# Molecular Formula Prediction for Chemical Filtering of 3D OrbiSIMS Datasets

**DOI:** 10.1021/acs.analchem.1c04898

**Published:** 2022-03-11

**Authors:** Max K. Edney, Anna M. Kotowska, Matteo Spanu, Gustavo F. Trindade, Edward Wilmot, Jacqueline Reid, Jim Barker, Jonathan W. Aylott, Alexander G. Shard, Morgan R. Alexander, Colin E. Snape, David J. Scurr

**Affiliations:** † Department of Chemical and Environmental Engineering, University of Nottingham, Nottingham NG7 2RD, U.K.; ‡ School of Pharmacy, University of Nottingham, Nottingham NG7 2RD, U.K.; § Innospec Ltd., Oil Sites Road, Ellesmere Port, Cheshire CH65 4EY, U.K.; ∥ National Physical Laboratory, Hampton Road, Teddington, Middlesex TW11 0LW, U.K.

## Abstract

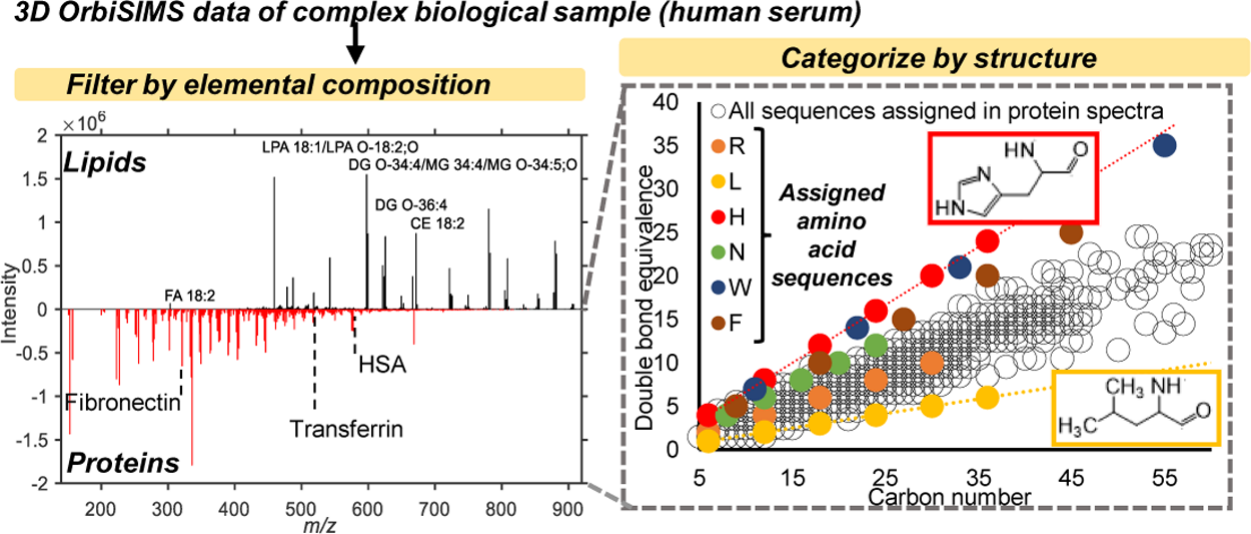

Modern mass spectrometry techniques produce a wealth of spectral data, and although this is an advantage in terms of the richness of the information available, the volume and complexity of data can prevent a thorough interpretation to reach useful conclusions. Application of molecular formula prediction (MFP) to produce annotated lists of ions that have been filtered by their elemental composition and considering structural double bond equivalence are widely used on high resolving power mass spectrometry datasets. However, this has not been applied to secondary ion mass spectrometry data. Here, we apply this data interpretation approach to 3D OrbiSIMS datasets, testing it for a series of increasingly complex samples. In an organic on inorganic sample, we successfully annotated the organic contaminant overlayer separately from the substrate. In a more challenging purely organic human serum sample we filtered out both proteins and lipids based on elemental compositions, 226 different lipids were identified and validated using existing databases, and we assigned amino acid sequences of abundant serum proteins including albumin, fibronectin, and transferrin. Finally, we tested the approach on depth profile data from layered carbonaceous engine deposits and annotated previously unidentified lubricating oil species. Application of an unsupervised machine learning method on filtered ions after performing MFP from this sample uniquely separated depth profiles of species, which were not observed when performing the method on the entire dataset. Overall, the chemical filtering approach using MFP has great potential in enabling full interpretation of complex 3D OrbiSIMS datasets from a plethora of material types.

## Introduction

The use of sophisticated mass spectrometry (MS) techniques with high mass resolving power yields a significant amount of chemical data from a given sample series and often a barrier to fully interpreting these datasets comes from annotating the spectral ions in order to identify the analytes. Calculations for automated peak assignment based on molecular formula prediction (MFP) are widely used in other areas of MS. Dedicated programs for processing these datasets exist such as MZmine 2,^1,2^ which uses MFP from accurate *m*/*z* values following rules laid out by Kind and Fiehn.^3^ Kew et al. recently developed software which uses MFP, followed by plotting species in filtered lists by their double bond equivalence (DBE) versus carbon number of each predicted formula. Plotting DBE versus carbon number to categorize chemical species with similar chemistries is a strategy which has been successfully applied in other areas of MS to deconvolute complex datasets, such as in petroleomics using Fourier transformed iso-cyclotron resonance MS,^4−6^ and dedicated software for processing these datasets exists such as “KairosMS”.^7^ DBE is a measure used to elucidate chemical structures from predicted molecular formula and determines the degrees of unsaturation in a molecule by calculating the ratio of C, H, N, P, and halogen atoms in the molecular formula. The DBE of a structure is contributed by either a double bond and/or a ring system; for example, benzene has a DBE of 4 (3 double bonds and one ring system) and cyclohexane has a DBE of 1. Where category databases exist for species (e.g., LIPID MAPS, PubChem and ChemSpider), the putative molecular and structural assignment can then be validated by cross-referencing to these.

Secondary ion mass spectrometry (SIMS) relies on detection of ions which have been liberated from a sample surface by bombardment from a primary ion source.^8^ While most MS techniques extract species from a sample using a solvent and then coarsely separate them using for example, chromatographic retention time, SIMS collects all ions from the surface. Therefore, these datasets contain molecular ions as well as fragments, meaning that assigning the parent species in highly complex samples typically requires sophisticated analysis methods. Software developed for other MS techniques regularly use molecular databases (libraries) to assign species, but the high level of fragmentation in SIMS means this is often unfeasible, meaning that “library-free” interpretations are needed. Green et al. showed that even with a mass accuracy of 1 ppm, up to 1000 formulae are possible at *m*/*z* 385, and concluded that even high accuracy SIMS techniques would need to be combined with filtering techniques for truly library-free interpretation of data from unknown samples, as well as extra steps such as isotopic distribution analysis.^9^ SIMS is also uniquely able to perform depth profiling and label-free chemical imaging of samples, but this additional dimensionality adds another layer of complexity to interpreting these datasets. Until recently, the low mass resolving power (<20,000) and mass accuracy and high levels of fragmentation of SIMS techniques were barriers to performing filtering of data in this way due to the vast number of possible formulae within acceptable errors for each ion.

The introduction of the 3D OrbiSIMS technique,^10^ with its superior mass resolving power (240,000 at *m*/*z* 200), sub parts-per-million mass accuracy, and reduced fragmentation using an argon gas cluster ion beam, now permits the use of chemical filtering approaches using MFP and DBE measures as applied in other fields of high mass resolving power MS techniques. The issue of fragmentation, while reduced, is still present, and the high amount of data is a barrier to full interpretation which carries the risk that key chemical insights or trends may be missed without this approach. Often, the complexity of SIMS data demands the use of sophisticated data analysis methods including unsupervised machine learning methods such as multivariate analysis (MVA), which has proven to be of great effectiveness for 3D structured SIMS data.^11,12^ However, most MVA methods suffer from bias toward high intensity ions, especially in very high dynamic range datasets typical of the 3D OrbiSIMS. Other tools include de novo sequencing of peptides and proteins, which was recently shown to be applicable to 3D OrbiSIMS data,^13^ but again suffers from the high level of data acquired from the technique and so a pre-processing workflow using chemical filtering would be highly valuable for these applications. To our knowledge, measures of MFP and DBE have not been applied to SIMS data. Therefore, a chemical filtering workflow using MFP to filter all secondary ions by predicted elemental compositions, followed by categorizing these ions by plotting their DBE versus carbon number would be useful to not only deconvolute and filter 3D OrbiSIMS data but could be applied to various data analysis tools as a pre-filter, such as MVA.

Here, we demonstrate the utility of chemical filtering using MFP and DBE measures on increasingly complex 3D OrbiSIMS depth profiling datasets from aluminum foil, a human serum sample containing chemically similar proteins and lipids and finally heterogeneous gasoline engine deposits. We applied MVA on filtered datasets from the deposit to showcase the advantage over performing MVA on raw 3D OrbiSIMS datasets.

## Methods

### Materials

#### Aluminium Foil

Standard aluminium foil (as available from any supermarket) was purchased and analyzed using 3D OrbiSIMS on both the shiny and dull side of the foil.

#### Engine Deposits

In this work, we analyzed the deposits in-situ on different gasoline direct injection fuel injector components from different vehicles, sourced from the USA. Two samples were fuel injector tips, which had a thick carbonaceous deposit, and one was a needle of the injector which exhibited a thin-film coating of deposit. Samples are termed injector tip 1, injector tip 2 and injector needle 1.

#### Model Protein Samples and Human Serum

Proteins: lysozyme from chicken egg white, α-chymotrypsin from bovine pancreas, insulin solution human, recombinant bovine serum albumin, horse skeletal muscle myoglobin, l-lactate dehydrogenase from rabbit muscle, human holo-transferrin, concanavalin A from jack bean, bovine plasma fibronectin, alcohol dehydrogenase from *Saccharomyces cerevisiae*, porcine lipase, bovine liver catalase, human serum albumin, and cytochrome c from equine heart were purchased from Sigma-Aldrich. Porcine pepsin and porcine trypsin were purchased from Promega. Serum from human male AB plasma, USA origin, sterile-filtered was purchased from Sigma-Aldrich. Proteins and the human serum were spotted and dried onto separate gold slides 3 times to obtain protein films.

### 3D OrbiSIMS

3D OrbiSIMS (Hybrid SIMS, IONTOF GmbH, Germany) analysis was conducted as outlined by Passarelli et al.^10^ Secondary ions were collected using the Q Exactive HF Orbitrap mass analyzer (mass resolving power of 240,000 at *m*/*z* 200). The mass scale was calibrated prior to each sample measurement using a range of silver cluster secondary ions sputtered from a silver sample using 30 keV Bi^+^ ions. In each case, samples were analyzed using single beam depth profiles using a 20 keV Ar_3000_^+^ gas cluster ion analysis beam which was defocused to 20 μm. In all cases, the Orbitrap injection time was 500 ms, and the automated gain control was switched off. The cycle time was 200 μs for all samples except the human serum sample, which was 400 μs, all were operated with a duty cycle of 4.4%. Charge compensation was achieved with a low energy electron flood gun (2.3 A filament current and extraction bias of −20 V) and by regulation of the main chamber with argon gas (9 × 10^–7^ mbar) to delocalize any accumulation of charge surrounding the sample. A flow of pressurized nitrogen was fed to the Orbitrap at 12 bar. Sample data were collected over a mass range of *m*/*z* 75–1125 for all samples except the human serum sample which was measured in the mass range of *m*/*z* 150–2250, with the application programming interface provided by Thermo Fisher for control of the Orbitrap MS portion of the instrument. Data processing was performed using SurfaceLab Version 7.1.c (ION-TOF GmbH).

Engine deposit samples were analyzed in the negative polarity mode; the analysis area was 200 μm^2^ with an interlaced border (random raster mode). Measurement times for the samples were as follows: injector tip 1, 22,094; injector tip 2, 128,000; and injector needle 1, 561 s. Primary ion currents were as follows: injector tip 1, 238 pA; injector tip 2, 260 pA; and injector needle 1, 230 pA. The human serum sample was analyzed in the positive polarity mode over a 200 μm^2^ area (random raster mode) using 3D OrbiSIMS single beam depth profiles as described in the data acquisition section. The measurement lasted 30 scans (approximately 40 s measurement time), and the primary ion current was 218 pA. Aluminium foil was analyzed using 3D OrbiSIMS single beam depth profiles, as described in the data acquisition section. Data were collected in negative polarity in an analysis area of 150 μm^2^ (random raster mode), and the measurement time was 134 s.

### DBE Calculation

DBE relates the number of double bonds or rings in a molecule and is calculated using the following formula in eq 1 which relates to elemental formula as
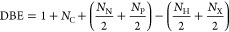
1where *N*_C_ = the number of carbon atoms, *N*_H_ = the number of hydrogen atoms, *N*_X_ = the number of halogen atoms (Br, Cl, I and F), *N*_N_ = the number of nitrogen atoms, and *N*_P_ = the number of phosphorus atoms. Isotopes for C, Br, and Cl are considered. It is important to note that non-integer DBE values are generated, which arises from the inclusion of [M ± H] ions. Therefore, the inclusion of these non-integer DBE values is necessary and the true value of DBE needs to be considered by the user, particularly when considering positive ions which can exist as [M + H]^+^ and [M]^•+^ ions.

### Data Analysis

The chemical filtering method for interpreting 3D OrbiSIMS data laid out in this work was performed using SIMS-MFP software which was developed in MATLAB and is available for use in this work, a description of the software functions is given in Supporting Information Note S1 and the graphical user interface is shown in Supporting Information Figure S1. Briefly, the software requires input of a depth profiling dataset as a .txt file format or as a matrix in MATLAB. To do this, we first performed a peak search on raw data, a minimum intensity threshold was manually determined by discerning the minimum count of a peak that distinguished it from a noise peak (this value varied between samples). For the engine deposit samples, the peak search generated a file containing ion peak data for 13,437 ions for injector tip 1, 12,707 ions for injector tip 2 and 1600 ions for injector needle 1. The aluminium foil file contained 1252 ion peaks, and the serum sample had 5805 ion peaks. MFP is then performed to produce spectra of filtered ions based on elemental compositions in the “search constraints”, namely, the maximum error of each ion and the minimum and maximum value of each element in the molecular formula. The DBE value of predicted formula of each ion is calculated and plotted against its carbon number to allow observation of different trends in the data by the level of unsaturation in different molecules. More information on the method can be found in Supporting Information Note S1. To advance this methodology, we propose performing MVA on the filtered datasets (depth profiles or spectra) to further elucidate differences in the trend of each sub-group. We apply all these features to a range of different samples which were analyzed by 3D OrbiSIMS in subsequent sections. MVA—specifically non-negative matrix factorization (NMF) was performed using SIMS-MFP software which is linked to SIMS-MVA algorithms.^11^ In each case, the optimal number of factors was determined by performing NMF using different numbers of factors until excessive repetition of endmember loadings was observed. In each case the number of iterations was 200.

## Results and Discussion

### Differentiation of Contaminant Overlayer and Aluminum Foil Substrate

**Figure 1:**
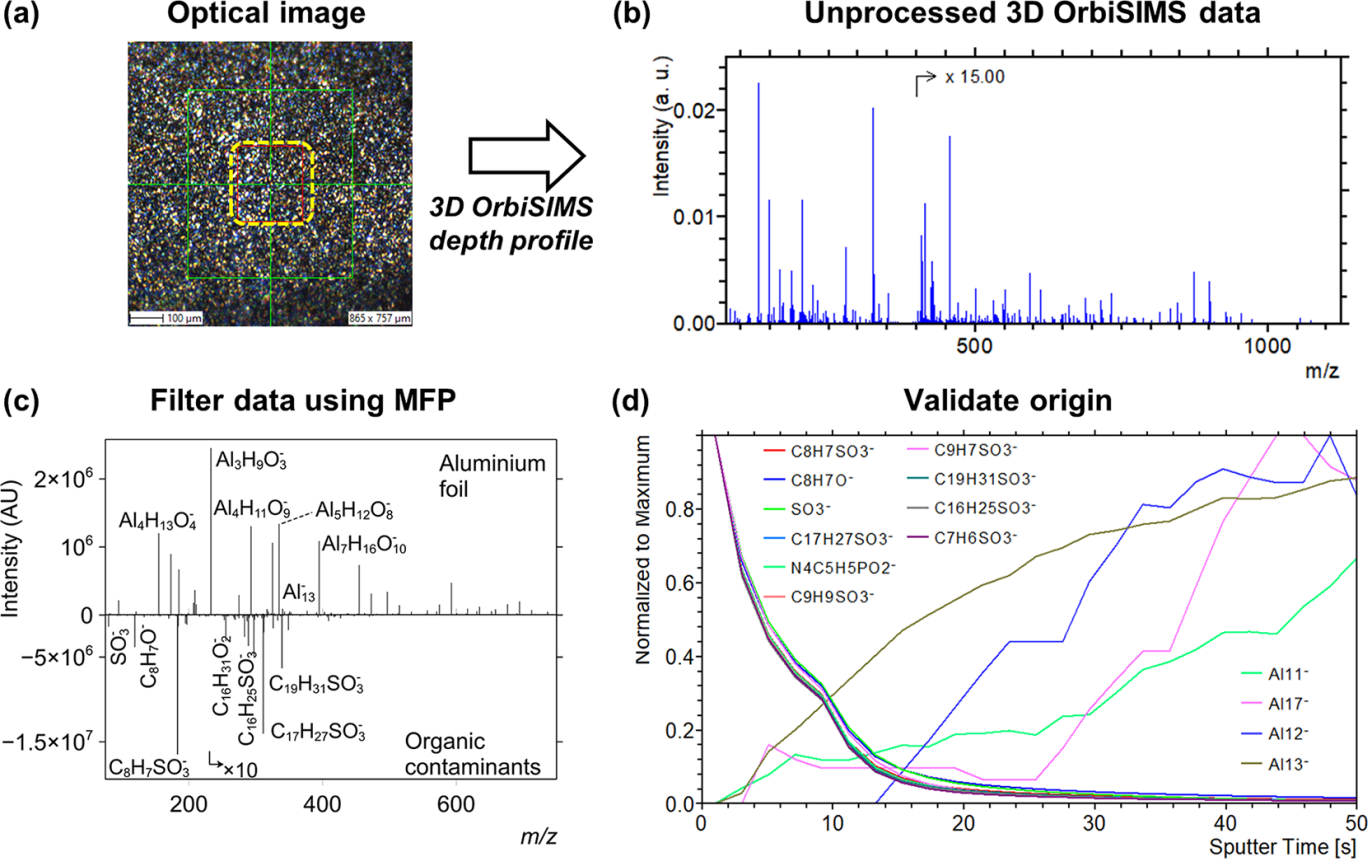
Separating organic and inorganic species on aluminum foil 3D OrbiSIMS depth profiling datasets using MFP. (a) Optical image of the analysis area before Ar_3000_^+^ depth profiling. (b) Raw depth profile accumulation spectra in the negative ion mode. (c) Positive scale, plots of the inorganic species containing Al, O, and H identified using MFP. Negative scale, contaminants identified after performing a repeated mass separation of the initial dataset, followed by another formula filtering iteration analysis to identify species with C, H, O, N, S, and P (intensity × −1). (d) Depth profiles of some species in each sub-group. MFP, molecular formula prediction.

Here, we apply chemical filtering to data from aluminum foil, which is regularly used in surface and materials analysis, and is considered a very clean material but may present trace organic contaminants which would be useful to assign and ascribe to an origin. MFP using elemental and mass error constraints were set, specifically a maximum deviation threshold of 2 ppm for ions >*m*/*z* 95 and 3 ppm for ions <*m*/*z* 95, which are typical acceptable errors when interpreting Orbitrap datasets.^14^ After automatically annotating aluminum peaks (including oxides and hydroxides) using MFP, we performed another search for organic species on the “unassigned” dataset (Figure 1). This is a useful test sample as the species we are distinguishing are chemically distinct and are expected to have characteristic depth profiling behavior which can validate our findings. This approach can be used to help uncover the identity and source of contamination or identify miss-assignments to either the substrate or contaminant category.

The 3D OrbiSIMS depth profile of aluminum foil (Figure 1a) yielded a raw spectrum with dominant signals for inorganic ions (Figure 1b). The first search using MFP was for species containing Al, O, and H (any values of each element were considered). Out of a peak list containing 1252 ions, MFP identified 408 corresponding to Al, O, and H species (Supporting Information Table S2) which are plotted in the “aluminum foil” spectra in Figure 1c. We then performed MFP again on the discarded dataset (of which no ions could be assigned to the foil), with elemental constrains of C_*n*_, H_*n*_, O_20_, N_0-10_, S_1_, and P_0-2_. Filtered spectra of these ions in the contaminant search are shown in the lower plot in Figure 1c (intensity × −1), in total there were 920 possible formulae for ions which can be found in the data repository. The most intense ions all corresponded to sulfonated compounds (containing an SO_3_^–^ group), and we identified 53 such ions with various double bond equivalencies, suggesting degrees of unsaturation or aromatization. Organic sulfonates such as these have been ascribed to lubricating oil in previous SIMS analysis,^15^ we attribute these ions as originating from the manufacturing of the foil where lubricants are used in the machinery which comes into contact with the foil.^16^ We applied the filter to a dataset taken from the “shiny” side of the foil which showed only 31 sulfonated species and all with lower normalized intensity (Supporting Information Table S2), which is expected as the “shiny” side of the foil comes into less contact with machinery in the manufacturing process. We also applied MATLAB code which matched the formulas generated by MFP to ions found in the LIPID MAPS database.^17^ Out of the list we identified 14 ions on the dull side of the foil, 10 of which were fatty acids and 4 were assigned as sterols, both of which may derive from trace contamination from skincare products.^18^ Aside from these, we also identified hydrocarbon fragments and a summary of all species in the mentioned compound classes can be found in Supporting Information Table S2.

Application of chemical filtering to data from solvent-based MS techniques is limited to only distinguishing ions by their formula, but the use of depth profiling in 3D OrbiSIMS uniquely allows further categorization of data by their depth behavior and can help confirm their origin. Ions with increasing intensity as a function of sputtering dose may be ascribed to the substrate and vice versa for organic contaminants (Supporting Information Figure S2). Depth profiles of the most intense aluminum species showed two trends, where ions containing only Al increased in intensity in the experiment (example ions are shown in the profile in Figure 1d), the aluminum oxide/hydroxide ions decreased in intensity, suggesting the presence of an oxide layer. The purely organic species decreased in intensity slightly faster than the metal oxides (Supporting Information Figure S2).

Chemical filtering on this sample dataset was useful as an initial test using a system containing elementally distinct species, that is, organic and inorganic materials. Furthermore, the anticipated depth profiling behavior of each system enabled a validation for the species that MFP had categorized. We note that an alternative approach could be to search for organic species first; however, the simpler approach was determined to identify aluminum species initially as there are fewer possible combinations of molecular ions. This approach also shows the flexibility to filter data by first searching for known species and separating them and repeating the process to elucidate unknown chemistries in a sample.

### Distinguishing Proteins and Lipids in a Human Serum

**Figure 2:**
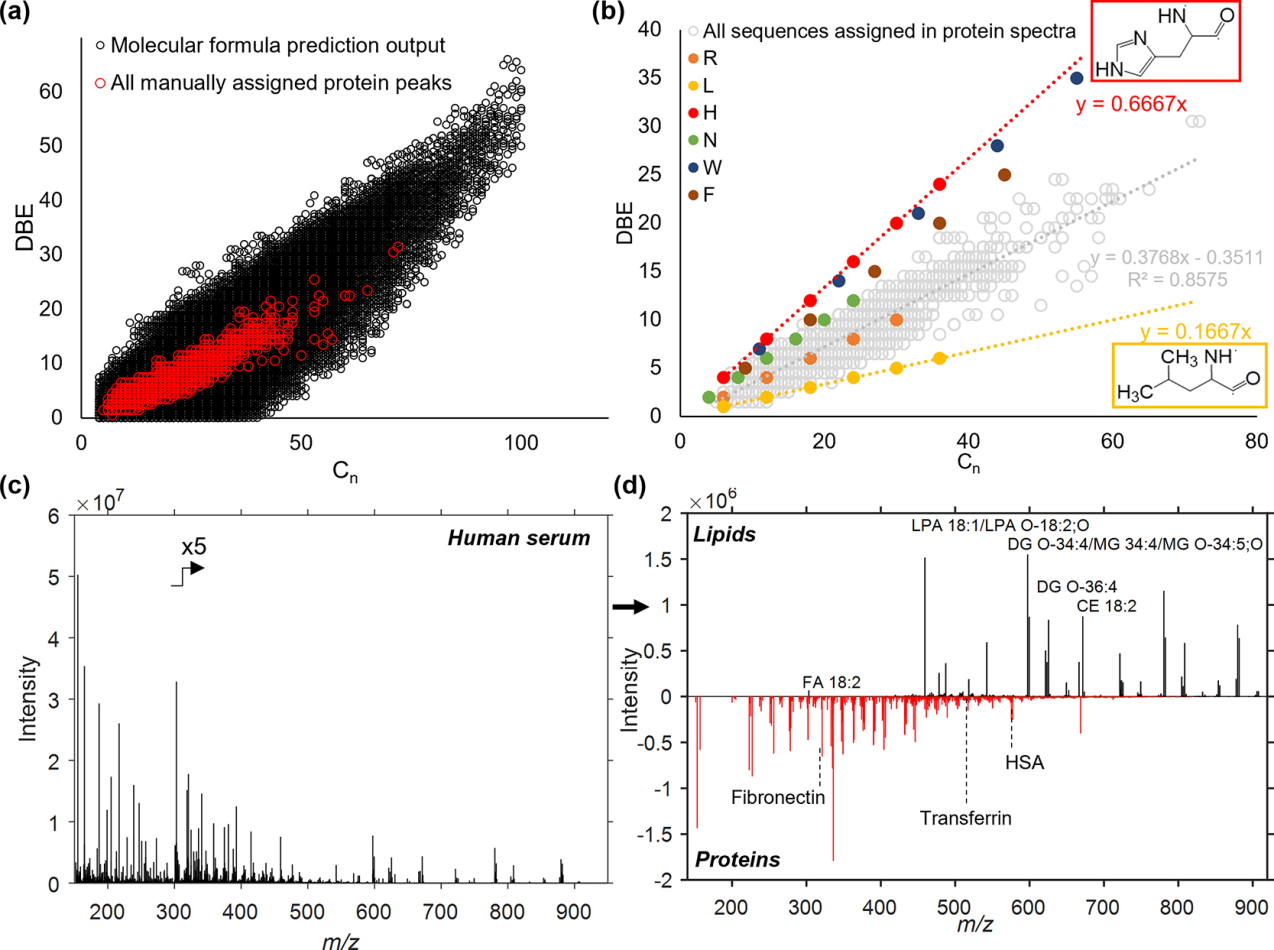
(a) Comparison of all assignments proposed by MFP in a collated peak list of all manually assigned protein peaks in 16 different protein samples (black) and only correct protein assignments (red). (b) All protein peaks assigned in 16 protein samples are widely spread around the trend line of DBE/C_*n*_ (grey). The variability of DBE/C_*n*_ of protein assignments is caused by different amino acid DBE/C_*n*_ (orange—asparagine, R; yellow—leucine, L; red—histidine, H, green—asparagine, N; brown—phenylalanine, F). (c) Human serum sample is too complex to manually assign fragments of biological molecules. (d) MFP combined with the LIPID MAPS database automatically assigns lipid groups (black). Additional MFP on the rest of the spectrum enables assignment of proteins in serum (red). Peaks assigned as fragments of fibronectin (C_15_H_22_N_4_O_3_Na^+^, *m*/*z* 329.1585), transferrin (C_24_H_44_N_6_O_5_Na^+^, *m*/*z* 519.3258), and human serum albumin (C_24_H_40_N_8_O_7_Na^+^, *m*/*z* 575.2918). Chemical filtering was carried out on SIMS-MFP software.

In the previous example, we distinguished species with very different elemental compositions. In order to further challenge the chemical filtering approach and to introduce the concept of filtering them by their DBE value, we apply the method to a human serum sample, containing proteins and lipids. Interpretation of 3D OrbiSIMS spectra of proteins is challenging, as the fragmentation process produces multiple amino acid sequences, each occurring as different types of ions (a, b, c, y, and z ions).^13^ The limits applied to the elemental formula were based on manually assigned protein fragments from 16 protein samples reported by Kotowska et al.: C_4-100_, H_8-200_, N_0-20_, O_0-20_, S_0-1_, Na_0-1_. MFP returned 58,135 possible assignments based only on the elemental composition in a collated peak list of all manually assigned protein peaks (Figure 2a, black). Among all assignments suggested by MFP, we highlighted the known structures (Figure 2a, red). Due to proteins being composed of different combinations of 20 amino acids, single DBE values or DBE/C_*n*_ are not suitable for further filtering of the data. Depending on the amino acid composition of detected protein fragments, the DBE/C_*n*_ is placed between the amino acid of the highest (Figure 2b, histidine, H, red) and lowest DBE/C_*n*_ (Figure 2b, leucine, L, yellow). The peak list exported from the spectrum contains 5805 peaks and providing a full interpretation of unprocessed data is not feasible. This is especially the case when targeting fragments of macromolecules such as proteins, which are typically of relatively weak intensity and are easily overlooked (Figure 2c). The spectrum of serum is dominated by readily ionized lipids. Therefore, the first step of the processing was to filter the lipid-related peaks from the data using MFP. We used the LIPID MAPS database of “bulk” lipid species^17^ and wrote code which automatically matches formula in the list from MFP to lipids in the database (available upon request). The database groups major classes of lipids based on the molecular formula and indicates general classification of a lipid but not specific chain positions or double bond regiochemistry and geometry. The formulae suggested by SIMS-MFP were matched with the database to automatically assign the lipid peaks in the human serum spectrum. As a result of this matching, possible assignments for 226 lipid peaks were generated (Figure 2d, black, and Supporting Information Table S2). Peaks assigned as lipids were removed from the dataset using the “separate” function in the software which can generate a list of ions that were not assigned in the original formula prediction search. MFP was run again on the “unassigned” peaks to aid assignment of protein peaks. The search found 69,602 possible formulas based on the allowed elemental composition. A database of up to 6-membered peptides was calculated using known formulas of each amino acid. A total of 3328 assignments were found to match with the elemental composition of sodiated or protonated protein fragments gathered in this database (Figure 2d, red). Example protein assignments and possible amino acid assignments are listed in Supporting Information Table S3. Molecular formulae of protein fragments obtained using MFP, together with de novo sequence search, described in previous work,^12^ enabled automatic assignment of sequences of abundant serum proteins such as serum albumin, transferrin, and fibronectin on filtered datasets (Supporting Information Table S4). Additionally, phosphate salts were assigned in the serum spectrum by searching compounds of composition: C_0_, H_1-10_, N_0-0_, O_0-10_, S_0-1_, P_0-5_, Na_0-5_, Ca_0-5_, and Cl_0__-5_ in the list of peaks unassigned as either lipids or proteins (Supporting Information Table S5). Overall, filtering using MFP and grouping of species by DBE significantly reduced the complexity of the 3D OrbiSIMS human serum spectrum by separating and assigning secondary ions associated with lipids from fragments of large biological molecules such as proteins.

### Using MFP as a Pre-filtering Tool to MVA Techniques on 3D OrbiSIMS Data from Carbonaceous Engine Deposits

**Figure 3:**
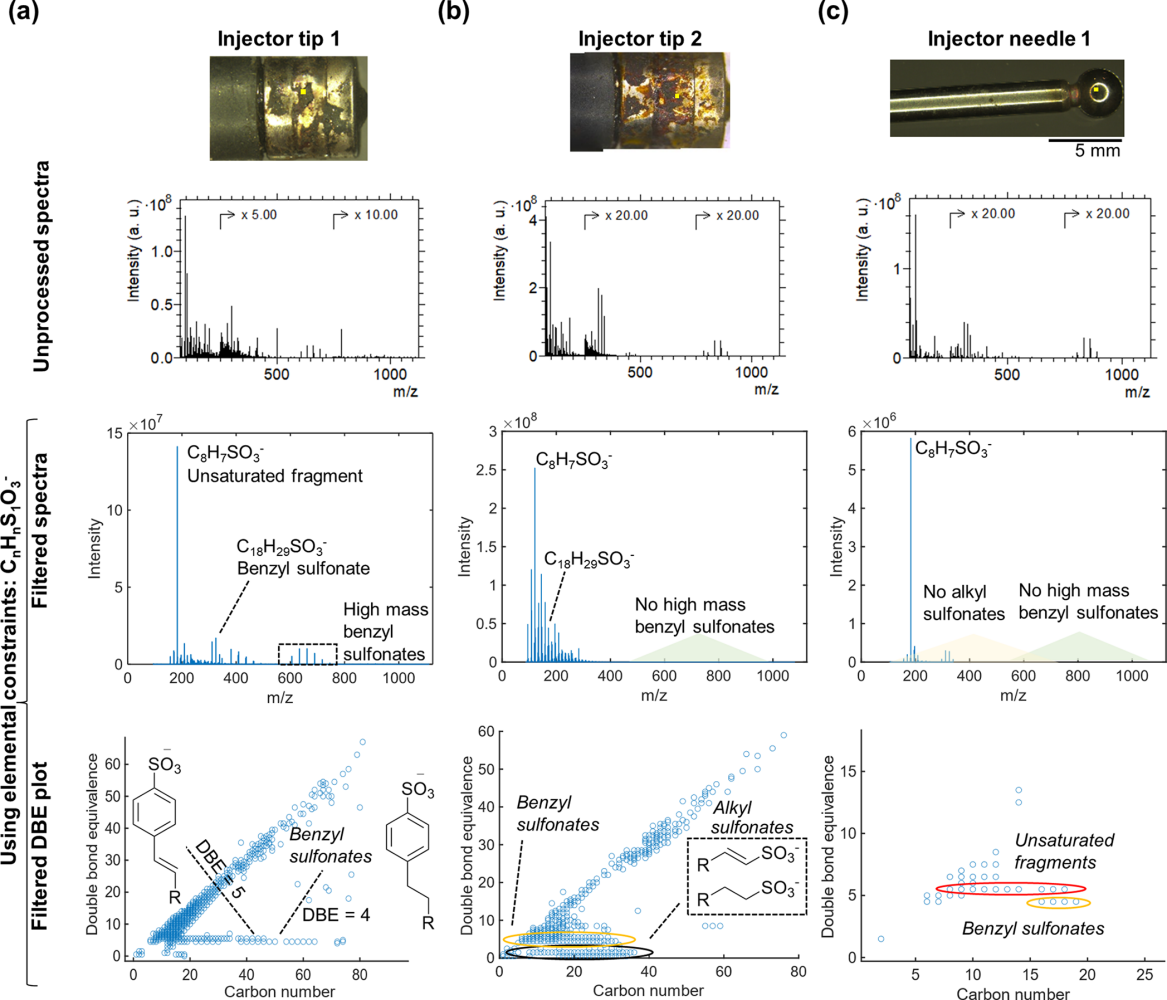
Using MFP to search for sulfated species in gasoline engine deposits. In each case, the raw 3D OrbiSIMS depth profile accumulation spectrum is shown above the spectrum of sulfated ions and the lowest is the filtered DBE plot of these species. (a) Deposit on injector tip 1, showing benzyl sulfonates present up to high masses (C_75_). (b) Injector tip deposit 2, showing benzyl sulfonates <C_40_ and a unique distribution of alkyl sulfonates with a DBE value <2. (c) Results from the injector needle, showing only benzyl sulfonates but to a lower maximum mass than other samples.

The final application of our chemical filtering approach is to gasoline engine deposits, which are the most challenging samples to interpret in this work owing to the similarity of chemical species it contains. In addition, they are heterogeneous samples and previous work found that most species had different depth profiling behavior.^15^ We employed MFP and DBE filtering to depth profiling data from several samples to interrogate these complex datasets and focused on key classes of compounds, followed by MVA on the filtered data. The first type is sulfated species, which can be indicative of contamination of lubricating oil in engines.^15^ Filtered spectra and DBE plots from the engine deposit samples after searching for species with compositions of C_*n*_H_*n*_S_1_O_3_^–^ are shown in Figure 3.

Filtered DBE plots allowed us to make distinctions between sample chemistry that would have remained lost in the high amount of chemical data in the raw spectra without the use of MFP. In all samples, we easily identified benzyl sulfonates (DBE = 4), depicted in Figure 3a, including the cited parent ion (C_18_H_29_SO_3_^–^).^19,20^ An intense SIMS-induced fragment ion (C_8_H_7_SO_3_^–^, DBE = 5) was also in all filtered spectra and DBE plots (Figure 3a–c), which has been confirmed previously using 3D OrbiSIMS and MS/MS.^15^ Benzyl sulfonates with masses higher than the cited parent ion were present in both injector tip deposits, termed “high mass benzyl sulfonates” (Figure 3a,b), which extended up to much higher masses in injector tip 1 (C_78_) but were not present in the injector needle deposit (Figure 3c). High mass sulfonates have been identified and confirmed with MS/MS in previous work and are attributed to the product of reactivity of the deposited residue in the engine itself. Understanding the extent of this reactivity by visualizing that maximum mass of ions is important in unraveling key deposit formation mechanisms.^15^ The filtered DBE plot also highlighted a unique series in injector tip 2 with a DBE of 1 and 2 which we putatively assign as alkyl sulfonates which have not been identified in these systems before. Their low intensity compared to benzyl sulfonates meant that without the visualization approach they were unidentified in our previous analysis of these sample types.^15^ In total 410, 353 and 35 sulfated ions were detected in injector tip 1, injector tip 2 and injector needle 1, respectively. The comprehensive nature of this categorization of species is evidently a useful feature and is shown in the tabulated output of oxygenated ions found in all samples in Supporting Information Table S6. Depth profiles of the 10 most intense ions all showed consistent behavior which further validates grouping of these assignments into this compound class (Supporting Information Figure S3).

**Figure 4:**
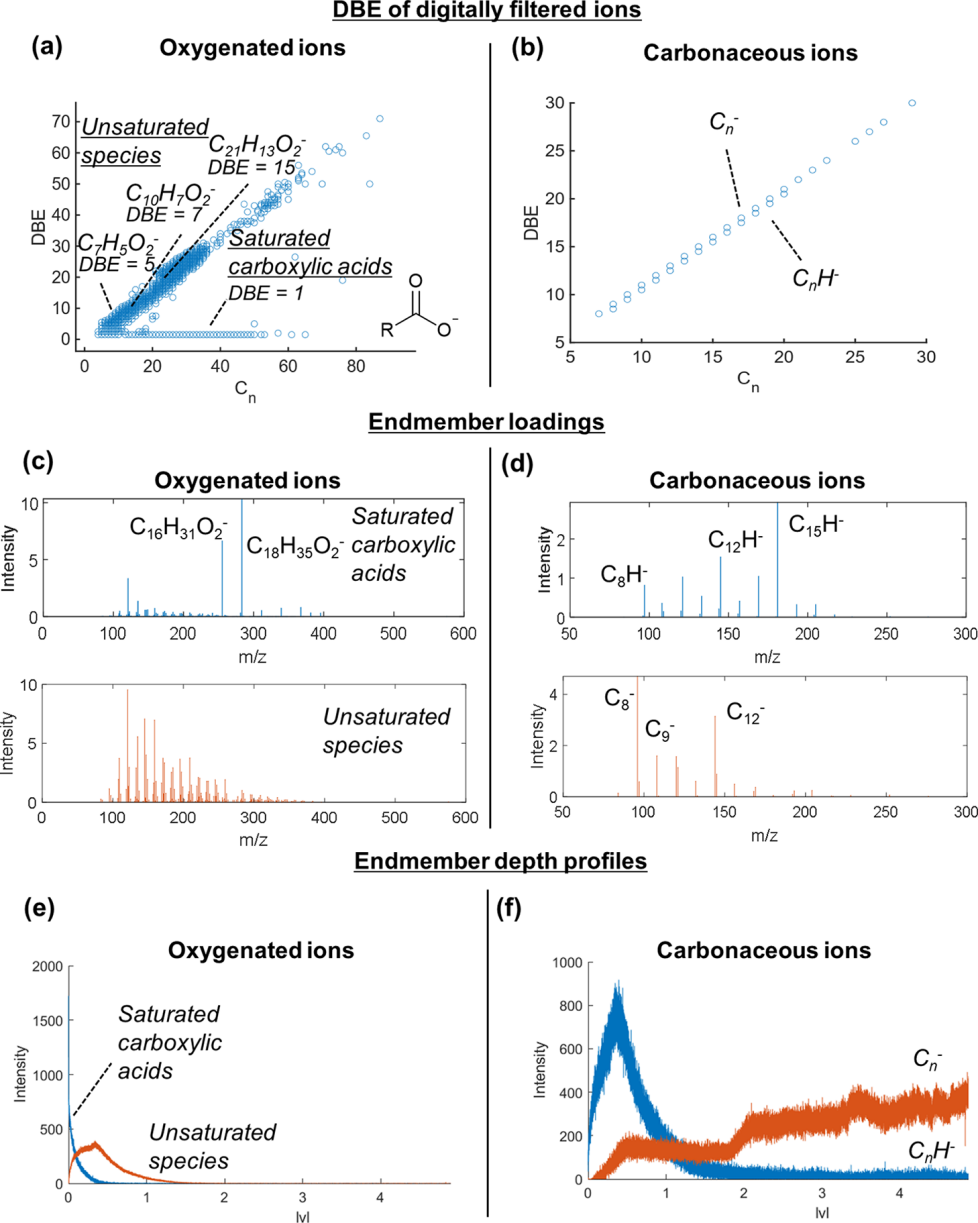
Demonstrating MVA on datasets after performing MFP on 3D OrbiSIMS depth profiling data from injector tip 1 deposit. (a) DBE versus carbon number plots of all oxygenated ions (C_*n*_H_*n*_O_2_^–^) identified using MFP. (b) DBE versus carbon number plots of all carbonaceous ions (C_*n*_H_<1_^–^) identified using MFP. (c) Loadings of oxygenated ions after performing NMF on the filtered list of oxygenated ions identified using MFP. (d) The first two loadings of carbonaceous ions identified using MFP. (e) Depth profiles of the first two endmembers of the oxygenated ions. (f) Depth profiles of the first two endmembers of the carbonaceous ions.

The next two key compound classes discussed are oxygenated species containing possible carboxylate functional groups (C_*n*_H_*n*_O_2_^–^) and carbonaceous ions (C_*n*_ and C_*n*_H^–^) and using MFP we identified possible ions. To interrogate the depth profile behavior and further categorize these species, we next performed MVA on the filtered list of ions; outputs from these steps are shown in Figure 4 with data taken from injector tip 1.

In total, 522 discrete oxygenated ions were automatically identified which fit the criteria (with no other option within the deviation threshold) (Supporting Information Figure S4). DBE plots showed different trends of unsaturation of ions, and we easily identified saturated carboxylic acids by their DBE value of 1.5 (which corresponds to a DBE of 1 for the neutral ion if we assume [M – H]^−^ ions were generated) (Figure 4a). The depth profiles of the top 10 most intense ions in this filtered list showed separation into two distinct behaviors, one for species with a higher DBE (>5) and one for saturated species (Supporting Information Figure S4), which we attribute to carboxylic acids. We repeated MFP of oxygenated ions for the two other deposits; injector tip 2 also contained saturated carboxylic acids and the thin film deposit on the injector needle did not contain any long chain carboxylic acids (Supporting Information Table S6).

We annotated 34, 52, and 19 carbonaceous ions in injector tip 1, injector tip 2, and injector needle 1, respectively (Figure 4b and Supporting Information Table S6). Depth profiles of the most intense ions revealed subtle differences in depth behavior where purely carbonaceous (C_*n*_^–^) ions had higher intensity at the lowest deposit depth compared to hydrogenated ions (C_*n*_H^–^) (Supporting Information Figure S5). This aligns with our hypothesis from previous work of the origin of these ions being condensed carbonaceous clusters which increase in C/H ratio in lower deposit depths.

MVA on depth profiling data in theory negates the need to interrogate each ion in a dataset; however, applying MVA on complex SIMS data often leads to issues such as overfitting of data in output loadings, replicating intense ions in multiple loadings and generally under-performing in discerning ions with subtle differences in their spatial distributions. To demonstrate the utility of MVA on chemically filtered data, we performed NMF analysis of the depth profiles of oxygenated and carbonaceous ions annotated using MFP. This is of particular importance when considering the oxygenated ions, which still contained >500 ions in its filtered dataset, so interrogating each one would be unfeasible. NMF (described in the methods) analysis on the dataset was performed with two factors on the list of oxygenated ions after MFP was performed. This clearly separated into two depth profile behaviors (Figure 4c), one corresponded to saturated carboxylic acids, and one corresponded to unsaturated ions. Of note is that this is comparable to the trends identified by the levels of unsaturation in the DBE plots (Figure 4e).

NMF output using two factors on the dataset of carbonaceous ions after MFP also showed distinction into the two depth profiling trends identified by manual peak picking (Figure 4f). This is despite these ions only having a subtle difference in depth profile behavior and only one proton difference in composition (Figure 4d). NMF on the filtered dataset from the injector needle did show a similar trend shown by higher scores of purely carbonaceous ions for the component profile which increased at lower depths (Supporting Information Figure S6). This is despite the analysis time being significantly shorter (561 s on the needle and 22,094 s on the tip), suggesting the depth profiles reflect real chemical differences and is not an effect of long sputter times. This will be explored further in future work but does highlight the importance of depth profiling in assigning the origin of species in MS data.

To illustrate the advantage of filtering the data prior to MVA, we performed NMF on the unfiltered 3D OrbiSIMS dataset to attempt to recreate the MVA outputs from the three classes discussed so far (sulfated, oxygenated and carbonaceous ions). Profiles of the first 3 factors followed general trends of these classes, but many ion peaks were present in multiple loadings. Moreover, it failed to distinguish subtle differences in the profiles of sulfated, oxygenated, or carbonaceous ions (Supporting Information Figure S6), for example, it did not separate C_*n*_^–^ and C_*n*_H^–^ ions, which was achievable when using NMF after performing an initial filtering using MFP (Figure 4f). We increased the number of endmembers to four to try and discern more subtle differences, but again, we observed overfitting of loadings and an entire loading dataset was replicated without revealing the subtle differences in profile trends. Overall, this shows the utility of performing MFP followed by MVA on filtered datasets to unveil subtle chemical trends in complex heterogeneous samples.

The full chemical filtering method described in this work was carried out by a piece of software, SIMS-MFP, which was developed using MATLAB and has a dedicated graphical user interface described in Supporting Information Note S1. The software is freely available as part of this work (https://github.com/medney96/SIMSMFP), and we believe it will be of great use to many users in the SIMS community whether performing MFP or in addition to other data analysis tools such as MVA or de novo sequencing. Finally, we propose chemical filtering can be applied to other SIMS techniques including time-of-flight SIMS, including MS/MS data, to further refine possible annotations per peak, making our approach applicable in some format to all SIMS datasets.

## Conclusions

The need for automated tools for MS data analysis is ever growing, particularly with the development of high mass resolving SIMS techniques such as 3D OrbiSIMS. This work successfully applies a new methodology to filter and deconvolute 3D OrbiSIMS datasets using MFP and DBE measures for the first time. Species from adventitious organic contamination upon aluminum foil were readily filtered and separated from the substrate peaks, and the origin of the contamination was ascribed to lubricating oil species from its manufacturing process. In a complex biological sample, human serum, we automatically matched 226 putatively assigned molecular and fragment lipid ions with the LIPID MAPS database. Further filtering of serum data by applying a protein fragment database aided amino acid sequence assignment by reducing the number of non-protein related peaks. In carbonaceous deposits formed on metal in engines, we identified known chemistries such as carboxylic acids, elucidated species not observed previously (alkyl sulfonates), and highlighted differences between samples from the filtered DBE plots which were missed from an initial targeted analysis due to the low intensity of key ions. Depth profiles helped to validate groupings of species and we showed how performing unsupervised machine learning and de novo sequencing techniques on filtered datasets gave superior distinction of sample chemistries in layered systems compared to applying the techniques on the unfiltered dataset.

There are many useful applications of the filtering approaches we have demonstrated, particularly in assigning components in complex mixtures, such as the adsorbed biomolecular layer of implanted biomaterials and the influence of the biointerface on biofilm formation with importance in infection. We have demonstrated here that the filtering approach allowed us to make assignments which we could not have made without this tool, yet we note that we have not completely mitigated challenges inherent with SIMS of these complex samples such as that within a 2 ppm window there are often many possible assignments for high mass species. This was evident in the human serum where even after removing lipid peaks and then filtering the resultant dataset by elemental composition we still had >3000 possible protein assignments to those peaks we classify as likely peptides. A SIMS database of protein fragments could help identify the proteins by limiting number of peaks for amino acid sequence search. But even for peaks that are automatically matched in databases, there will need to be structural assignments made using MS/MS, which is currently time consuming. Alternatively, it is alluring to envisage digitally predicting which peptides will be seen in SIMS, in an analogous way as the proteomics community has developed for liquid chromatography MS. That being said, without the filtering method it would have been impossible to generate the >3000 possible protein assignments, and our method will no doubt reduce data analysis time and help unveil new chemical insights from even the most complex 3D OrbiSIMS datasets.

## Accession Codes

The SIMS-MFP MATLAB application can be accessed at https://github.com/medney96/SIMSMFP. Raw 3D OrbiSIMS data can be accessed at The University of Nottingham data repository at http://doi.org/10.17639/nott.7168.

## Abbreviations

3D OrbiSIMS: 3D orbitrap secondary ion mass spectrometry
DBE: double bond equivalence
FT-ICR: Fourier-transformed iso-cyclotron resonance
MFP: molecular formula prediction
MVA: multivariate analysis
NMF: non-negative matrix factorization
SIMS: secondary ion mass spectrometry
