# Supplementary material for: Molecular Formula Prediction for Chemical Filtering of 3D OrbiSIMS Datasets
Source: Anal Chem. 2022 Mar 11;94(11):4703–11. doi: 10.1021/acs.analchem.1c04898 (PMC8943605; doi:10.1021/acs.analchem.1c04898)
Supplement: Supplementary file 1 — ac1c04898_si_001.pdf [file ac1c04898_si_001.pdf]

Supplementary Information for

Molecular Formula Prediction for Chemical Filtering of 3D OrbiSIMS Datasets

Max K. Edney,[1] Anna M. Kotowska,[2] Matteo Spanu,[1] Gustavo F. Trindade,[2,4] Edward Wilmot,[3] Jacqueline Reid,[3] Jim Barker, [3] Jonathan W. Aylott,[2] Alexander G. Shard,[4] Morgan R. Alexander,[2] Colin E. Snape[1] & David J. Scurr[2]

[1]*Department of Chemical and Environmental Engineering, University of Nottingham, Nottingham, NG7 2RD, UK*

[2]*School of Pharmacy, University of Nottingham, Nottingham, NG7 2RD, UK*

[3]*Innospec Ltd., Oil Sites Road, Ellesmere Port, Cheshire, CH65 4EY, UK*

[4]*National Physical Laboratory, Hampton Road, Teddington, Middlesex, TW11 0LW, UK*

Table of Contents:

Supplementary Notes:

Supplementary Note 1: SIMS MFP software graphical user interface......S2

Supplementary Figures:

Supplementary Figure 1: Interface of SIMS-MFP software showing all possible outputs......S2

Supplementary Figure 2: 3D OrbiSIMS depth profiles of organic and inorganic species on aluminum foil......S4

Supplementary Figure 3: 3D OrbiSIMS depth profiles of intense sulfated species in engine deposit 1......S5

Supplementary Figure 4: Application of MFP to identify carboxylic acids in engine deposits......S6

Supplementary Figure 5: Application of MFP and MVA on carbonaceous ion datasets in engine deposits......S7

Supplementary Figure 6: NMF analysis on the raw 3D OrbiSIMS data from engine deposit 1 with different conditions......S9

Supplementary Tables:

Supplementary Table 1: Output data file from SIMS-MFP of molecular formula which fit the constraints of from the searches of the aluminum foil and organic contaminants......S10

Supplementary Table 2: Lipid group assignments made automatically in the human serum sample......S22

Supplementary Table 3: First 60 of 3,328 peaks assigned in human serum as potential protein fragments......S28

Supplementary Table 4: Assignments of abundant serum proteins, albumin, transferrin and fibronectin......S30

Supplementary Table 5: Assignments of 50 most abundant salt ions in the human serum spectrum......S31

Supplementary Table 6: Output from SIMSMFP on carbonaceous ions ($C_nH_{<1}^-$) found in negative ion depth profile datasets from engine deposits......S33

Supplementary Table 7: Output from SIMS-MFP on all species containing two oxygen atoms found in negative ion depth profiles from engine deposits......S35

Supplementary Table 8: Output from SIMS-MFP on all sulfonated species found in negative ion depth profile datasets from engine deposits......S59

Supplementary note 1 - SIMS-MFP software graphical user interface

The need to simplify high-resolution 3D SIMS datasets is ever-growing in the field, SIMS-MFP software combines tools for MFP, categorization of species by double bond equivalence (DBE) and integrates existing MVA tools which can be linked to existing SIMS-MVA software.[9] A unique feature of this program is its incorporation to SIMS depth profiling data and the graphical user interface (GUI) (**Supplementary Figure 1**) is to display the profiles in both the pre and post filtered list and throughout this work we show its use for validating the grouping of secondary ions and is discussed in this work.

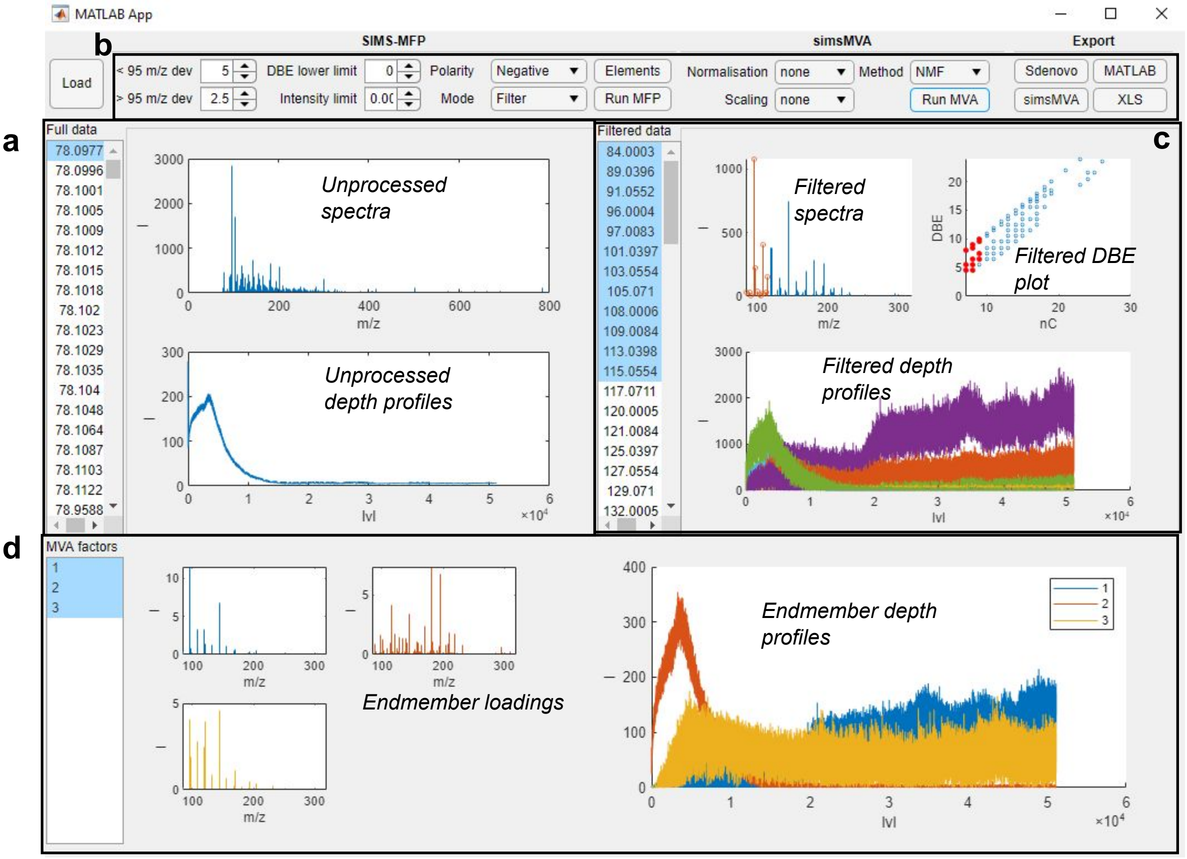

Supplementary Figure 1*. Interface of the SIMS-MFP software showing key steps for the workflow an example 3D OrbiSIMS dataset. A. Display of the pre-filtered data including raw spectra and profiles of selected species. B. Inputting search criteria, error thresholds, DBE and intensity lower limits, polarity and the elemental compositions ('elements' button must be selected). C, After selecting 'Run MFP' the output data is shown. The filtered spectra of ions identified (left) and DBE versus carbon number of ions found in the search are displayed. Depth profiles for species can be selected also. D. The result of 'Run MVA' on the filtered depth profiling data. Data output is shown after performing MVA on the filtered list.*

Upon loading a selected depth profile matrix by selecting the 'Load' button, the software displays the peak list and profiles for each ion in the list (**Supplementary Figure 1a**). Next, the user enters pre-set criteria for the filter, namely elemental compositions by clicking the 'elements' button (**Supplementary Figure 1b**), which brings up a

table where maximum and minimum values are chosen, an untargeted approach can be used by selecting '-1' in the maximum value. Other constraints can be determined such as minimum DBE value, maximum error threshold (we recommend < 2 ppm for ions > *m/z* 95) and minimum peak intensity which are selected using the buttons in **Supplementary Figure 1b**. After selecting the 'Run MFP' button the program will run and cycle through each *m/z* value in the list and attempt to calculate its formula based on the preset criteria and will calculate DBE on proposed molecular formula. The program generates several plots (**Supplementary Figure 1c**). Firstly, a mass spectrum containing only ions with formula meeting the preset criteria (termed 'filtered spectra'). Second, a plot of the DBE versus carbon number of all possible assignments in the filtered data ('filtered DBE plot'). The list of ions with possible matching formula are displayed and their profiles can be displayed as well. A file is also produced which is exported to MATLAB and contains the formula predictions of each ion, and DBE value of each candidate for further interrogation by the user. If more than one formula can be assigned to a peak this is also shown. We include code which can match assigned ions to those in the LIPIDMAPS. Another feature in the SIMS-MFP toolkit is a 'mass separator' button which is accessed in the 'mode' drop-down menu (**Supplementary Figure 1a**). This generates a separated spectrum containing all peaks that could not be matched to the preset criteria. A list of non-included ions is generated which can be put back into the formula finder if the user wishes to further interrogate separated species which may be of use when analyzing sample and substrate peaks, for example. The user can perform MVA on filtered datasets by accessing the MVA tab in the GUI (**Supplementary Figure 1b**). We show an example after running non-negative matrix factorization (NMF) on the filtered depth profile data. The number of iterations and endmembers can be chosen and are displayed in the lower portion of the GUI which shows loadings and profiles of each endmember (**Supplementary Figure 1d**). In each case the plots can be exported as png. or svg. files. SIMS-MFP can incorporate datasets from multiple samples, allowing other MVA techniques to be performed such as principal component analysis or K-means clustering. Filtered data can be exported to simsMVA[5] for further interrogation by MVA techniques.

Supplementary Figures

(a) Al clusters

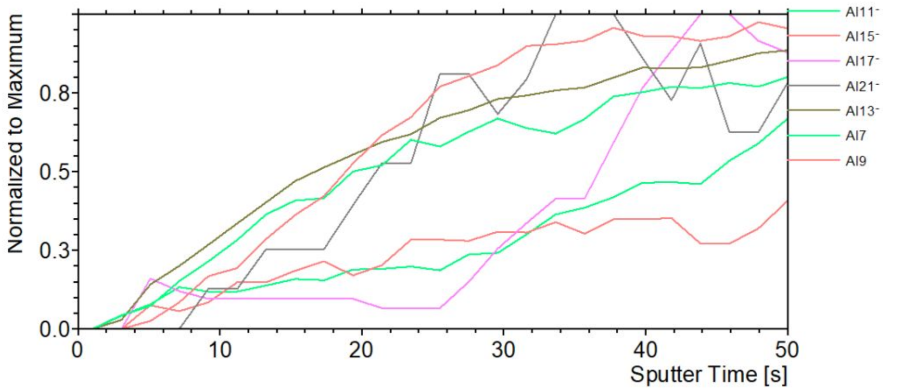

(b) Al oxides and hydroxides

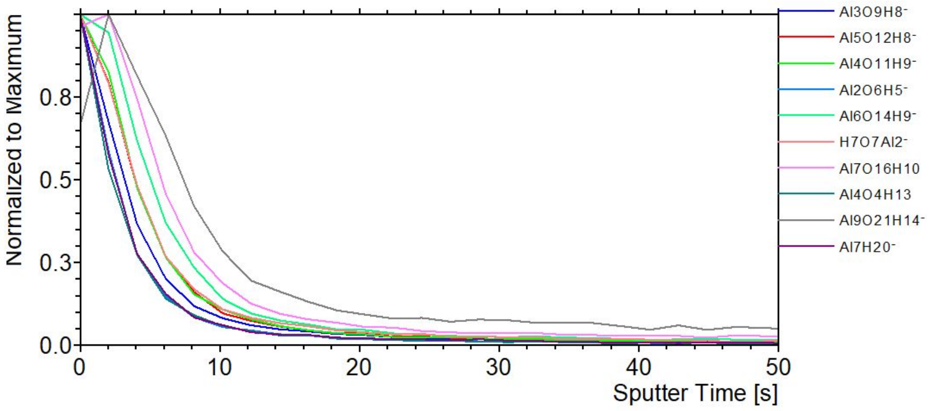

(c) Al oxides and hydroxides and intense organics ions

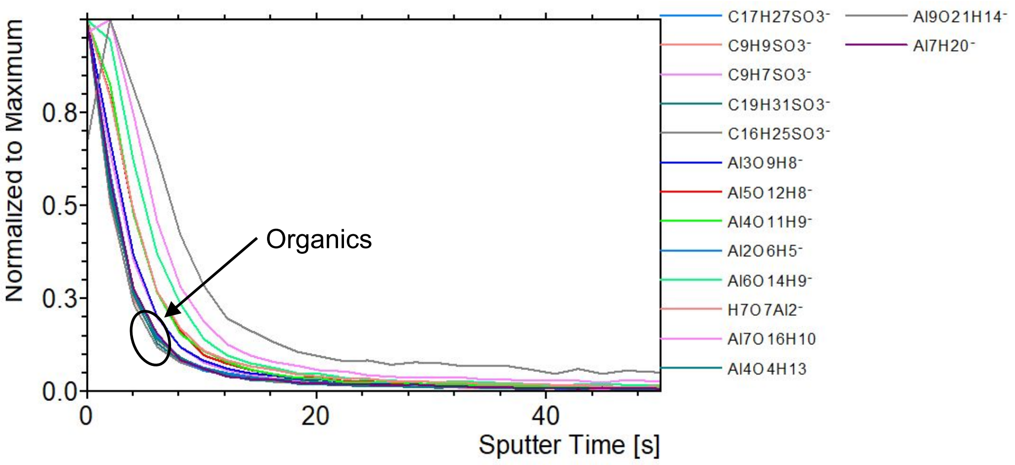

(d) Species in LIPIDMAPS

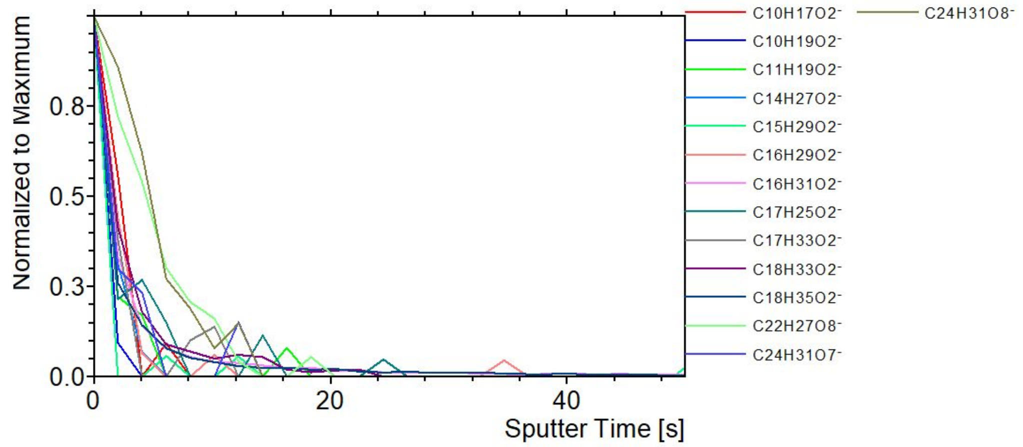

Supplementary Figure 2: 3D OrbiSIMS depth profiles of the organic and inorganic species on the dull side of the aluminum foil.

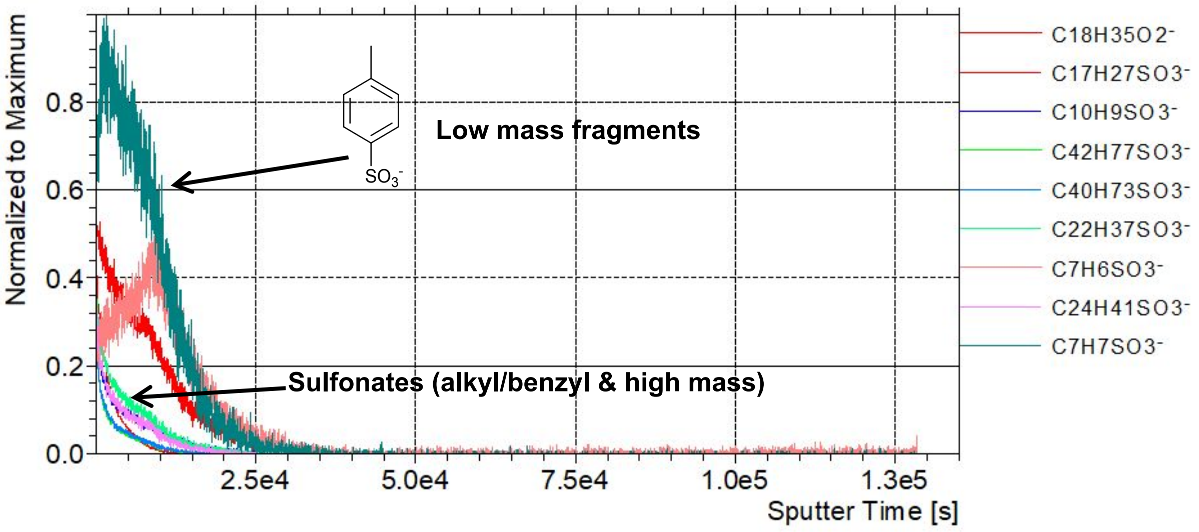

Supplementary Figure 3. Application of MFP to identify sulfated species (C & H = any, O = 3, S = 1) in engine deposits. Depth profiles of the 10 most intense species found in the search are shown.

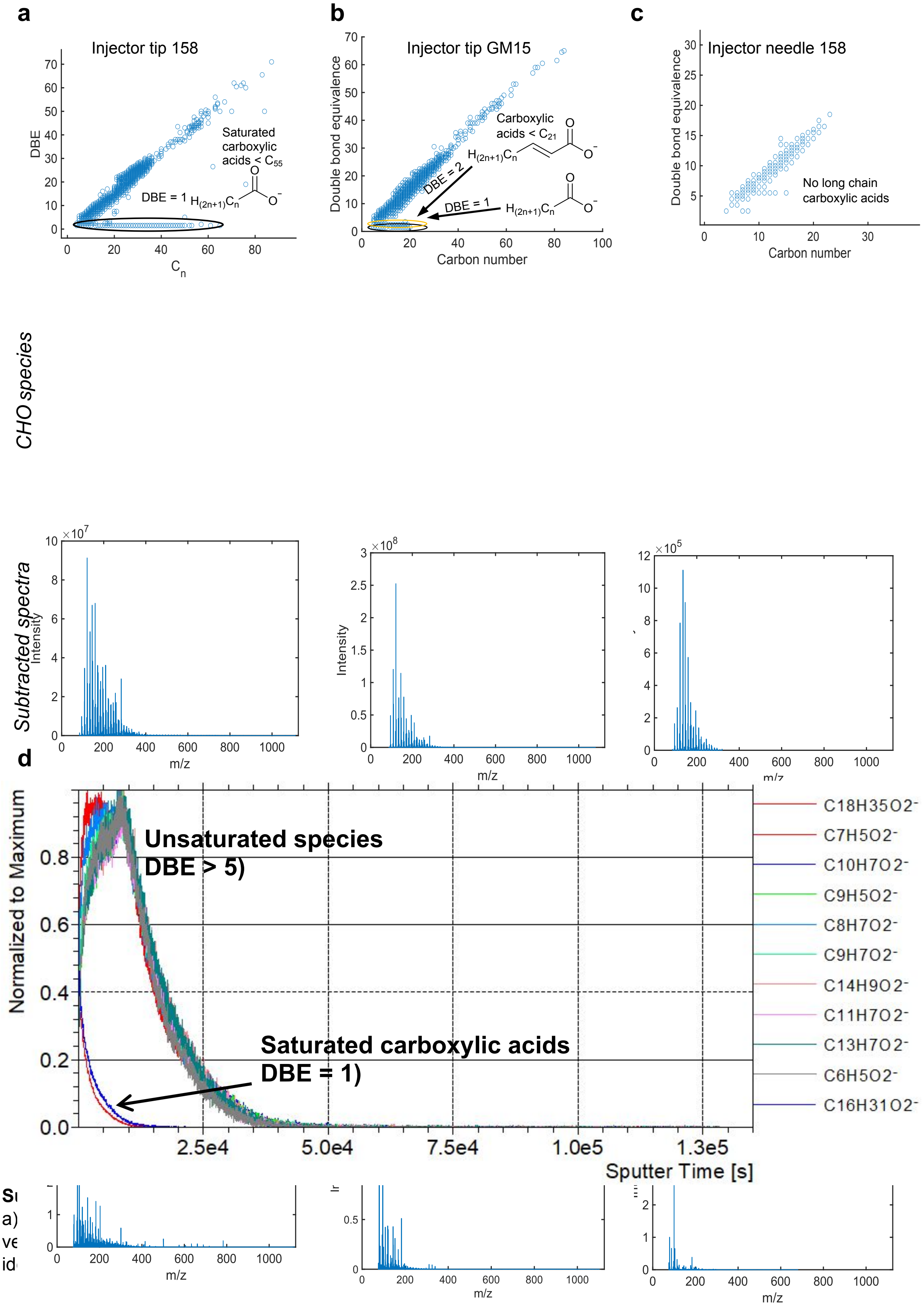
a
Injector tip 158
DBE
Cn
Saturated carboxylic acids < C55
DBE = 1
b
Injector tip GM15
Double bond equivalence
Carbon number
Carboxylic acids < C21
DBE = 2
DBE = 1
c
Injector needle 158
Double bond equivalence
Carbon number
No long chain carboxylic acids
CHO species
Subtracted spectra
Intensity
m/z
d
Normalized to Maximum
Unsaturated species DBE > 5)
Saturated carboxylic acids DBE = 1)
Sputter Time [s]
C18H35O2-
C7H5O2-
C10H7O2-
C9H5O2-
C8H7O2-
C9H7O2-
C14H9O2-
C11H7O2-
C13H7O2-
C6H5O2-
C16H31O2-

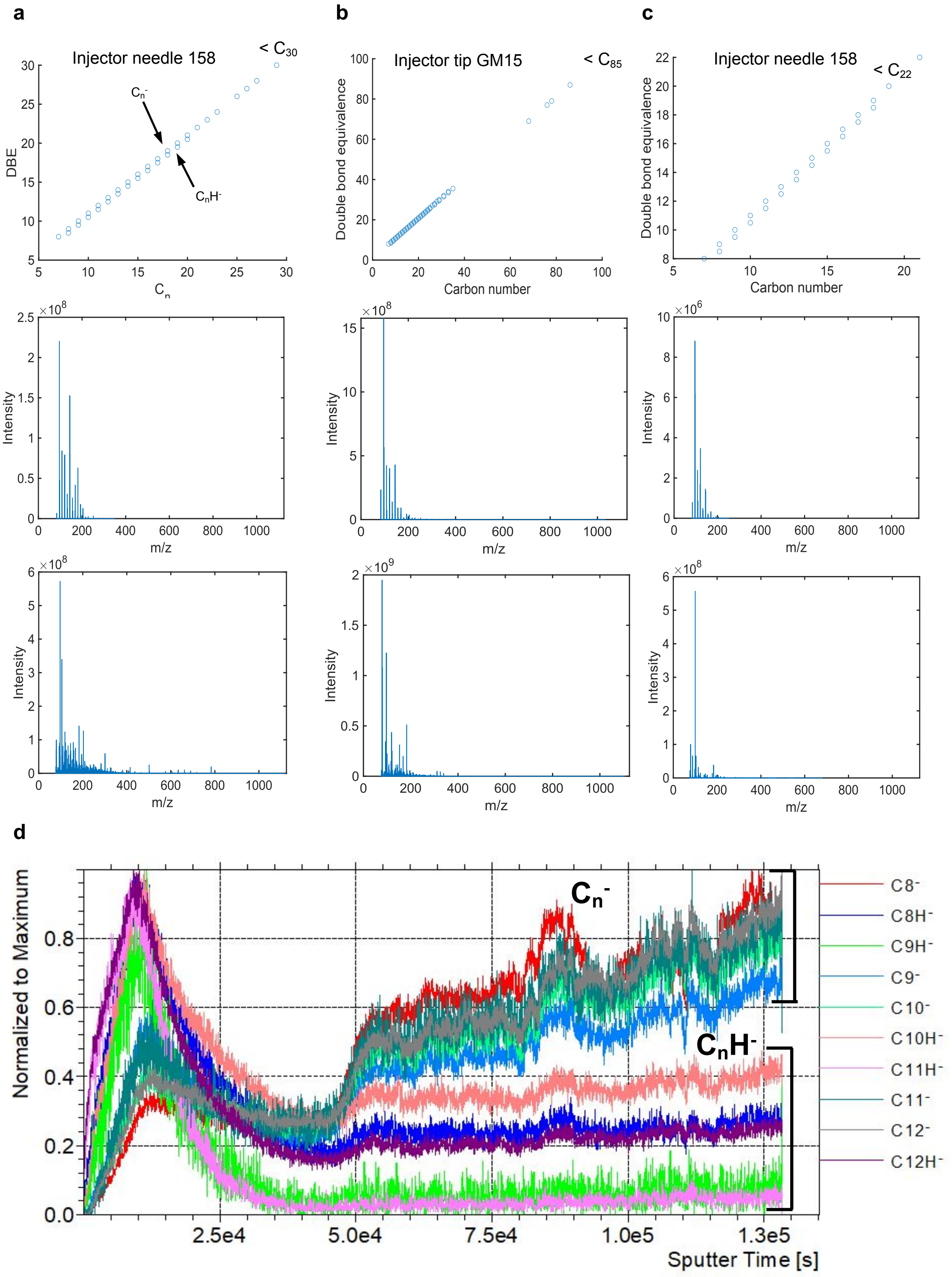
a
b
c
Injector needle 158
< C30
Cn-
CnH-
DBE
Cn
Injector tip GM15
< C85
Double bond equivalence
Carbon number
Injector needle 158
< C22
Double bond equivalence
Carbon number
Intensity
m/z
d
Normalized to Maximum
Sputter Time [s]
Cn-
CnH-
C8-
C8H-
C9H-
C9-
C10-
C10H-
C11H-
C11-
C12-
C12H-

e

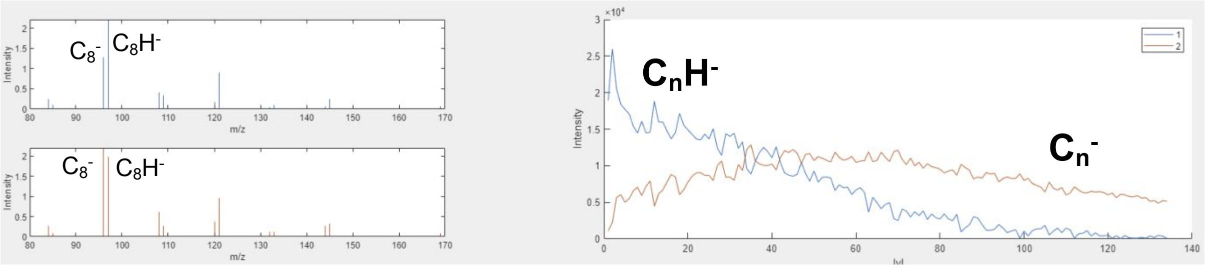

Supplementary Figure 5. Application of the SIMS-MFP software to identify carbonaceous ions (C = any, H ≤ 1) in engine deposits. a) injector tip 1, b) injector tip deposit 2 and c) injector needle 1. In each case the double bond equivalence (DBE) versus carbon number of each ion identified is displayed above, the middle plot shows the spectra of species identified and below are the subtracted spectra of species not found in the search. d) Depth profiles of intense ions in the filtered data set. e) NMF outputs on carbonaceous ions from injector needle 1.

a) 3 endmembers (200 iterations)

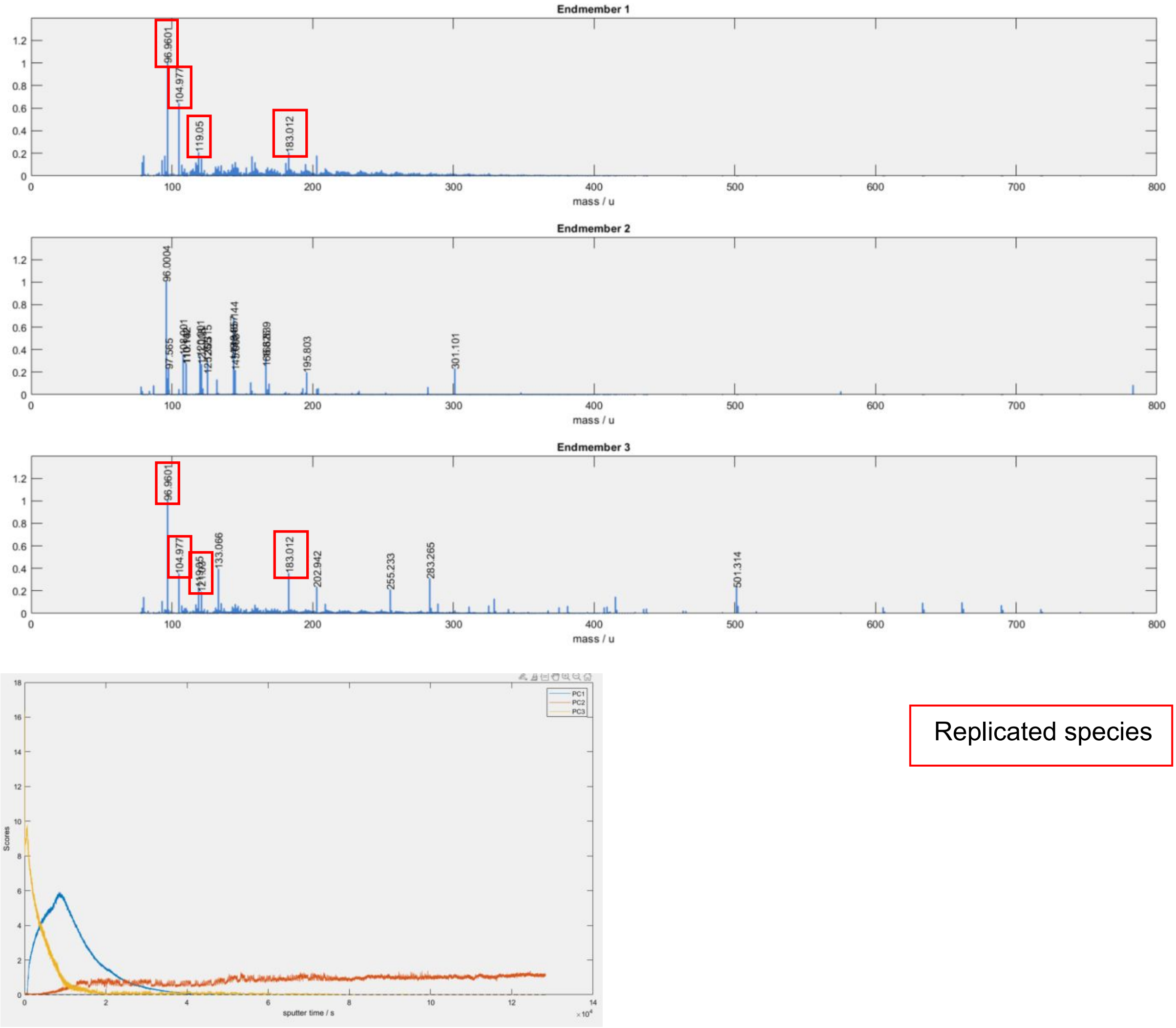

b) 4 endmembers (200 iterations)

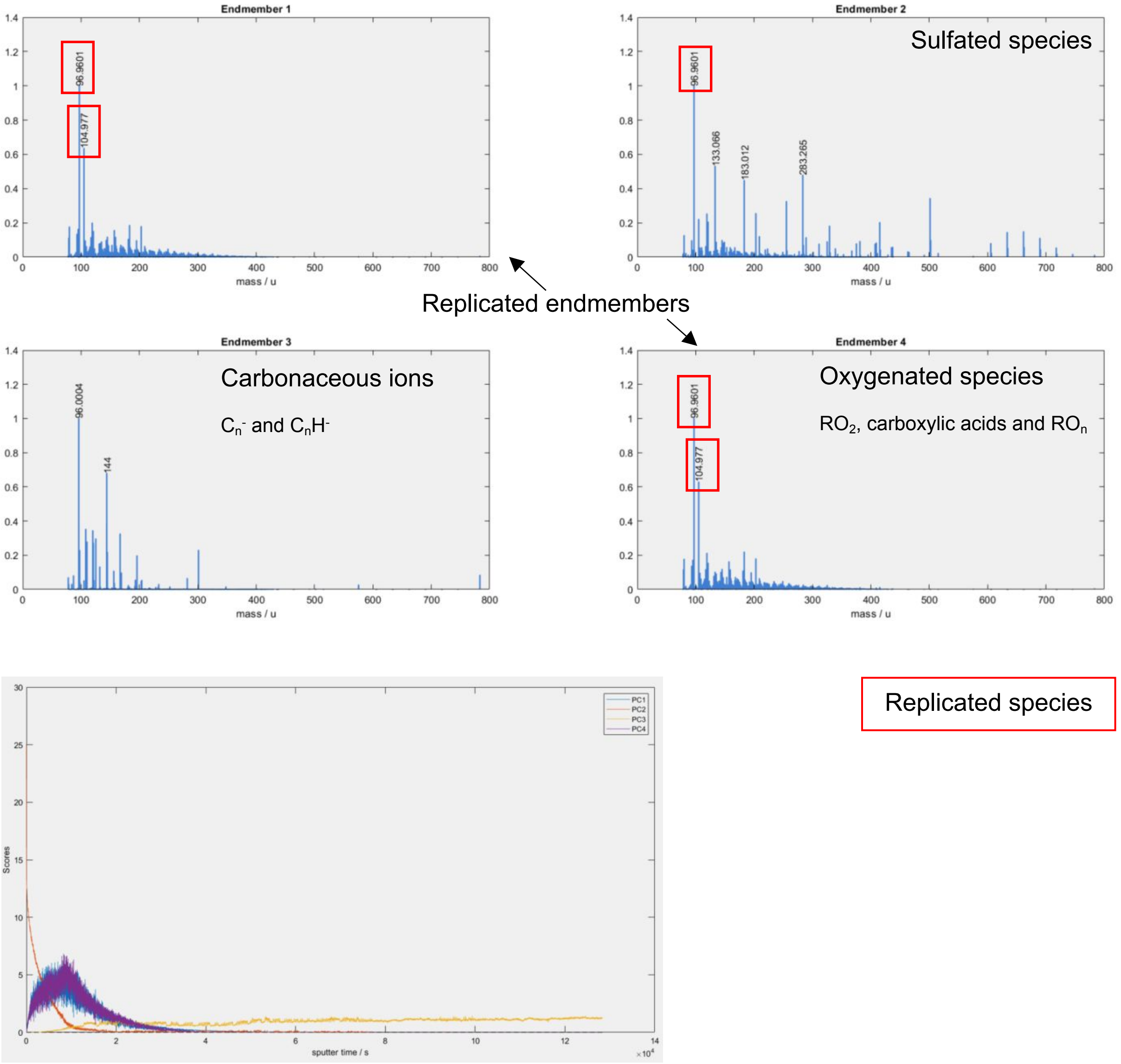

Supplementary Figure 6. NMF analysis on the raw 3D OrbiSIMS spectra with different conditions, a) with 3 endmembers, b) with 4 endmembers. In each case there was overfitting of data, loadings grouped species together with different elemental compositions and depth profiling behaviors.

Supplementary Table 1: Output data file from SIMS-MFP of molecular formula which fit the constraints of from the searches of the aluminum foil. a) Al search showing species containing at least one Al atom. b) Categories of organic species by compound class found by using MFP. i. sulfonated species on the dull side, ii. sulfonated species on the shiny side, iii. hydrocarbons, iv. ions with matching formulas in the LIPIDMAPS database. The full table of all possibilities of organic assignments can be found in the data repository.

a)

Mass	Deviation (ppm)	DBE	Area (AU)	H	O	Al
81.9528	-2.36599	0.5	29202.37	1	0	3
93.9851	-1.31829	-0.5	6304.99	3	4	1
94.9929	-1.56748	-1	215412.1	4	4	1
120.9667	-0.52824	-0.5	50637.13	3	4	2
135.0452	-2.06523	-5	3058.09	12	6	1
138.9772	-0.92749	-1.5	1581.66	5	5	2
153.9643	-0.77226	-1	8159.43	4	6	2
154.9722	-0.28328	-1.5	1201790	5	6	2
157.9997	2.285453	-64	4875.14	130	0	1
161.8897	-0.54914	1	1529.14	0	0	6
164.9508	-1.47862	-1	3050.22	4	5	3
169.9592	-0.78784	-1	4463.18	4	7	2
172.9827	-0.62954	-2.5	896617.4	7	7	2
185.0078	-1.58858	-5.5	668558.8	13	4	4
186.0155	-2.25196	-6	6841.45	14	4	4
188.8712	-0.68248	1	17995.48	0	0	7
188.9395	-2.34942	-2	1539.45	6	3	5
196.9406	-1.39077	-1	7909.87	4	7	3
198.9563	-1.12537	-2	28016.37	6	7	3
203.0869	-1.84108	-9.5	1875.03	21	8	2
207.0121	-0.38114	-8	179955.3	18	0	7
209.0078	1.153547	-65.5	2076.58	133	3	1
209.0277	-0.61666	-9	365964.6	20	0	7
210.0354	-1.20884	-9.5	10927.99	21	0	7
211.0191	-2.31685	-5.5	3120.67	13	9	2
211.0235	1.379469	-66.5	6722.66	135	3	1
211.0434	-0.37386	-10	156752	22	0	7
212.051	-1.43314	-10.5	4717.13	23	0	7
212.9392	-0.98103	-62	6239.8	126	2	2
213.0349	-1.59082	-6.5	2032.9	15	9	2
213.0392	1.601116	-67.5	3124.41	137	3	1
213.0588	-1.5437	-11	2889.3	24	0	7
213.9435	-0.53238	-1.5	10948.61	5	8	3
214.9513	-0.64619	-2	12324.95	6	8	3
219.8909	-0.97275	1	2648.68	0	7	4
222.9145	-0.39881	-0.5	13956.12	3	7	4
224.9984	-2.01734	-4.5	3661.26	11	10	2
227.014	-2.21968	-5.5	1604.1	13	10	2
229.0296	-2.41846	-6.5	2111.22	15	10	2

230.9498	-0.75298	-63	3515.04	128	3	2
230.9701	-0.62302	-6.5	15964.91	15	0	8
231.0453	-2.18095	-7.5	3092.73	17	10	2
231.9542	0.090966	-2.5	1910.03	7	9	3
232.897	-0.03821	-61	1674.67	124	0	4
232.9619	-0.446	-3	2453274	8	9	3
235.8859	-0.54645	1	2038.91	0	8	4
240.0779	-2.28634	-11.5	12662.63	25	5	5
242.8341	-1.27206	1	16137.36	0	0	9
244.0408	-1.36821	-10.5	1646.89	23	2	7
244.9291	-0.5671	-62	3319.85	126	4	2
247.9488	-1.18532	-2.5	4929	7	10	3
248.9604	-0.55792	-64	6450.88	130	4	2
249.0224	0.767401	-69	2295.21	140	0	4
255.0036	-1.05449	-6	2315.91	14	10	3
256.9197	-1.43586	-1.5	6397.06	5	9	4
256.9439	-0.22923	-6	1886	14	0	9
258.9354	-1.23158	-2.5	14957.81	7	9	4
265.1478	1.475029	-77	6666.44	156	0	4
266.9023	-0.70775	-62	6290.46	126	2	4
266.9406	1.240352	-62.5	2124.37	127	7	1
268.1092	-0.05184	-73.5	2645.02	149	4	2
269.9835	-1.90345	-7.5	2998	17	4	7
272.9387	-0.63714	-6	11529.97	14	1	9
273.9227	-0.39756	-2	6549.23	6	10	4
274.9306	-0.1233	-2.5	293296.2	7	10	4
276.0415	-0.10469	-69.5	2922.1	141	5	2
276.946	-1.02511	-3.5	6642.39	9	10	4
277.9468	-1.30924	-6	1631.78	14	3	8
281.0767	-1.18793	-11	3047.29	24	11	3
282.8972	-0.72076	-62	4146.27	126	3	4
282.9355	1.117217	-62.5	5475.78	127	8	1
283.9424	-2.14444	-63	1676.86	128	8	1
284.9396	-2.34751	-4.5	1825.86	11	7	6
284.9777	-1.2243	-5	3069.42	12	12	3
291.0496	2.443232	-12.5	6319.25	27	3	8
292.9412	0.003755	-3.5	1304009	9	11	4
296.7971	-1.31032	1	9700.74	0	0	11
298.8881	-2.00376	-1	1746.54	4	10	5
298.9123	-0.9665	-5.5	1528.59	13	1	10
300.9041	-0.82717	-2	8798.71	6	10	5
300.946	0.83437	-63.5	21292.28	129	9	1
301.1269	-0.79335	-17.5	19725.67	37	3	8
302.9618	1.323931	-64.5	5982.54	131	9	1
310.9071	0.196522	-6	23629.31	14	0	11
312.9399	-0.95513	-64	4938.33	130	8	2

314.1677	1.610926	-19	3247.27	40	7	6
314.9075	-0.01238	-5.5	4991.72	13	2	10
316.8989	-1.14831	-2	86121.06	6	11	5
316.9409	0.744934	-63.5	10638.62	129	10	1
318.9146	-0.98428	-3	49841.9	8	11	5
318.9773	2.244992	-8	18616.73	18	7	7
321.153	-1.10197	-19.5	2471.43	41	4	8
323.1684	-1.86868	-20.5	29233.22	43	4	8
323.7786	-1.32467	1	2130.99	0	0	12
324.9041	0.880568	-62	3430.59	126	9	2
326.9199	1.333968	-63	5664.87	128	9	2
327.1798	-0.7149	-79	25940.16	160	7	2
329.0854	-0.81711	-15.5	1705.15	33	5	8
331.8862	-0.46371	-1.5	2805.83	5	12	5
332.9358	0.664092	-63.5	1850.93	129	11	1
333.9018	-0.61066	-2.5	1722.55	7	12	5
334.8652	0.286981	-4.5	2857.95	11	0	12
334.9098	-0.08629	-3	1340626	8	12	5
338.1877	2.2801	-22.5	2243.7	47	3	9
340.203	1.237792	-23.5	91269.14	49	3	9
342.9146	0.644767	-63	49945.41	128	10	2
344.9303	0.785957	-64	5542.95	130	10	2
344.9567	-1.57672	-5.5	1709.57	13	14	4
346.898	1.920165	-5.5	1713.31	13	4	10
348.8861	0.949021	-65	3429.6	132	0	8
349.8966	-0.91141	-2.5	27254.5	7	13	5
350.7602	-1.05171	1	51917.75	0	0	13
352.8757	0.088133	-5.5	35307.89	13	1	12
352.9201	-0.83277	-4	40186.92	10	13	5
353.2155	0.229605	-83.5	2991.85	169	3	5
358.9097	1.131484	-63	7517.81	128	11	2
360.9253	0.986632	-64	2416.75	130	11	2
361.0065	-1.06341	-12.5	4500.14	27	4	10
364.854	2.264198	0.5	2284.31	1	16	4
367.9073	-0.49985	-3.5	5101.49	9	14	5
376.878	-1.21763	-2.5	62255.23	7	13	6
376.9201	0.639659	-64	2396.32	130	12	2
376.977	-2.41102	-8	4376.94	18	14	5
378.8938	-0.81527	-3.5	6509.01	9	13	6
380.8102	-0.28597	1	13134.39	0	12	7
390.9117	-1.91577	-8.5	3025.44	19	3	12
390.9926	2.483685	-70.5	37688.26	143	7	5
391.9959	0.168624	-10	2669.14	22	13	6
392.9421	0.282739	-5.5	1797.47	13	17	4
393.8808	-1.01274	-3	5938.65	8	14	6
394.8888	-0.56699	-3.5	1087778	9	14	6

395.8929	-1.57593	-67	1890.61	136	1	9
397.2269	-0.03499	-25.5	3301.21	53	8	8
399.2426	0.090421	-26.5	5782.89	55	8	8
400.8368	0.688809	-1	2540.34	4	13	7
402.8938	0.56119	-63.5	46224.41	129	12	3
404.7232	-1.10915	1	3493.63	0	0	15
404.7783	-1.57839	-56.5	3641.25	115	13	3
404.9099	1.669757	-64.5	2043.09	131	12	3
410.9107	-1.82253	-69	4008.89	140	0	10
412.8549	0.08744	-6	23628.63	14	3	13
412.8994	-0.4575	-4.5	33010.23	11	15	6
418.8888	0.74268	-63.5	3135.5	129	13	3
420.1835	-1.42533	-21.5	1561.28	45	15	5
420.9254	2.35458	-8	2205.53	18	10	9
420.9734	1.534779	-67.5	2314.24	137	16	1
422.878	-0.91965	-4	2567.98	10	14	7
434.8689	-1.41169	-67.5	2382.1	137	0	11
436.7681	-1.53147	-56.5	2913.45	115	15	3
436.8571	-1.26792	-3	24950.08	8	15	7
436.8993	0.563288	-64.5	3586.29	131	14	3
438.8729	-0.92031	-4	24098.95	10	15	7
438.936	2.337704	-9	2606.05	20	11	9
444.8628	1.339965	-63	3079.37	128	13	4
446.8784	1.222034	-64	2728.54	130	13	4
450.8465	-2.18233	-61	7394.06	124	17	2
452.8795	-1.27826	-68.5	23665.13	139	1	11
454.868	-0.48124	-4	733576.5	10	16	7
456.8836	-0.58855	-5	5443.52	12	16	7
458.6863	-0.93506	1	1977.8	0	0	17
462.8731	0.715315	-64	29426.72	130	14	4
464.8464	-1.33141	-3.5	2200.82	9	15	8
464.8885	0.17445	-65	1585.85	132	14	4
472.8341	0.086923	-6.5	5342.63	15	5	14
472.8784	-0.81184	-5	314998.5	12	17	7
476.8374	-1.56007	-67	3615.09	136	1	12
478.8255	-1.63713	-2.5	12168.34	7	16	8
478.8682	1.077751	-64	2992.68	130	15	4
480.8415	-0.90238	-3.5	16318.35	9	16	8
480.8835	0.345406	-65	16139.15	132	15	4
486.8231	-1.6205	-64.5	2477.15	131	7	9
490.8446	-0.04869	-7.5	4459.35	17	6	14
494.8477	-2.0388	-68	1556.51	138	2	12
494.8784	-2.31147	-70.5	1621.41	143	0	13
495.8287	-0.65325	-3	12089.7	8	17	8
496.8366	-0.50097	-3.5	341243.3	9	17	8
496.8785	0.505355	-65	2344.72	132	16	4

498.8523	-0.39872	-4.5	4357.32	11	17	8
503.1381	1.343768	-24.5	3592.69	51	8	12
504.8417	0.596425	-63.5	19519.03	129	15	5
511.8235	-0.85752	-3	8127.86	8	18	8
512.6489	-1.77295	1	1867.35	0	0	19
512.8584	-1.70398	-69	26787.39	140	3	12
514.8026	-0.17269	-6	6929.98	14	6	15
514.8346	2.09019	-8.5	41063.77	19	4	16
514.847	-0.80393	-4.5	143334.2	11	18	8
519.1491	-1.44255	-81.5	7266.41	165	17	3
520.1519	2.251463	-84	2472.89	170	10	7
520.8364	0.165311	-63.5	2349.5	129	16	5
522.852	0.069044	-64.5	7139.5	131	16	5
532.8451	1.897553	-9.5	4832.82	21	5	16
532.8575	-0.89874	-5.5	49580.92	13	19	8
538.8044	-2.00239	-3	4106.47	8	18	9
540.8205	-1.16286	-4	4869.56	10	18	9
550.868	-0.98735	-6.5	1618.11	15	20	8
554.8116	1.155529	-62	3299.58	126	20	4
554.827	-1.62915	-68.5	16224.14	139	4	13
556.8035	2.417555	-8	7175.95	18	5	17
556.8155	-0.9768	-4	67832.97	10	19	9
558.8311	-1.06275	-5	1945.4	12	19	9
564.821	0.718989	-64	2596.35	130	17	6
566.6124	-1.86	-49.5	1597.33	101	24	3
566.6124	-0.86285	1	1597.33	0	0	21
570.7927	1.254573	-66	2023.35	134	7	12
570.8046	-2.23176	-62	52757.65	126	21	4
574.8141	2.402696	-9	3334.66	20	6	17
574.8262	-0.71134	-5	152035.5	12	20	9
576.8307	-2.23445	-71	2347.48	144	0	16
576.8418	-0.79554	-6	1859.49	14	20	9
582.8313	0.242094	-65	10533.82	132	18	6
592.8369	-0.46202	-6	473484.2	14	21	9
594.8411	-2.44418	-72	11451.41	146	1	16
598.11	0.578656	-83	7906.48	168	15	7
598.7719	1.863985	-7.5	8138.63	17	6	18
598.7835	-1.96047	-3.5	2486.63	9	20	10
600.7994	-1.53778	-4.5	1715.84	11	20	10
600.8419	0.293089	-66	24342.64	134	19	6
602.8042	-2.49483	-70.5	8650.9	143	0	17
614.8061	-1.62474	-69	23534.69	140	6	14
616.7825	1.866302	-8.5	7165.81	19	7	18
616.7948	-0.71158	-4.5	94714.97	11	21	10
618.8101	-1.27486	-5.5	1972.34	13	21	10
624.7995	-0.46239	-64.5	2272.9	131	19	7

630.7837	-2.17015	-62.5	56721.14	127	23	5
631.7817	-0.99544	-4	1743.03	10	22	10
634.8055	-0.47873	-5.5	121932.7	13	22	10
636.81	-1.85911	-71.5	2847.78	145	2	17
640.7522	-1.72282	-3	3742.27	8	21	11
642.8105	0.227283	-65.5	4737.88	133	20	7
644.7726	2.048944	-59.5	4441.15	121	31	1
648.7942	-2.2101	-63.5	7499.56	129	24	5
648.7942	-1.33925	-13	7499.56	28	0	23
649.7926	-0.4523	-5	4745.4	12	23	10
652.7714	-0.37364	-8	2414.52	18	11	17
652.8161	-0.41191	-6.5	57372.26	15	23	10
656.7729	1.204527	-7.5	1733.43	17	13	16
658.7513	1.147779	-58.5	4929.29	119	32	1
658.7513	2.005465	-8	4929.29	18	8	19
658.7634	-0.71179	-4	82697.02	10	22	11
660.7787	-1.23929	-5	2804.9	12	22	11
663.1981	2.27851	-89	2994.34	180	20	6
666.7684	-0.02835	-64	2049.16	130	20	8
672.7525	-1.78208	-62	2325.56	126	24	6
672.7525	-0.94225	-11.5	2325.56	25	0	24
674.7855	-1.17652	-69.5	7444.77	141	8	15
676.7739	-0.78889	-5	158328.5	12	23	11
678.7784	2.417732	-60.5	1787.86	123	33	1
678.7784	-2.083	-71	1787.86	144	3	18
684.779	0.023511	-65	3938.77	132	21	8
690.763	-1.82971	-63	25286.2	128	25	6
690.763	-1.01178	-12.5	25286.2	27	1	24
691.2296	-0.74346	-37.5	2550.31	77	8	18
692.7955	-1.96147	-70.5	1554.2	143	9	15
694.7845	-0.71806	-6	199984	14	24	11
696.7887	1.974921	-61.5	5090.96	125	34	1
696.7887	-2.40948	-72	5090.96	146	4	18
702.7896	0.07271	-66	6189.19	134	22	8
708.7735	-1.87493	-64	2127.22	130	26	6
708.7735	-1.07778	-13.5	2127.22	29	2	24
712.795	-0.79111	-7	73770.21	16	25	11
716.7534	-2.12611	-69	2005.94	140	9	16
718.7426	-0.64543	-4.5	16000.37	11	24	12
722.7618	1.053045	-61	1607.32	124	34	2
722.7618	1.834771	-10.5	1607.32	23	10	20
732.7311	-2.44823	-62.5	8359.36	127	26	7
732.7311	2.492189	-1.5	8359.36	5	32	8
732.7311	-1.67715	-12	8359.36	26	2	25
734.7645	-1.34587	-70	5654.86	142	10	16
736.741	0.944838	-60	3632.61	122	35	2

736.741	1.711731	-9.5	3632.61	21	11	20
736.7532	-0.58215	-5.5	48166.19	13	25	12
750.7421	-1.81007	-63.5	21860.15	129	27	7
750.7421	-1.05749	-13	21860.15	28	3	25
752.7744	-2.19707	-71	2090.45	144	11	16
754.7515	0.83617	-61	1615.77	124	36	2
754.7515	1.584763	-10.5	1615.77	23	12	20
754.7636	-0.78687	-6.5	48259.7	15	26	12
760.7093	1.223991	-67	5436.34	136	12	16
764.7312	2.002145	-60.5	1520.84	123	35	3
764.7312	-1.99272	-71	1520.84	144	5	20
768.7524	-2.11238	-64.5	9037.25	131	28	7
768.7524	-1.37742	-14	9037.25	30	4	25
772.7741	-0.85264	-7.5	16513.84	17	27	12
778.7214	-1.10296	-5	43712.09	12	26	13
782.9308	-1.77397	-74.5	2395.38	151	31	5
782.9308	-1.05233	-24	2395.38	50	7	23
792.7105	-2.00438	-63	7411.53	128	28	8
792.7105	-1.29164	-12.5	7411.53	27	4	26
796.7319	-1.15961	-6	166162.3	14	27	13
798.736	1.06556	-61.5	3684.13	125	37	3
798.736	1.772929	-11	3684.13	24	13	21
804.7375	0.156697	-66	3545.97	134	25	10
804.7375	0.85879	-15.5	3545.97	33	1	28
805.6601	0.572326	-57	6704.97	116	38	3
805.6601	1.273616	-6.5	6704.97	15	14	21
806.6627	-1.08335	-70	1712.3	142	1	24
810.7213	-1.66999	-64	8319.18	130	29	8
810.7213	-0.97308	-13.5	8319.18	29	5	26
814.7425	-1.09102	-7	85343.27	16	28	13
820.6886	1.262478	-67.5	1622.74	137	14	17
822.7475	-0.53346	-67	3739.08	136	26	10
822.7475	0.153267	-16.5	3739.08	35	2	28
828.7317	-1.8328	-65	2281.57	132	30	8
828.7317	-1.15103	-14.5	2281.57	31	6	26
832.753	-1.14548	-8	47756.77	18	29	13
833.6908	1.152827	-68.5	19895.85	139	13	18
834.6943	-0.37008	-58.5	9660.89	119	43	1
834.6943	0.306819	-8	9660.89	18	19	19
836.7122	2.319919	-59.5	2451.36	121	43	1
836.7122	-1.33128	-70	2451.36	142	13	18
838.7012	-0.30273	-5.5	18205.62	13	28	14
852.6897	-1.85753	-63.5	12964.92	129	30	9
852.6897	2.387861	-2.5	12964.92	7	36	10
852.6897	-1.19492	-13	12964.92	28	6	27
854.7226	2.077988	-60.5	2193.17	123	44	1

854.7226	-1.49627	-71	2193.17	144	14	18
856.6984	-0.34306	-61	1835.08	124	39	4
856.6984	0.316448	-10.5	1835.08	23	15	22
856.7115	-0.60569	-6.5	29082.96	15	29	14
861.7225	-1.65819	-67.5	16151.86	137	25	12
861.7225	-1.00253	-17	16151.86	36	1	30
862.7265	-1.89388	-7	10341.58	16	31	13
862.7265	1.960185	-70	10341.58	142	18	16
870.6999	-2.23831	-64.5	10686.7	131	31	9
870.6999	1.919264	-3.5	10686.7	9	37	10
870.6999	-1.58941	-14	10686.7	30	7	27
874.7219	-0.78185	-7.5	21714.19	17	30	14
880.6688	-1.45787	-5	13205.71	12	29	15
880.6688	2.317676	-68	13205.71	138	16	18
888.7111	-1.47843	-65.5	2627.37	133	32	9
888.7111	-0.84268	-15	2627.37	32	8	27
889.7542	-0.22354	-5.5	3427.01	13	43	7
889.7542	0.07991	-79	3427.01	160	0	27
890.7576	-1.45819	-69	2136.35	140	30	10
890.7576	-0.8239	-18.5	2136.35	39	6	28
892.7327	-0.50284	-8.5	4653.6	19	31	14
898.6795	-1.27843	-6	46504.7	14	30	15
898.6795	2.421448	-69	46504.7	140	17	18
912.6681	1.359861	-3	4785.81	8	38	11
912.6681	-1.98747	-13.5	4785.81	29	8	28
916.6901	-1.21513	-7	92431.71	16	31	15
916.6901	2.412053	-70	92431.71	142	18	18
918.6945	1.04616	-62.5	1933.54	127	41	5
918.6945	-2.27921	-73	1933.54	148	11	22
918.6945	1.661164	-12	1933.54	26	17	23
930.6793	-1.87379	-65	4280.68	132	33	10
930.6793	2.015843	-4	4280.68	10	39	11
930.6793	-1.26671	-14.5	4280.68	31	9	28
934.7007	-1.15427	-8	54620.55	18	32	15
934.7007	2.403021	-71	54620.55	144	19	18
940.6472	-2.21007	-5.5	2528.65	13	31	16
940.6472	1.324728	-68.5	2528.65	139	18	19
952.7115	-0.88579	-9	13503.7	20	33	15
954.6363	0.849644	-2.5	1932.2	7	39	12
954.6363	-2.35052	-13	1932.2	28	9	29
958.6593	-0.56735	-6.5	11926.17	15	32	16
972.6473	-2.44065	-64.5	6835.33	131	34	11
972.6473	1.281144	-3.5	6835.33	9	40	12
972.6473	-1.85977	-14	6835.33	30	10	29
976.6697	-0.72583	-7.5	14488.9	17	33	16
982.6166	-1.33205	-5	2994.6	12	32	17

982.6166	2.051771	-68	2994.6	138	19	20
990.6581	-2.15907	-65.5	6748.9	133	35	11
990.6581	1.495069	-4.5	6748.9	11	41	12
990.6581	-1.58874	-15	6748.9	32	11	29
994.6799	-1.07964	-8.5	4559.81	19	34	16
994.6799	2.263145	-71.5	4559.81	145	21	19
1000.627	-1.77279	-6	20101.58	14	33	17
1000.627	1.550131	-69	20101.58	140	20	20
1008.668	0.908228	-5.5	1855.88	13	42	12
1008.668	-2.12051	-16	1855.88	34	12	29
1018.638	-1.3144	-7	56745.63	16	34	17
1018.638	1.949765	-70	56745.63	142	21	20
1032.627	-2.10037	-65	2121.67	132	36	12
1032.627	1.405253	-4	2121.67	10	42	13
1032.627	-1.55322	-14.5	2121.67	31	12	30
1036.648	-1.35427	-8	51168.79	18	35	17
1036.648	1.853188	-71	51168.79	144	22	20
1038.573	0.241774	-1.5	1823.71	5	41	14
1040.607	1.240719	-59.5	1916.97	121	49	5
1040.607	-1.69507	-70	1916.97	142	19	22
1040.607	1.783673	-9	1916.97	20	25	23
1054.658	-1.86686	-9	10774.39	20	36	17
1054.658	1.285821	-72	10774.39	146	23	20
1056.584	0.744002	-2.5	3066.42	7	42	14
1056.584	-2.14739	-13	3066.42	28	12	31
1060.606	-1.85639	-6.5	4709.2	15	35	18
1060.606	1.278611	-69.5	4709.2	141	22	21
1074.597	-0.92956	-64.5	4204.08	131	37	13
1074.597	2.439154	-3.5	4204.08	9	43	14
1074.597	-0.40378	-14	4204.08	30	13	31
1078.618	0.061282	-7.5	16474.9	17	36	18
1080.545	0.001018	-62	1775.88	126	36	14
1080.545	0.523903	-11.5	1775.88	25	12	32
1084.566	-0.64441	-55.5	80878.64	113	59	1
1084.566	-0.12346	-5	80878.64	12	35	19
1086.571	-0.10483	-71	1535.24	144	15	26
1092.607	-1.2483	-65.5	1648.47	133	38	13
1092.607	2.064883	-4.5	1648.47	11	44	14
1092.607	-0.73119	-15	1648.47	32	14	31
1092.607	2.311999	-78	1648.47	158	1	34
1102.575	-1.32771	-56.5	21976.82	115	60	1
1102.575	-0.81527	-6	21976.82	14	36	19
1102.575	2.200399	-69	21976.82	140	23	22
1120.586	-1.00742	-57.5	36511.15	117	61	1
1120.586	-0.50322	-7	36511.15	16	37	19
1120.586	2.463984	-70	36511.15	142	24	22

(b)

i. Sulfonated species 'dull side'

Total ion counts	65318098							
**Mass**	**Deviation (ppm)**	**DBE**	**Area (AU)**	**Area (normalised to total)**	**H**	**C**	**O**	**S**
79.9571	-3.313015634	1	1410502	0.021594359	0	0	3	1
80.9649	-3.580551103	0.5	60736.96	0.000929864	1	0	3	1
93.0012	-4.031114531	0.5	4341.78	6.64713E-05	5	2	2	1
94.9806	-2.525772569	0.5	1823.17	2.79122E-05	3	1	3	1
106.9809	0.561782839	1.5	4474.98	6.85106E-05	3	2	3	1
154.9807	-0.90269223	5.5	7529.78	0.000115279	3	6	3	1
155.9885	-1.057128094	5	21774.88	0.000333367	4	6	3	1
156.9964	-0.572624268	4.5	184933.2	0.002831271	5	6	3	1
170.0043	-0.087644834	5	457859.3	0.007009685	6	7	3	1
171.0121	-0.233316769	4.5	3294.94	5.04445E-05	7	7	3	1
180.9966	0.608299089	6.5	186107.8	0.002849253	5	8	3	1
182.0042	-0.631303714	6	3418.3	5.23331E-05	6	8	3	1
183.012	-0.764430163	5.5	16487489	0.252418393	7	8	3	1
185.0274	-2.107251026	4.5	10200.43	0.000156165	9	8	3	1
194.0041	-1.107707322	7	9953	0.000152377	6	9	3	1
195.0121	-0.204602647	6.5	1046453	0.016020872	7	9	3	1
196.0199	-0.331088724	6	216077.5	0.00330808	8	9	3	1
197.0277	-0.456280812	5.5	1179238	0.018053764	9	9	3	1
198.0354	-1.085158344	5	21783.01	0.000333491	10	9	3	1
199.0433	-0.702861647	4.5	5890.7	9.01848E-05	11	9	3	1
220.0198	-0.74947744	8	6971.02	0.000106724	8	11	3	1
221.0276	-0.859167981	7.5	56598.33	0.000866503	9	11	3	1
223.0433	-0.627232077	6.5	63655.04	0.000974539	11	11	3	1
225.0591	0.044877103	5.5	161277.1	0.002469103	13	11	3	1
227.0746	-0.616096709	4.5	5045.03	7.72379E-05	15	11	3	1
233.0276	-0.814924263	8.5	8708.8	0.000133329	9	12	3	1
235.0432	-1.020662394	7.5	12561.1	0.000192307	11	12	3	1
237.059	-0.379230343	6.5	19805.2	0.000303212	13	12	3	1
239.0747	-0.166893416	5.5	169418.7	0.002593749	15	12	3	1
241.0902	-0.78767138	4.5	2222.52	3.40261E-05	17	12	3	1
247.0432	-0.971084276	8.5	3510.43	5.37436E-05	11	13	3	1
249.0589	-0.762469662	7.5	2523.48	3.86337E-05	13	13	3	1
250.9813	1.833207667	13.5	2284.82	3.49799E-05	3	14	3	1
251.0745	-0.955492377	6.5	6193.21	9.48161E-05	15	13	3	1
253.0902	-0.750324815	5.5	63732.73	0.000975729	17	13	3	1
255.106	-0.156405548	4.5	4814.29	7.37053E-05	19	13	3	1
265.0902	-0.716359428	6.5	3934.13	6.02303E-05	17	14	3	1
267.1058	-0.898145172	5.5	35113.34	0.000537574	19	14	3	1
269.1215	-0.705628744	4.5	4592.12	7.03039E-05	21	14	3	1
273.0016	0.091940861	15.5	1713.35	2.62309E-05	5	17	2	1
279.1057	-1.217816713	6.5	2035.94	3.11696E-05	19	15	3	1

281.0281	1.103449592	12.5	11803.52	0.000180708	9	16	3	1
281.1215	-0.675508176	5.5	26750	0.000409534	21	15	3	1
283.1371	-0.84729199	4.5	9159.3	0.000140226	23	15	3	1
293.1216	-0.306698559	6.5	2488.22	3.80939E-05	21	16	3	1
295.137	-1.151667221	5.5	30857.55	0.00047242	23	16	3	1
297.1528	-0.639064746	4.5	492311.7	0.007537141	25	16	3	1
307.1371	-0.781083798	6.5	1980.87	3.03265E-05	23	17	3	1
309.1528	-0.614258979	5.5	41137.21	0.000629798	25	17	3	1
311.1686	-0.128226289	4.5	1402522	0.021472181	27	17	3	1
335.1687	0.179312719	6.5	1569.71	2.40318E-05	27	19	3	1
337.1842	-0.266619777	5.5	16850.5	0.000257976	29	19	3	1
339.1998	-0.412441111	4.5	630590.6	0.009654148	31	19	3	1

ii. Sulfonates on 'shiny' side

Total ion counts	13252804							
**Mass**	**Deviation (ppm)**	**DBE**	**Area (AU)**	**Area (normalised to total)**	**H**	**C**	**O**	**S**
79.957	-4.56368	1	405941.7	0.03063063	0	0	3	1
80.9648	-4.81565	0.5	8469.62	0.000639081	1	0	3	1
155.9885	-1.05713	5	1193.58	9.00625E-05	4	6	3	1
156.9963	-1.20958	4.5	9213.94	0.000695245	5	6	3	1
170.0042	-0.67587	5	35700.8	0.00269383	6	7	3	1
180.9963	-1.04919	6.5	11106.82	0.000838073	5	8	3	1
183.012	-0.76443	5.5	2163489	0.163247657	7	8	3	1
184.0197	-1.43952	5	34155.49	0.002577227	8	8	3	1
195.012	-0.71739	6.5	102706.8	0.007749815	7	9	3	1
196.0198	-0.84124	6	14610.44	0.001102441	8	9	3	1
197.0277	-0.45628	5.5	119683.7	0.009030824	9	9	3	1
198.0354	-1.08516	5	1338.89	0.000101027	10	9	3	1
207.012	-0.67581	7.5	8764.75	0.000661351	7	10	3	1
209.0276	-0.90849	6.5	23687.29	0.001787342	9	10	3	1
211.0433	-0.6629	5.5	9903.8	0.000747298	11	10	3	1
221.0277	-0.40674	7.5	2308.33	0.000174177	9	11	3	1
223.0434	-0.17889	6.5	2038.84	0.000153842	11	11	3	1
225.0589	-0.84378	5.5	13135.47	0.000991146	13	11	3	1
239.0746	-0.58517	5.5	12771.19	0.000963659	15	12	3	1
253.0903	-0.35521	5.5	3729.99	0.000281449	17	13	3	1
267.1058	-0.89815	5.5	1633.07	0.000123224	19	14	3	1
281.0282	1.459286	12.5	3408.93	0.000257223	9	16	3	1
281.1216	-0.31979	5.5	1821.95	0.000137477	21	15	3	1
295.137	-1.15167	5.5	1895.88	0.000143055	23	16	3	1
297.1527	-0.97559	4.5	59676.37	0.004502924	25	16	3	1
301.1269	0.41544	8.5	4028.46	0.00030397	21	18	2	1
309.1529	-0.29079	5.5	2388.25	0.000180207	25	17	3	1
311.1684	-0.77096	4.5	198286	0.014961814	27	17	3	1
323.1684	-0.74234	5.5	1451.48	0.000109522	27	18	3	1

325.184	-0.89149	4.5	161031	0.012150711	29	18	3	1
339.1996	-1.00206	4.5	96113.36	0.007252304	31	19	3	1

iii. Hydrocarbons

Mass	Deviation (ppm)	DBE	Area (AU)	H	C
133.0086	1.699892972	11.5	62811.9	1	11
181.0087	1.801574109	15.5	56127.39	1	15
145.0086	1.559220298	12.5	45572.59	1	12
115.0553	-0.207726155	6.5	27339.38	7	9
175.0555	1.005967691	11.5	14052.58	7	14
169.0086	1.337803535	14.5	11746.09	1	14
159.0242	1.107379852	12.5	11695.77	3	13
121.0085	1.042076643	10.5	8921.82	1	10
97.0083	-0.761789906	8.5	7849.16	1	8
157.0085	0.803141876	13.5	7737.51	1	13
195.0241	0.390208337	15.5	7076.74	3	16
91.0551	-2.458944633	4.5	6976.75	7	7
147.0242	1.197763436	11.5	6476.88	3	12
173.0398	0.728734614	12.5	5951.68	5	14
171.0243	1.614393076	13.5	5697.24	3	14
89.0394	-3.076156819	5.5	5170.44	5	7
207.0242	0.850625916	16.5	3869.71	3	17
185.0395	-0.939798457	13.5	2676.91	5	15
101.0397	0.258314373	6.5	2386.55	5	8
221.0399	1.022893293	16.5	2043.35	5	18
193.0085	0.653339528	16.5	1939.62	1	16
110.1101	0.009990001	2	1846.99	14	8
265.009	2.362566853	22.5	1787.15	1	22
219.024	-0.10912045	17.5	1741.7	3	18
189.0708	-0.919760429	11.5	1720.39	9	15
141.0709	-0.52384979	7.5	1658.86	9	11

iv. Species in LIPIDMAPS

Mass	Intensity	H	C	O	Lipid assignments
169.1233	2491.73	17	10	2	'FA 10:1'
171.1391	2313.68	19	10	2	'FA 10:0'
183.1388	1659.13	19	11	2	'FA 11:1'
227.2016	3222.71	27	14	2	'FA 14:0'
241.2172	3010.97	29	15	2	'FA 15:0'
253.2171	4602.82	29	16	2	'FA 16:1'
255.2329	311343.4	31	16	2	'FA 16:0'
261.2221	5096.02	29	18	1	'ST 18:0;O'
269.2487	2213.19	33	17	2	'FA 17:0'
281.2484	21013.3	33	18	2	'FA 18:1'
283.2642	261965.6	35	18	2	'FA 18:0'

419.1711	19105.95	27	22	8	'ST 22:5;O8'
431.2072	1619.65	31	24	7	'ST 18:3;O;GlCA/ST 18:4;O2;Hex/ST 24:5;O7'
447.2023	16996.62	31	24	8	'ST 18:3;O2;GlCA/ST 18:4;O3;Hex/ST 24:5;O8'

Supplementary Table 2. Lipid group assignments made automatically in the human serum sample, using SIMS-MFP with LIPID MAPS® database of "bulk" lipid species.

Mass (m/z)	Intensity	Assignment	Deviation (ppm)	DBE	Lipid groups
597.4852	1547942	$C_{37}H_{66}O_4Na^+$	-0.2178	4.5	DG O-34:4/MG 34:4/MG O-34:5;O
459.2481	1515874	$C_{21}H_{41}O_7PNa^+$	-0.2419	1.5	LPA 18:1/LPA O-18:2;O
780.5511	1151521	$C_{42}H_{80}NO_8PNa^+$	-0.3524	3.5	CerP 42:3;O4/LPC 34:3;O/PC 34:2/PC O-34:3;O/PE 37:2/PE O-37:3;O
671.5737	872161.2	$C_{45}H_{76}O_2Na^+$	-0.0746	7.5	CE 18:2
599.5009	869365.6	$C_{37}H_{68}O_4Na^+$	-0.1336	3.5	DG O-34:3/MG 34:3/MG O-34:4;O
625.5163	834726.9	$C_{39}H_{70}O_4Na^+$	-0.5277	4.5	DG O-36:4
879.7408	782883.4	$C_{55}H_{100}O_6Na^+$	-0.4662	5.5	CE 28:0;O4/DG 52:4;O/DG O-52:5;O2/TG 52:3/TG O-52:4;O
782.5669	643310.7	$C_{42}H_{82}NO_8PNa^+$	-0.1599	2.5	CerP 42:2;O4/LPC 34:2;O/PC 34:1/PC O-34:2;O/PE 37:1/PE O-37:2;O
881.7565	635687.6	$C_{55}H_{102}O_6Na^+$	-0.4084	4.5	DG 52:3;O/DG O-52:4;O2/TG 52:2/TG O-52:3;O
542.4906	590690.2	$C_{34}H_{65}NO_2Na^+$	-0.2748	2.5	Cer 34:2;O/NAE 32:2
808.5826	582898	$C_{44}H_{84}NO_8PNa^+$	-0.0929	3.5	CerP 44:3;O4/PC 36:2/PC O-36:3;O/PE 39:2/PE O-39:3;O
621.4850	501055.7	$C_{39}H_{66}O_4Na^+$	-0.5312	6.5	CE 12:1;O2/DG O-36:6
721.4777	469499	$C_{39}H_{71}N_0O_8PNa^+$	-0.2441	4.5	LPG O-33:5/PA 36:3/PA O-36:4;O
666.4832	376432.7	$C_{36}H_{70}NO_6PNa^+$	-0.1427	2.5	CerP 36:2;O2/LPC O-28:3/LPE O-31:3
623.5007	374497.3	$C_{39}H_{68}O_4Na^+$	-0.4492	5.5	CE 12:0;O2/DG O-36:5
487.2793	362594	$C_{23}H_{45}O_7PNa^+$	-0.4332	1.5	LPA 20:1/LPA O-20:2;O/PA O-20:1
478.3290	256273.9	$C_{29}H_{45}NO_3Na^+$	-0.3431	7.5	NAE 27:7;O
804.5517	215617.9	$C_{44}H_{80}NO_8PNa^+$	0.4038	5.5	CerP 44:5;O4/PC 36:4/PC O-36:5;O/PE 39:4/PE O-39:5;O
877.7251	191341.8	$C_{55}H_{98}O_6Na^+$	-0.5242	6.5	CE 28:1;O4/DG 52:5;O/DG O-52:6;O2/TG 52:4/TG O-52:5;O
518.3214	188496.7	$C_{24}H_{50}NO_7PNa^+$	-0.5983	0.5	LPC 16:0/LPC O-16:1;O/LPE 19:0/LPE O-19:1;O
723.4933	176029.2	$C_{39}H_{73}O_8PNa^+$	-0.3125	3.5	LPG O-33:4/PA 36:2/PA O-36:3;O
853.7250	175051.1	$C_{53}H_{98}O_6Na^+$	-0.6561	4.5	DG 50:3;O/DG O-50:4;O2/TG 50:2/TG O-50:3;O
749.5087	163238.5	$C_{41}H_{75}O_8PNa^+$	-0.6352	4.5	PA 38:3/PA O-38:4;O
725.5567	155429	$C_{39}H_{79}N_2O_6PNa^+$	-0.1297	1.5	PE-Cer 37:1;O2/SM 34:1;O2
649.5164	151706.7	$C_{41}H_{70}O_4Na^+$	-0.3543	6.5	CE 14:1;O2/DG O-38:6
855.7407	121068.6	$C_{53}H_{100}O_6Na^+$	-0.5961	3.5	DG 50:2;O/DG O-50:3;O2/TG 50:1/TG O-50:2;O

806.5670	112865.7	$C_{44}H_{82}NO_8PNa^+$	-0.0311	4.5	CerP 44:4;O4/PC 36:3/PC O-36:4;O/PE 39:3/PE O-39:4;O
722.4811	80283.96	$C_{38}H_{69}NO_{10}Na^+$	-0.3725	4.5	HexCer 32:3;O4
652.6001	68362.82	$C_{42}H_{79}NO_2Na^+$	-0.3051	3.5	Cer 42:3;O
303.2294	66453.01	$C_{18}H_{32}O_2Na^+$	-0.1652	2.5	FA 18:2
905.7565	58932.6	$C_{57}H_{102}O_6Na^+$	-0.3976	6.5	CE 30:1;O4/DG 54:5;O/DG O-54:6;O2/TG 54:4/TG O-54:5;O
907.7722	55999.47	$C_{57}H_{104}O_6Na^+$	-0.3416	5.5	CE 30:0;O4/DG 54:4;O/DG O-54:5;O2/TG 54:3/TG O-54:4;O
673.5889	53426.17	$C_{45}H_{78}O_2Na^+$	-0.7424	6.5	CE 18:1
510.2683	52896.44	$C_{24}H_{41}NO_9Na^+$	1.8537	4.5	ST 22:0;O8;G
832.5827	51443.43	$C_{46}H_{84}NO_8PNa^+$	0.0299	5.5	CerP 46:5;O4/PC 38:4/PC O-38:5;O/PE 41:4/PE O-41:5;O
468.2577	45102.01	$C_{22}H_{39}NO_8Na^+$	1.9453	3.5	CAR 15:2;O4
745.4776	42706.51	$C_{41}H_{71}O_8PNa^+$	-0.3704	6.5	PA 38:5/PA O-38:6;O
506.3602	40323.07	$C_{31}H_{49}NO_3Na^+$	-0.5216	7.5	NAE 29:7;O
523.2636	36065.95	$C_{22}H_{45}O_{10}PNa^+$	-1.2539	0.5	LPG 16:0;O
509.2478	35391.98	$C_{21}H_{43}O_{10}PNa^+$	-1.5829	0.5	LPG 15:0;O
485.2636	35124.92	$C_{23}H_{43}O_7PNa^+$	-0.5381	2.5	LPA 20:2/LPA O-20:3;O/PA O-20:2
466.2055	34824.63	$C_{21}H_{33}NO_9Na^+$	1.5999	5.5	ST 19:1;O8;G
508.2523	32734.41	$C_{24}H_{39}NO_9Na^+$	1.1725	5.5	ST 22:1;O8;G
776.5928	32402.18	$C_{44}H_{84}NO_6PNa^+$	-0.0581	3.5	CerP 44:3;O2
495.2323	28166.2	$C_{20}H_{41}O_{10}PNa^+$	-1.3248	0.5	LPG 14:0;O
443.2531	28076.86	$C_{21}H_{41}O_6PNa^+$	-0.4424	1.5	LPA O-18:2
810.5984	27569.85	$C_{44}H_{86}NO_8PNa^+$	0.0924	2.5	CerP 44:2;O4/PC 36:1/PC O-36:2;O/PE 39:1/PE O-39:2;O
522.2677	27082.08	$C_{25}H_{41}NO_9Na^+$	0.6623	5.5	ST 23:1;O8;G
470.2733	26738.39	$C_{22}H_{41}NO_8Na^+$	1.8306	2.5	CAR 15:1;O4
521.2479	25951.87	$C_{22}H_{43}O_{10}PNa^+$	-1.3546	1.5	BMP 16:0/LPG 16:1;O
627.5322	25947.93	$C_{39}H_{72}O_4Na^+$	-0.1276	3.5	DG O-36:3
747.4935	25694.17	$C_{41}H_{73}O_8PNa^+$	-0.0349	5.5	PA 38:4/PA O-38:5;O
750.5122	25413.56	$C_{40}H_{73}NO_{10}Na^+$	-0.6250	4.5	HexCer 34:3;O4
851.7092	24492.81	$C_{53}H_{96}O_6Na^+$	-0.8337	5.5	CE 26:0;O4/DG 50:4;O/DG O-50:5;O2/TG 50:3/TG O-50:4;O
626.5843	23724.68	$C_{40}H_{77}NO_2Na^+$	-0.5572	2.5	Cer 40:2;O
536.2829	23127.3	$C_{26}H_{43}NO_9Na^+$	-0.1941	5.5	ST 18:0;O4;HexNAC/ST 24:1;O8;G
724.4968	21669.66	$C_{38}H_{71}NO_{10}Na^+$	-0.3024	3.5	HexCer 32:2;O4
507.2321	21401.27	$C_{21}H_{41}O_{10}PNa^+$	-1.6878	1.5	BMP 15:0/LPG 15:1;O
524.2839	21249.69	$C_{25}H_{43}NO_9Na^+$	1.7088	4.5	ST 23:0;O8;G
480.2575	21193.84	$C_{23}H_{39}NO_8Na^+$	1.4803	4.5	CAR 16:3;O4/ST 21:0;O7;G
537.2793	21045.04	$C_{23}H_{47}O_{10}PNa^+$	-1.1281	0.5	LPG 17:0;O
835.6663	20630.22	$C_{47}H_{93}N_2O_6PNa^+$	-0.0528	2.5	PE-Cer 45:2;O2/SM 42:2;O2
546.3528	19623.92	$C_{26}H_{54}NO_7PNa^+$	-0.3846	0.5	LPC 18:0/LPC O-18:1;O/LPE 21:0/LPE O-21:1;O/PE O-21:0
494.2732	18997.13	$C_{24}H_{41}NO_8Na^+$	1.5394	4.5	CAR 17:3;O4/ST 22:0;O7;G
551.2946	18521.88	$C_{24}H_{49}O_{10}PNa^+$	-1.7343	0.5	LPG 18:0;O

483.2480	18465.82	$C_{23}H_{41}O_7PNa^+$	-0.4368	3.5	LPA 20:3/LPA O-20:4;O/PA O-20:3
481.2166	18290.46	$C_{19}H_{39}O_{10}PNa^+$	-1.4673	0.5	LPG 13:0;O
447.2406	18160.77	$C_{23}H_{38}NO_4SNa^+$	-1.7331	5	ST 23:1;O;S
419.2093	17245.78	$C_{21}H_{34}NO_4SNa^+$	-1.8490	5	ST 21:1;O;S
903.7407	17062.67	$C_{57}H_{100}O_6Na^+$	-0.5644	7.5	CE 30:2;O4/DG 54:6;O/DG O-54:7;O2/TG 54:5/TG O-54:6;O
595.4695	16856.68	$C_{37}H_{64}O_4Na^+$	-0.3025	5.5	CE 10:0;O2/DG O-34:5/MG 34:5/MG O-34:6;O
493.2167	16832	$C_{20}H_{39}O_{10}PNa^+$	-1.2289	1.5	BMP 14:0/LPG 14:1;O
695.5732	16706.24	$C_{47}H_{76}O_2Na^+$	-0.7909	9.5	CE 20:4
773.5087	16264.88	$C_{43}H_{75}O_8PNa^+$	-0.6155	6.5	PA 40:5/PA O-40:6;O
482.2733	16199.64	$C_{23}H_{41}NO_8Na^+$	1.7851	3.5	CAR 16:2;O4
578.2908	15851.31	$C_{32}H_{45}NO_5SNa^+$	-0.4584	10.5	ST 30:7;O3;T
478.2054	15515.45	$C_{22}H_{33}NO_9Na^+$	1.3507	6.5	ST 20:2;O8;G
550.2606	15338.36	$C_{30}H_{41}NO_5SNa^+$	1.5173	10.5	ST 28:7;O3;T
647.5009	15282.13	$C_{41}H_{68}O_4Na^+$	-0.1237	7.5	CE 14:2;O2/DG O-38:7
464.1900	15094.65	$C_{21}H_{31}NO_9Na^+$	1.9300	6.5	ST 19:2;O8;G
535.2634	14897.59	$C_{23}H_{45}O_{10}PNa^+$	-1.5994	1.5	BMP 17:0/LPG 17:1;O
441.2373	14757.14	$C_{21}H_{39}O_6PNa^+$	-0.7844	2.5	LPA O-18:3
540.4750	14520.16	$C_{34}H_{63}NO_2Na^+$	-0.1834	3.5	Cer 34:3;O/NAE 32:3
505.2532	14424.05	$C_{22}H_{43}O_9PNa^+$	-0.9720	1.5	LPG 16:1/LPG O-16:2;O
479.2373	14186.65	$C_{20}H_{41}O_9PNa^+$	-1.5464	0.5	LPG 14:0/LPG O-14:1;O
565.3102	14103.68	$C_{25}H_{51}O_{10}PNa^+$	-1.7797	0.5	LPG 19:0;O
750.5768	14044.44	$C_{42}H_{82}NO_6PNa^+$	-0.5264	2.5	CerP 42:2;O2/LPC O-34:3
437.1905	13352.08	$C_{17}H_{35}O_9PNa^+$	-1.3520	0.5	LPG 11:0/LPG O-11:1;O
463.2061	13298.84	$C_{19}H_{37}O_9PNa^+$	-1.3841	1.5	LPG 13:1/LPG O-13:2;O
493.2530	13021.22	$C_{21}H_{43}O_9PNa^+$	-1.4011	0.5	LPG 15:0/LPG O-15:1;O
477.2218	12746.17	$C_{20}H_{39}O_9PNa^+$	-1.2386	1.5	LPG 14:1/LPG O-14:2;O
564.2756	12467.62	$C_{31}H_{43}NO_5SNa^+$	0.3277	10.5	ST 29:7;O3;T
570.5216	12278.62	$C_{36}H_{69}NO_2Na^+$	-0.7872	2.5	Cer 36:2;O/NAE 34:2
519.2321	12137.09	$C_{22}H_{41}O_{10}PNa^+$	-1.6488	2.5	BMP 16:1/LPG 16:2;O
433.2250	11942.48	$C_{22}H_{36}NO_4SNa^+$	-1.6737	5	ST 22:1;O;S
538.2997	11208.52	$C_{26}H_{45}NO_9Na^+$	1.9430	4.5	ST 24:0;O8;G
451.2060	11129.2	$C_{18}H_{37}O_9PNa^+$	-1.6425	0.5	LPG 12:0/LPG O-12:1;O
536.2589	10937.92	$C_{22}H_{44}NO_{10}PNa^+$	-1.1284	1.5	LPS 16:0;O
550.2746	10900.72	$C_{23}H_{46}NO_{10}PNa^+$	-1.0088	1.5	LPS 17:0;O
651.5323	10810.01	$C_{41}H_{72}O_4Na^+$	0.0305	5.5	CE 14:0;O2/DG O-38:5
552.2788	10792.4	$C_{26}H_{43}NO_{10}Na^+$	1.5950	5.5	ST 18:0;O5;HexNAC
573.4857	10731.81	$C_{35}H_{66}O_4Na^+$	0.6450	2.5	DG O-32:2/MG 32:2/MG O-32:3;O
465.2217	10424.28	$C_{19}H_{39}O_9PNa^+$	-1.4855	0.5	LPG 13:0/LPG O-13:1;O
654.6158	10274.94	$C_{42}H_{81}NO_2Na^+$	-0.2278	2.5	Cer 42:2;O

494.2485	10097.97	$C_{20}H_{42}NO_9PNa^+$	-0.8904	0.5	LPS O-14:0;O
445.2251	9910.76	$C_{23}H_{36}NO_4SNa^+$	-1.4040	6	ST 23:2;O;S
484.2890	9651.26	$C_{23}H_{43}NO_8Na^+$	1.8809	2.5	CAR 16:1;O4
507.2684	9429.59	$C_{22}H_{45}O_9PNa^+$	-1.8552	0.5	LPG 16:0/LPG O-16:1;O
650.5845	9362.01	$C_{42}H_{77}NO_2Na^+$	-0.2292	4.5	Cer 42:4;O
566.2933	9230.5	$C_{27}H_{45}NO_{10}Na^+$	-0.4752	5.5	ST 19:0;O5;HexNAC
778.6083	9221.58	$C_{44}H_{86}NO_6PNa^+$	-0.2506	2.5	CerP 44:2;O2
552.3141	8985.71	$C_{27}H_{47}NO_9Na^+$	-0.3695	4.5	ST 25:0;O8;G
443.2092	8952.98	$C_{23}H_{34}NO_4SNa^+$	-1.9745	7	ST 23:3;O;S
578.3057	8931.26	$C_{25}H_{50}NO_{10}PNa^+$	-1.3057	1.5	LPS 19:0;O
548.2590	8909.32	$C_{23}H_{44}NO_{10}PNa^+$	-0.9213	2.5	LPS 17:1;O
564.2901	8663.6	$C_{24}H_{48}NO_{10}PNa^+$	-1.2495	1.5	LPS 18:0;O
687.5682	8627.48	$C_{45}H_{76}O_3Na^+$	-0.6764	7.5	CE 18:2;O
453.1851	8404.84	$C_{17}H_{35}O_{10}PNa^+$	-1.9994	0.5	LPG 11:0;O
503.2373	8399.46	$C_{22}H_{41}O_9PNa^+$	-1.4727	2.5	LPG 16:2/LPG O-16:3;O
576.2897	8376.85	$C_{25}H_{48}NO_{10}PNa^+$	-1.9176	2.5	LPS 19:1;O
496.2889	8320.76	$C_{24}H_{43}NO_8Na^+$	1.6339	3.5	CAR 17:2;O4
421.2249	8223.45	$C_{21}H_{36}NO_4SNa^+$	-1.9588	4	ST 21:0;O;S
436.2314	8090.24	$C_{21}H_{35}NO_7Na^+$	1.8933	4.5	CAR 14:3;O3/ST 19:0;O6;G
479.2007	7693.71	$C_{19}H_{37}O_{10}PNa^+$	-1.9952	1.5	BMP 13:0/LPG 13:1;O
467.2011	7659.52	$C_{18}H_{37}O_{10}PNa^+$	-1.1903	0.5	LPG 12:0;O
606.3383	7640.3	$C_{27}H_{54}NO_{10}PNa^+$	0.8987	1.5	LPS 21:0;O/PS O-21:0;O
491.2010	7612.48	$C_{20}H_{37}O_{10}PNa^+$	-1.3357	2.5	BMP 14:1/LPG 14:2;O
497.2209	7556.49	$C_{26}H_{36}NO_5SNa^+$	0.5227	9	ST 26:5;O2;S
431.2094	7454.9	$C_{22}H_{34}NO_4SNa^+$	-1.5656	6	ST 22:2;O;S
510.3043	7389.54	$C_{25}H_{45}NO_8Na^+$	1.0991	3.5	CAR 18:2;O4
424.2312	7377.99	$C_{20}H_{35}NO_7Na^+$	1.4754	3.5	CAR 13:2;O3
604.3213	7299.58	$C_{27}H_{52}NO_{10}PNa^+$	-1.3322	2.5	LPS 21:1;O/PS 21:0/PS O-21:1;O
562.2743	7245.42	$C_{24}H_{46}NO_{10}PNa^+$	-1.5208	2.5	LPS 18:1;O
457.2325	7147.71	$C_{21}H_{39}O_7PNa^+$	-0.1336	2.5	LPA 18:2/LPA O-18:3;O
827.7097	7011.44	$C_{51}H_{96}O_6Na^+$	-0.2538	3.5	DG 48:2;O/DG O-48:3;O2/TG 48:1/TG O-48:2;O
407.2093	6951.17	$C_{20}H_{34}NO_4SNa^+$	-1.9034	4	ST 20:0;O;S
481.2260	6912.66	$C_{26}H_{36}NO_4SNa^+$	0.5713	9	ST 26:5;O;S
475.2723	6866.01	$C_{25}H_{42}NO_4SNa^+$	-0.7892	5	ST 25:1;O;S
613.4796	6861.35	$C_{37}H_{66}O_5Na^+$	-1.0515	4.5	DG 34:3/DG O-34:4;O/MG 34:4;O/TG O-34:3
522.2437	6633.85	$C_{21}H_{42}NO_{10}PNa^+$	-0.2970	1.5	LPS 15:0;O
457.2254	6541.46	$C_{24}H_{36}NO_4SNa^+$	-0.7110	7	ST 24:3;O;S
534.2435	6525.23	$C_{22}H_{42}NO_{10}PNa^+$	-0.6647	2.5	LPS 16:1;O
459.2406	6494.27	$C_{24}H_{38}NO_4SNa^+$	-1.6878	6	ST 24:2;O;S

746.4815	6381.01	$C_{40}H_{69}NO_{10}Na^+$	0.1754	6.5	HexCer 34:5;O4
467.2533	5984.55	$C_{23}H_{41}O_6PNa^+$	0.0083	3.5	LPA O-20:4
558.2305	5962.57	$C_{27}H_{37}NO_{10}Na^+$	-0.8403	9.5	ST 19:4;O5;HexNAC
809.6504	5820.53	$C_{45}H_{91}N_2O_6PNa^+$	-0.3632	1.5	PE-Cer 43:1;O2/SM 40:1;O2
536.2438	5745.07	$C_{29}H_{39}NO_5SNa^+$	-0.5876	10.5	ST 27:7;O3;T
419.2557	5700.08	$C_{26}H_{36}O_3Na^+$	0.0832	8.5	ST 26:5;O3
624.5689	5697.42	$C_{40}H_{75}NO_2Na^+$	-0.1587	3.5	Cer 40:3;O
571.4696	5446.84	$C_{35}H_{64}O_4Na^+$	-0.1402	3.5	DG O-32:3/MG 32:3/MG O-32:4;O
508.2273	5391.86	$C_{20}H_{40}NO_{10}PNa^+$	-1.7809	1.5	LPS 14:0;O
778.5355	5359.71	$C_{42}H_{78}N_1O_8PNa^+$	-0.2891	4.5	CerP 42:4;O4/LPC 34:4;O/PC 34:3/PC O-34:4;O/PE 37:3/PE O-37:4;O
774.5770	5304.35	$C_{44}H_{82}NO_6PNa^+$	-0.2519	4.5	CerP 44:4;O2
473.2561	5298.84	$C_{25}H_{40}NO_4SNa^+$	-1.9548	6	ST 25:2;O;S
748.4970	5280.62	$C_{40}H_{71}NO_{10}Na^+$	-0.0255	5.5	HexCer 34:4;O4
645.4851	5221.8	$C_{41}H_{66}O_4Na^+$	-0.3565	8.5	CE 14:3;O2/DG O-38:8
461.1914	5100.13	$C_{19}H_{35}O_9PNa^+$	0.6698	2.5	LPG 13:2/LPG O-13:3;O
469.2250	5082.77	$C_{25}H_{36}NO_4SNa^+$	-1.5453	8	ST 25:4;O;S
834.5985	4980.57	$C_{46}H_{86}NO_8PNa^+$	0.2096	4.5	CerP 46:4;O4/PC 38:3/PC O-38:4;O/PE 41:3/PE O-41:4;O
520.2643	4857.49	$C_{22}H_{44}NO_9PNa^+$	-0.5576	1.5	LPS 16:0/LPS O-16:1;O
471.2843	4853.06	$C_{23}H_{45}O_6PNa^+$	-0.6283	1.5	LPA O-20:2
580.3062	4822.21	$C_{32}H_{47}NO_5SNa^+$	-0.8876	9.5	ST 30:6;O3;T
545.2879	4712.61	$C_{32}H_{42}O_6Na^+$	0.9901	11.5	ST 26:7;O;Hex
449.1902	4696.49	$C_{18}H_{35}O_9PNa^+$	-1.9838	1.5	LPG 12:1/LPG O-12:2;O
450.1739	4633.29	$C_{20}H_{29}NO_9Na^+$	0.9905	6.5	ST 18:2;O8;G
830.5665	4516.17	$C_{46}H_{82}NO_8PNa^+$	-0.6322	6.5	CerP 46:6;O4/PC 38:5/PC O-38:6;O/PE 41:5/PE O-41:6;O
566.3309	4477.09	$C_{28}H_{49}NO_9Na^+$	1.6702	4.5	ST 26:0;O8;G
694.5142	4434.42	$C_{38}H_{74}NO_6PNa^+$	-0.5689	2.5	CerP 38:2;O2/LPC O-30:3/LPE O-33:3
439.1696	4406.53	$C_{16}H_{33}O_{10}PNa^+$	-1.7217	0.5	LPG 10:0;O
449.2270	4367.58	$C_{19}H_{39}O_8PNa^+$	-1.0598	0.5	LPA 16:0;O/LPG O-13:1
465.1851	4320.91	$C_{18}H_{35}O_{10}PNa^+$	-1.9478	1.5	BMP 12:0/LPG 12:1;O
449.2198	4289.08	$C_{22}H_{36}NO_5SNa^+$	-1.8701	5	ST 22:1;O2;S
455.2098	4227	$C_{24}H_{34}NO_4SNa^+$	-0.6043	8	ST 24:4;O;S
825.6938	4199.59	$C_{51}H_{94}O_6Na^+$	-0.5572	4.5	DG 48:3;O/DG O-48:4;O2/TG 48:2/TG O-48:3;O
483.2407	4190.32	$C_{26}H_{38}NO_4SNa^+$	-1.3970	8	ST 26:4;O;S
637.4802	3917.67	$C_{39}H_{66}O_5Na^+$	-0.0708	6.5	CE 12:1;O3/DG 36:5/DG O-36:6;O/TG O-36:5
532.2277	3884.2	$C_{22}H_{40}NO_{10}PNa^+$	-0.9490	3.5	LPS 16:2;O
537.2886	3759.95	$C_{30}H_{44}NO_4SNa^+$	0.5116	9	ST 30:5;O;S
594.2863	3682.12	$C_{32}H_{45}NO_6SNa^+$	0.5383	10.5	ST 30:7;O4;T
435.2046	3615.16	$C_{21}H_{34}NO_5SNa^+$	-0.8964	5	ST 21:1;O2;S
837.6816	3613.87	$C_{47}H_{95}N_2O_6PNa^+$	-0.4705	1.5	PE-Cer 45:1;O2/SM 42:1;O2

421.1886	3607.64	$C_{20}H_{32}NO_5SNa^+$	-1.7572	5	ST 20:1;O2;S
537.2529	3542.27	$C_{29}H_{40}NO_5SNa^+$	1.7867	10	ST 29:6;O2;S
459.1746	3474.57	$C_{19}H_{33}O_9PNa^+$	-1.8318	3.5	LPG 13:3
748.5610	3469.4	$C_{42}H_{80}NO_6PNa^+$	-0.7282	3.5	CerP 42:3;O2/LPC O-34:4
756.5505	3443.01	$C_{40}H_{80}NO_8PNa^+$	-1.1567	1.5	CerP 40:1;O4/LPC 32:1;O/LPS O-34:1/PC 32:0/PC O-32:1;O/PE 35:0/PE O-35:1;O
475.2058	3402.45	$C_{20}H_{37}O_9PNa^+$	-1.9804	2.5	LPG 14:2/LPG O-14:3;O
469.2686	3334.18	$C_{23}H_{43}O_6PNa^+$	-0.7375	2.5	LPA O-20:3
647.5732	3259.65	$C_{43}H_{76}O_2Na^+$	-0.8495	5.5	CE 16:0
598.5534	3227.89	$C_{38}H_{73}NO_2Na^+$	0.0850	2.5	Cer 38:2;O
664.4678	3205.49	$C_{36}H_{68}NO_6PNa^+$	0.2331	3.5	CerP 36:3;O2/LPC O-28:4/LPE O-31:4
498.3047	3142.04	$C_{24}H_{45}NO_8Na^+$	1.9283	2.5	CAR 17:1;O4
478.2168	3134.81	$C_{19}H_{38}NO_9PNa^+$	-1.7567	1.5	LPS 13:0/LPS O-13:1;O
822.4309	3120.31	$C_{44}H_{66}NO_{10}PNa^+$	-0.9181	12.5	PS 38:10
751.5247	3097.06	$C_{41}H_{77}O_8PNa^+$	-0.1678	3.5	PA 38:2/PA O-38:3;O
901.7249	3082.14	$C_{57}H_{98}O_6Na^+$	-0.7320	8.5	CE 30:3;O4/DG 54:7;O/DG O-54:8;O2/TG 54:6/TG O-54:7;O
452.2627	2998.96	$C_{22}H_{39}NO_7Na^+$	1.8262	3.5	CAR 15:2;O3
774.5124	2979.96	$C_{42}H_{73}NO_{10}Na^+$	-0.3474	6.5	HexCer 36:5;O4
909.7874	2963.55	$C_{57}H_{106}O_6Na^+$	-0.8355	4.5	DG 54:3;O/DG O-54:4;O2/TG 54:2/TG O-54:3;O
457.1891	2915.21	$C_{23}H_{32}NO_5SNa^+$	-0.5252	8	ST 23:4;O2;S
561.3197	2886.55	$C_{33}H_{46}O_6Na^+$	1.8526	10.5	ST 27:6;O;Hex/TG 30:8
568.5056	2867.78	$C_{36}H_{67}NO_2Na^+$	-1.4056	3.5	Cer 36:3;O/NAE 34:3
833.6506	2831.62	$C_{47}H_{91}N_2O_6PNa^+$	-0.1129	3.5	PE-Cer 45:3;O2/SM 42:3;O2
584.2590	2806.9	$C_{26}H_{44}NO_{10}PNa^+$	-0.8645	5.5	LPS 20:4;O/PS 20:3/PS O-20:4;O
521.2213	2800.66	$C_{28}H_{36}NO_5SNa^+$	1.2661	11	ST 28:7;O2;S
771.4922	2724.64	$C_{43}H_{73}O_8PNa^+$	-1.7189	7.5	PA 40:6/PA O-40:7;O
875.7092	2698.43	$C_{55}H_{96}O_6Na^+$	-0.8109	7.5	CE 28:2;O4/DG 52:6;O/DG O-52:7;O2/TG 52:5/TG O-52:6;O
538.3360	2691.44	$C_{27}H_{49}NO_8Na^+$	1.7849	3.5	CAR 20:2;O4
640.5999	2658.17	$C_{41}H_{79}NO_2Na^+$	-0.6230	2.5	Cer 41:2;O
639.4960	2534.96	$C_{39}H_{68}O_5Na^+$	0.1640	5.5	CE 12:0;O3/DG 36:4/DG O-36:5;O/TG O-36:4
723.5408	2418.22	$C_{39}H_{77}N_2O_6PNa^+$	-0.4756	2.5	PE-Cer 37:2;O2/SM 34:2;O2
544.3376	2394.23	$C_{26}H_{52}NO_7PNa^+$	0.4407	1.5	LPC 18:1/LPC O-18:2;O/LPE 21:1/LPE O-21:2;O/PE O-21:1
917.5298	2375.9	$C_{52}H_{79}O_{10}PNa^+$	-0.5516	13.5	PG 46:12
613.5163	2346.66	$C_{38}H_{70}O_4Na^+$	-0.5381	3.5	DG O-35:3
618.3411	2326.9	$C_{35}H_{49}NO_7Na^+$	1.5783	11.5	ST 27:6;O2;HexNAC
722.5462	2309.97	$C_{40}H_{78}NO_6PNa^+$	0.4220	2.5	CerP 40:2;O2/LPC O-32:3
753.5873	2225.61	$C_{41}H_{83}N_2O_6PNa^+$	-1.0538	1.5	PE-Cer 39:1;O2/SM 36:1;O2
610.2820	2187.68	$C_{32}H_{45}NO_7SNa^+$	1.8105	10.5	ST 30:7;O5;T
569.4536	2144.58	$C_{35}H_{62}O_4Na^+$	-0.7553	4.5	DG O-32:4/MG 32:4/MG O-32:5;O
559.3039	2142.82	$C_{33}H_{44}O_6Na^+$	1.5911	11.5	ST 27:7;O;Hex

731.4985	2140.97	$C_{41}H_{73}O_7PNa^+$	-0.1519	5.5	PA O-38:5
514.4590	2096.53	$C_{32}H_{61}NO_2Na^+$	-0.8730	2.5	Cer 32:2;O/NAE 30:2
619.4692	2086.03	$C_{39}H_{64}O_4Na^+$	-0.7750	7.5	CE 12:2;O2/DG O-36:7
641.5112	2054.49	$C_{39}H_{70}O_5Na^+$	-0.5380	4.5	DG 36:3/DG O-36:4;O/TG O-36:3

Supplementary Table 3. First (sorted by m/z) 60 of 3,328 peaks assigned in human serum as potential protein fragments. The assignments were made by matching the elemental composition of peaks assigned by SIMS-MFP with a database of up to 6-membered peptides, calculated using known formulas of each amino acid. Multiple amino acid sequences and ion types (a/b/c) are possible for each assigned peak.

Mass (m/z)	Deviation (ppm)	DBE	Intensity	H	C	N	O	S	Na	Sequence assignment of the peptide peak
151.0479	0.67	3	64542.42	8	5	2	2	0	1	AG/GA c
151.0842	0.11	2	33405.07	12	6	2	1	0	1	VG/GV b
152.0319	0.57	3	13615.05	7	5	1	3	0	1	SS a
163.0478	0.01	4	64592.73	8	6	2	2	0	1	QG/NA/AN/GQ/AGG/GAG/GGA a
163.0842	0.10	3	237072.1	12	7	2	1	0	1	KG/GK a/PA/AP b
165.0635	0.31	3	290507.39	10	6	2	2	0	1	AA c
165.0999	0.41	2	17348.47	14	7	2	1	0	1	LG/VA/AV/GL b
166.0587	0.02	3	23661.7	9	5	3	2	0	1	NG/GN/GGG b
168.0744	0.31	2	81514.22	11	5	3	2	0	1	AG/GA a-NH3
177.0635	0.29	4	285218.19	10	7	2	2	0	1	QA/AQ/AAG/AGA/GAA a /PG/GP c
177.0999	0.38	3	88759.39	14	8	2	1	0	1	KA/AK a
179.0428	0.49	4	48862.02	8	6	2	3	0	1	NS/SN/SGG/GSG/GGS a
179.0792	0.57	3	233730.86	12	7	2	2	0	1	PS/SP b/VG/GV c
180.0744	0.29	3	146285.04	11	6	3	2	0	1	QG/NA/AN/GQ/AGG/GAG/GGA b
186.0639	0.63	6	101235.05	9	8	3	1	0	1	HA/AH a
189.0636	0.80	5	28253.7	10	8	2	2	0	1	NP/PN/PGG/GPG/GGP a
189.0748	0.63	5	24567.84	10	7	4	1	0	1	HG/GH b
189.0999	0.35	4	79096.74	14	9	2	1	0	1	PP b
191.0792	0.53	4	993401.63	12	8	2	2	0	1	NV/VN/VGG/GVG/AAA/GGV a / PA/AP c
193.0949	0.79	3	218516.9	14	8	2	2	0	1	KS/SK a /TP/PT b/LG/VA/AV/GL c
194.0538	0.97	4	39557.32	9	6	3	3	0	1	NG/GN/GGG c
194.0902	1.05	3	385964.09	13	7	3	2	0	1	QA/AQ/AAG/AGA/GAA b /PG/GP a-NH3
195.0742	0.96	3	12994.45	12	7	2	3	0	1	EA/AE b/TA/AT c
196.1058	0.78	2	15697.78	15	7	3	2	0	1	VG/GV a-NH3
203.0792	0.50	5	90741.12	12	9	2	2	0	1	QP/PQ/PAG/APG/PGA/GPA/AGP/GAP a
203.0905	0.83	5	50988.64	12	8	4	1	0	1	HA/AH b
205.1060	0.09	4	31038.31	14	8	4	1	0	1	RA/AR a
206.0537	0.43	5	68818.51	9	7	3	3	0	1	NN/NGG/GNG/GGN/GGGG a
206.0901	0.50	4	256770.27	13	8	3	2	0	1	NP/PN/PGG/GPG/GGP b
208.0693	0.18	4	202729.3	11	7	3	3	0	1	QG/NA/AN/GQ/AGG/GAG/GGA c

208.1057	0.25	3	279312.13	15	8	3	2	0	1	NV/VN/VGG/GVG/AAA/GGV b /KG/GK c/PA/AP a-NH3
214.0951	0.08	6	20899.89	13	10	3	1	0	1	HV/VH a
215.0791	0.01	6	17214.95	12	10	2	2	0	1	YG/GY b
216.0743	-0.22	6	32822.5	11	9	3	2	0	1	HT/TH a
217.0947	-0.22	5	186713.49	14	10	2	2	0	1	PAA/APA/AAP a /PP c
219.0740	-0.06	5	49085.82	12	9	2	3	0	1	PSG/SPG/PGS/GPS/SGP/GSP a
219.0852	-0.21	5	14025.58	12	8	4	2	0	1	HS/SH b
222.0849	-0.05	4	597805.78	13	8	3	3	0	1	QA/AQ/AAG/AGA/GAA c
223.0689	-0.13	4	10003.22	12	8	2	4	0	1	TSG/STG/SSA/TGS/SAS/ASS/GTS/SGT/GST a /EA/AE c
223.0801	-0.27	4	14349.11	12	7	4	3	0	1	NN/NGG/GNG/GGN/GGGG b
225.0957	-0.49	3	28434.69	14	7	4	3	0	1	QG/NA/AN/GQ/AGG/GAG/GGA a-NH3
227.0789	-0.87	7	11943.93	12	11	2	2	0	1	FG/GF c
228.1109	0.74	6	25376.83	15	11	3	1	0	1	HL/LH a
229.0695	-0.42	7	15465.57	10	9	4	2	0	1	HN/NH/HGG/GHG/GGH a
229.0947	-0.21	6	20193.56	14	11	2	2	0	1	PPG/PGP/GPP a /YA/FS/SF/AY b
229.1059	-0.35	6	55991.22	14	10	4	1	0	1	HP/PH b
231.0852	-0.20	6	131113.65	12	9	4	2	0	1	HA/AH c
233.0896	-0.27	5	93613.12	14	10	2	3	0	1	TPG/PTG/PSA/SPA/PAS/APS/TGP/SAP/ASP/GTP/PGT/GPT a
233.1008	-0.41	5	141331.99	14	9	4	2	0	1	HT/TH b
234.0961	-0.19	5	22013.49	13	8	5	2	0	1	HG/GH a-NH3
236.0640	-0.75	5	9704.15	11	8	3	4	0	1	NSG/SNG/NGS/GNS/SGN/GSN/SGGG/GSGG/GGSG/GGGS a
241.0946	-0.61	7	41374.4	14	12	2	2	0	1	FA/AF c
243.0852	-0.19	7	47233.49	12	10	4	2	0	1	HQ/QH/HAG/AHG/HGA/GHA/AGH/GAH a
244.1056	-0.19	6	165225.9	15	11	3	2	0	1	FG/GF a-NH3
245.0896	-0.26	6	17957.79	14	11	2	3	0	1	YS/SY b
246.0848	-0.46	6	165867.93	13	10	3	3	0	1	NPG/PNG/NGP/GNP/PGN/GPN/PGGG/GPGG/GGPG/GGGP a
246.0961	-0.18	6	12297.54	13	9	5	2	0	1	HN/NH/HGG/GHG/GGH b
247.0800	-0.65	6	11176.03	12	9	4	3	0	1	HD/DH b/HS/SH c/
248.1117	-0.38	5	34980.9	15	9	5	2	0	1	RN/NR/RGG/GRG/GGR a /HA/AH a-NH3
249.0957	-0.45	5	118316.54	14	9	4	3	0	1	RD/DR a

Supplementary Table 4. Assignments of abundant serum proteins, albumin, transferrin and fibronectin, enabled by SIMS-MFP and amino acid sequence search developed previously (code available on Github, https://github.com/guferraz/simsdenovo/).

Human serum albumin

Mass (m/z)	Intensity	Assignment	Deviation (ppm)	DBE	Sequence assignment
194.0902	385964.09	$C_7H_{13}N_3O_2Na^+$	0.86	3	QA a
265.1270	436399.14	$C_{10}H_{18}N_4O_3Na^+$	-0.27	4	QAA a
378.2111	396449.82	$C_{16}H_{29}N_5O_4Na^+$	-0.29	5	QAAL a
435.2324	191754.07	$C_{18}H_{32}N_6O_5Na^+$	-0.46	6	QAALG a
548.3166	51031.77	$C_{24}H_{43}N_7O_6Na^+$	-0.17	7	QAALGL a
222.0849	597805.78	$C_8H_{13}N_3O_3Na^+$	-0.22	4	QA b
293.1219	337975.09	$C_{11}H_{18}N_4O_4Na^+$	-0.33	5	QAA b
406.2059	446712.66	$C_{17}H_{29}N_5O_5Na^+$	-0.38	6	QAAL b
463.2274	139180.91	$C_{19}H_{32}N_6O_6Na^+$	-0.37	7	QAALG b
576.3113	39553.65	$C_{25}H_{43}N_7O_7Na^+$	-0.49	8	QAALGL b
239.1113	15420.07	$C_8H_{16}N_4O_3Na^+$	-0.48	3	QA c
310.1488	11375.97	$C_{11}H_{21}N_5O_4Na^+$	0.77	4	QAA c
423.2325	14456.29	$C_{17}H_{32}N_6O_5Na^+$	-0.29	5	QAAL c
480.2546	4832.96	$C_{19}H_{35}N_7O_6Na^+$	1.03	6	QAALG c
177.0635	285218.19	$C_7H_{10}N_2O_2Na^+$	0.48	4	QA a-NH3
248.1005	119002.01	$C_{10}H_{15}N_3O_3Na^+$	-0.39	5	QAA a-NH3
361.1846	827740.55	$C_{16}H_{26}N_4O_4Na^+$	-0.17	6	QAAL a-NH3
418.2059	670771.72	$C_{18}H_{29}N_5O_5Na^+$	-0.36	7	QAALG a-NH3
531.2899	96204.84	$C_{24}H_{40}N_6O_6Na^+$	-0.40	8	QAALGL a-NH3
260.1482	9245.52	$C_{11}H_{19}N_5ONa^+$	0.01	5	HK a
347.1801	101012.8	$C_{14}H_{24}N_6O_3Na^+$	-0.20	6	HKS a
476.2227	25134.05	$C_{19}H_{31}N_7O_6Na^+$	-0.22	8	HKSE a
575.2918	63260.83	$C_{24}H_{40}N_8O_7Na^+$	0.98	9	HKSEV a
646.3296	8422.98	$C_{27}H_{45}N_9O_8Na^+$	1.93	10	HKSEVA a
288.1430	58162.55	$C_{12}H_{19}N_5O_2Na^+$	-0.28	6	HK b
375.1751	22219.56	$C_{15}H_{24}N_6O_4Na^+$	-0.08	7	HKS b
504.2178	15102.82	$C_{20}H_{31}N_7O_7Na^+$	0.10	9	HKSE b
603.2874	9395.91	$C_{25}H_{40}N_8O_8Na^+$	2.03	10	HKSEV b
674.3257	5551.28	$C_{28}H_{45}N_9O_9Na^+$	3.61	11	HKSEVA b
243.1216	16945.09	$C_{11}H_{16}N_4ONa^+$	-0.23	6	HK a-NH3
330.1536	168744.97	$C_{14}H_{21}N_5O_3Na^+$	-0.03	7	HKS a-NH3
459.1962	36073.28	$C_{19}H_{28}N_6O_6Na^+$	-0.17	9	HKSE a-NH3
558.2648	26358.07	$C_{24}H_{37}N_7O_7Na^+$	0.21	10	HKSEV a-NH3
629.3020	10641.29	$C_{27}H_{42}N_8O_8Na^+$	0.42	11	HKSEVA a-NH3

Human transferrin

Mass (m/z)	Intensity	Assignment	Deviation (ppm)	DBE	Sequence assignment
531.3264	29307.12	$C_{25}H_{44}N_6O_5Na^+$	-0.29	7	NLKPV a-NH3
630.3946	3680.45	$C_{30}H_{53}N_7O_6Na^+$	-0.53	8	NLKPVV a-NH3
701.4318	1684.03	$C_{33}H_{58}N_8O_7Na^+$	-0.38	9	NLKPVVA a-NH3
349.2209	204189.6	$C_{16}H_{30}N_4O_3Na^+$	-0.21	4	LEK a-NH3
420.2580	48135.49	$C_{19}H_{35}N_5O_4Na^+$	-0.27	5	LEKA a-NH3
519.3258	40499.77	$C_{24}H_{44}N_6O_5Na^+$	-1.36	6	LEKAV a-NH3
590.3631	1955.09	$C_{27}H_{49}N_7O_6Na^+$	-0.91	7	LEKAVA a-NH3

Human fibronectin

Mass (m/z)	Intensity	Assignment	Deviation (ppm)	DBE	Sequence assignment
233.1008	141331.99	$C_9H_{14}N_4O_2Na^+$	-0.23	5	TT a
346.1848	84358.43	$C_{15}H_{25}N_5O_3Na^+$	-0.36	6	TTL a
474.2434	13685.6	$C_{20}H_{33}N_7O_5Na^+$	-0.26	8	TTLQ a
571.2963	12205.61	$C_{25}H_{40}N_8O_6Na^+$	-0.08	10	TTLQP a
628.3178	7454.45	$C_{27}H_{43}N_9O_7Na^+$	-0.01	11	TTLQPG a
216.0743	32822.5	$C_9H_{11}N_3O_2Na^+$	-0.37	6	TT a-NH3
329.1585	176485.78	$C_{15}H_{22}N_4O_3Na^+$	0.12	7	TTL a-NH3
457.2168	52780.46	$C_{20}H_{30}N_6O_5Na^+$	-0.44	9	TTLQ a-NH3
554.2691	19494.24	$C_{25}H_{37}N_7O_6Na^+$	-1.16	11	TTLQP a-NH3
611.2911	4651.97	$C_{27}H_{40}N_8O_7Na^+$	-0.13	12	TTLQPG a-NH3

Supplementary Table 5. Assignments of 50 most abundant salt ions in the human serum spectrum assigned by formula search in a human serum peak list with elemental composition within limits: C_0, $H_{1\text{-}10}$, $N_{0\text{-}0}$, $O_{0\text{-}10}$, $S_{0\text{-}1}$, $P_{0\text{-}5}$, $Na_{0\text{-}5}$, $Ca_{0\text{-}5}$, $Cl_{0\text{-}5}$

Mass (m/z)	Deviation (ppm)	DBE	Intensity	Assignment
158.9131	0.27	1	17374.73	PO_4HNaCa^+
163.9222	0.13	1.5	536719.5	$PO_4Na_3^+$
164.9301	0.58	1	35374622	$PO_4HNa_3^+$
174.8780	-0.31	1.5	15440.77	$PO_4Ca_2^+$
175.9461	2.19	0	103526.4	$PO_6H_3Na_2^+$
176.8938	0.54	0.5	2173489	$PO_4H_2Ca_2^+$
176.9237	0.44	0	119777.9	$PO_5H_3NaCa^+$
180.8946	-2.22	1.5	234310.8	$PO_4H_0Na_2Ca^+$
182.8963	1.01	1	21241.72	$PO_3Na_3Cl^+$
182.9108	0.81	0.5	980327.7	$PO_4H_2Na_2Ca^+$
186.9121	0.81	1.5	29309137	$PO_4Na_4^+$

186.9172	1.21	0	304754	$PO_6H_2NaCl^+$
189.9254	2.21	1	596262.3	$PO_7HNa_2^+$
192.8888	0.94	0.5	1822408	$PO_5H_2Ca_2^+$
198.9057	0.67	0.5	11958186	$PO_5H_2Na_2Ca^+$
204.9226	0.42	0.5	17347588	$PO_5H_2Na_4^+$
212.9149	0.71	1	4008325	$PO_7HNa_3^+$
214.8706	0.17	1	61294.92	$PO_5HNaCa_2^+$
216.8862	-0.06	0	40272.44	$PO_5H_3NaCa_2^+$
220.8874	-0.50	1	516552.2	$PO_5HNa_3Ca^+$
220.909	-0.83	0.5	252973	$P_2O_4H_3Na_4^+$
222.9031	-0.27	0	230107.3	$PO_5H_3Na_3Ca^+$
226.8857	-0.31	2	109877.6	$P_2O_6Na_3^+$
226.9044	-0.25	1	380279.4	$PO_5HNa_5^+$
228.9097	0.16	1	7492826	$PO_8HNa_3^+$
230.9254	0.37	0	138934.4	$PO_8H_3Na_3^+$
232.8811	-0.12	0	830770.7	$PO_6H_3NaCa_2^+$
234.8967	0.03	1.5	596586.5	$PO_7Na_4^+$
238.8980	-0.31	0	16007352	$PO_6H_3Na_3Ca^+$
244.8705	-0.28	1	1862251	$PO_4Na_5Cl^+$
246.9202	-0.12	0	13063913	$PO_9H_3Na_3^+$
250.8833	-0.26	1.5	93334.51	$P_2O_6HNa_4^+$
250.8916	-0.03	1.5	6897698	$PO_8Na_4^+$
254.8845	-0.97	0	112185.8	$P_2O_5H_4Na_3Ca^+$
256.8641	-0.34	0	6850488	$PO_5H_2Na_3CaCl^+$
260.8800	-0.07	0.5	1884970	$PO_6H_2Na_4Ca^+$
263.8933	0.94	0	2484055	$PO_9H_3Na_2Ca^+$
268.8723	0.17	1	1131889	$PO_8HNa_3Ca^+$
276.8665	-0.69	0.5	72838.71	$P_2O_5H_3Na_4Ca^+$
286.8683	0.05	0.5	870317.9	$PO_8HNa_4Cl^+$
288.8601	-0.43	2	3335399	$P_2O_7Na_5^+$
288.8653	0.18	0.5	112453	$P_2O_9H_2Na_2Cl^+$
300.8538	-0.14	1	1234999	$P_2O_8H_2Na_3Ca^+$
318.8345	0.04	0.5	18006.9	$P_2O_8H_3Na_2Ca_2^+$
318.8644	-0.02	0	3033530	$P_2O_9H_4Na_3Ca^+$
324.8812	-0.47	0	46885.75	$P_2O_9H_4Na_5^+$
374.8218	2.03	1	1826292	$PO_6HNa_9Ca^+$
392.8324	2.03	0	2504059	$PO_7H_3Na_9Ca^+$
396.8037	1.81	1.5	97944.77	PO_6Ca^+

Supplementary Table 6: Output from SIMSMFP on carbonaceous ions ($C_nH_{<1}^-$) found in negative ion depth profile datasets from different engine deposits. a. injector tip 1, b. injector tip 2, c. injector needle 1

a)

Mass (m/z)	Deviation (ppm)	DBE	Intensity (AU)	H	C
84.0003	-2.963075876	8	6612870.74	0	7
96.0004	-1.551032798	9	220073965.8	0	8
97.0083	-0.761789906	8.5	47997971.37	1	8
108.0005	-0.452775477	10	84130360.44	0	9
109.0084	0.239431147	9.5	4132749.92	1	9
120.0005	-0.407498136	11	78048155.01	0	10
121.0083	-0.610701537	10.5	79168732.6	1	10
132.0005	-0.370453005	12	30397142.14	0	11
133.0086	1.699892972	11.5	12830317.97	1	11
144.0004	-1.034023836	13	152560782.5	0	12
145.0084	0.179989605	12.5	80177698.65	1	12
156.0005	-0.313460436	14	25463202.22	0	13
157.0085	0.803141876	13.5	9373906.88	1	13
168.0005	-0.291070478	15	10157390.85	0	14
169.0085	0.746116876	14.5	41521754.62	1	14
180.0005	-0.271665838	16	2368939.23	0	15
181.0087	1.801574109	15.5	62770085.39	1	15
192.0005	-0.254686772	17	3750276.95	0	16
193.0084	0.135227293	16.5	17708528.08	1	16
204.0005	-0.239705238	18	12599560.26	0	17
205.0087	1.590666731	17.5	6013949.93	1	17
216.0005	-0.226388314	19	1353836.11	0	18
217.0085	0.581083567	18.5	1720664.31	1	18
228.0006	0.224122267	20	2420252.62	0	19
229.0087	1.423965397	19.5	257766.72	1	19
240.0007	0.629581893	21	409189.44	0	20
241.0086	0.938141677	20.5	268580.7	1	20
252.0008	0.996426401	22	3238500.34	0	21
264.0009	1.329921477	23	202807.35	0	22
276.0007	0.547462679	24	657326.69	0	23
300.0006	0.170333022	26	462346.94	0	25
312.0008	0.804806276	27	54765.17	0	26
324.0006	0.157715782	28	110204.74	0	27
348.0007	0.434194717	30	74614.78	0	29

b)

Mass (m/z)	Deviation (ppm)	DBE	Intensity (AU)	H	C
84.0003	-2.963075876	8	233008645	0	7
96.0004	-1.551032798	9	1577618743	0	8
97.0082	-1.792628749	8.5	566131085.7	1	8

108.0005	-0.452775477	10	423487212.1	0	9
109.0083	-0.677929569	9.5	71678193.06	1	9
120.0005	-0.407498136	11	343602244.3	0	10
121.0083	-0.610701537	10.5	399600885	1	10
132.0004	-1.128025612	12	137607509.1	0	11
133.0083	-0.555604116	11.5	45786573.46	1	11
144.0004	-1.034023836	13	428616341.1	0	12
145.0082	-1.199241088	12.5	245485284.7	1	12
156.0004	-0.954483821	14	90655083.09	0	13
157.0082	-1.107584237	13.5	21511039.46	1	13
168.0004	-0.886306628	15	45016093.82	0	14
169.0082	-1.028943099	14.5	91134843.84	1	14
180.0004	-0.8272197	16	14954606.88	0	15
181.0083	-0.408268405	15.5	11988359.59	1	15
192.0004	-0.775518616	17	18492551.57	0	16
193.0082	-0.900997177	16.5	43210306.29	1	16
204.0003	-1.220094756	18	35551840.38	0	17
205.0082	-0.848258033	17.5	4315434.88	1	17
216.0003	-1.152311887	19	12037073.81	0	18
217.0082	-0.801351565	18.5	7850087.8	1	18
228.0004	-0.653068603	20	12510637.33	0	19
229.0082	-0.759360879	19.5	1970917.51	1	19
240.0005	-0.203749534	21	4900414.46	0	20
241.0083	-0.30662835	20.5	2772360.22	1	20
252.0005	-0.194047196	22	12129226.89	0	21
253.0084	0.103158641	21.5	658870.99	1	21
264.0005	-0.185226888	23	2528673.91	0	22
265.0085	0.47583402	22.5	214436.82	1	22
276.0004	-0.539491681	24	4555222.04	0	23
277.0083	-0.266778939	23.5	247192.07	1	23
288.0005	-0.169791343	25	388118.88	0	24
289.0084	0.090308802	24.5	19991.96	1	24
300.0004	-0.496332425	26	3071755.38	0	25
301.0083	-0.24550812	25.5	44661.78	1	25
312.0005	-0.156730494	27	76152.22	0	26
324.0003	-0.768208575	28	603586.75	0	27
325.0082	-0.535063137	27.5	16584.61	1	27
336.0005	-0.145535477	29	15204.51	0	28
348.0003	-0.715228757	30	233488.43	0	29
349.0089	1.507413688	29.5	8312.38	1	29
372.0001	-1.20671865	32	24876.07	0	31
373.0078	-1.538571357	31.5	9411.76	1	31
396.0001	-1.133584287	34	11083.39	0	33
397.0084	0.065741686	33.5	5663.28	1	33
421.0089	1.24961885	35.5	9737.02	1	35
816	-0.672671116	69	6518.44	0	68

911.9995	-1.150108957	77	6587.79	0	76
935.9997	-0.906943913	79	6150.25	0	78
1032	-0.144282869	87	6210.61	0	86

c)

Mass (m/z)	Deviation (ppm)	DBE	Intensity (AU)	H	C
84.0003	-2.96308	8	220744.68	0	7
85.0082	-2.04568	7.5	88965.29	1	7
96.0005	-0.50937	9	2081249.49	0	8
97.0084	0.269049	8.5	2095862.24	1	8
108.0006	0.473146	10	427641.02	0	9
109.0085	1.156792	9.5	197975.13	1	9
120.0005	-0.4075	11	231114.71	0	10
121.0084	0.215688	10.5	666231.3	1	10
132.0006	0.38712	12	51597.92	0	11
133.0085	0.948061	11.5	69689.98	1	11
144.0005	-0.33958	13	148719.99	0	12
145.0084	0.17999	12.5	166873.93	1	12
156.0005	-0.31346	14	11353.74	0	13
157.0085	0.803142	13.5	17278.84	1	13
168.0006	0.304166	15	4698.32	0	14
169.0086	1.337804	14.5	43480.13	1	14
181.0088	2.354035	15.5	25599.2	1	15
193.0086	1.171452	16.5	12505.87	1	16
205.0087	1.590667	17.5	6963.24	1	17

Supplementary Table 7: Output from SIMSMFP on all species containing two oxygen atoms ($C_nH_nO_2^-$) found in negative ion depth profile datasets from different engine deposits. a. injector tip 1, b. injector tip 2, c. injector needle 1.

a)

Mass (m/z)	Deviation (ppm)	DBE	Intensity (AU)	H	C	O
85.0293	-2.397991175	2.5	1370786.86	5	4	2
87.045	-1.768047882	1.5	113545.11	7	4	2
92.9981	-1.117225878	5.5	344621.86	1	5	2
95.0138	-0.567285693	4.5	9766088.82	3	5	2
97.0295	-0.04019396	3.5	4687395.61	5	5	2
99.0451	-0.544196237	2.5	915249.95	7	5	2
101.0608	-0.038590629	1.5	557410.88	9	5	2
106.0061	0.670716569	6	66532.37	2	6	2
107.0139	0.430785345	5.5	2497676.93	3	6	2
108.0217	0.195331161	5	17538290.93	4	6	2
109.0296	0.881412797	4.5	34608272.52	5	6	2
111.0452	0.415146437	3.5	5720464.39	7	6	2

113.0609	0.849985111	2.5	393019.77	9	6	2
115.0765	0.400603238	1.5	128886.08	11	6	2
119.0139	0.387349863	6.5	9266409.52	3	7	2
120.0217	0.175801573	6	192608.51	4	7	2
121.0295	-0.032223548	5.5	91263656.71	5	7	2
122.0374	0.582608622	5	11642479.93	6	7	2
123.0452	0.374659209	4.5	26783866.46	7	7	2
125.0608	-0.031184831	3.5	2146662.77	9	7	2
127.0765	0.362773737	2.5	245015.27	11	7	2
129.0921	-0.030210988	1.5	128085.53	13	7	2
131.0139	0.351871185	7.5	698269.57	3	8	2
132.0217	0.159822237	7	3597370.41	4	8	2
133.0295	-0.029316805	6.5	26633213.8	5	8	2
134.0374	0.530449246	6	2325149.15	6	8	2
135.0452	0.341367303	5.5	53350172.68	7	8	2
136.053	0.155086644	5	1194002.74	8	8	2
137.0608	-0.028454525	4.5	8268800.02	9	8	2
139.0765	0.331472357	3.5	508869.49	11	8	2
141.0921	-0.027641519	2.5	345456.94	13	8	2
143.0138	-0.376886564	8.5	690911.49	3	9	2
143.1077	-0.37663927	1.5	187329.08	15	8	2
144.0217	0.146505722	8	103408.16	4	9	2
145.0295	-0.02689108	7.5	66960312.63	5	9	2
146.0373	-0.197894608	7	2886402.26	6	9	2
147.0452	0.313509142	6.5	38229562.84	7	9	2
148.053	0.142516551	6	583201.08	8	9	2
149.0608	-0.02616382	5.5	18190842.79	9	9	2
150.0686	-0.192578557	5	51051.78	10	9	2
151.0764	-0.356773002	4.5	1507044.97	11	9	2
153.0921	-0.025474861	3.5	123122.68	13	9	2
155.0139	0.297392774	9.5	70235.07	3	10	2
155.1078	0.297212737	2.5	227709.1	15	9	2
156.0217	0.135237617	9	292229.67	4	10	2
157.0295	-0.024836097	8.5	5363201.25	5	10	2
157.1234	-0.024821255	1.5	102641.27	17	9	2
158.0373	-0.182868188	8	14616324.53	6	10	2
159.0451	-0.338897469	7.5	67934079.9	7	10	2
160.053	0.131831348	7	841279.15	8	10	2
161.0608	-0.024214458	6.5	16069563.51	9	10	2
162.0687	0.438703039	6	69756.26	10	10	2
163.0765	0.282689492	5.5	4319741.06	11	10	2
165.0921	-0.023623177	4.5	374114.11	13	10	2
167.0139	0.276025006	10.5	51033.91	3	11	2
167.1079	0.874286181	3.5	63447.8	15	10	2
168.0218	0.720740328	10	52137.72	4	11	2
169.0295	-0.023072895	9.5	14368242.29	5	11	2

169.1235	0.568224136	2.5	70100.72	17	10	2
170.0374	0.418143477	9	673414.04	6	11	2
171.0452	0.269519474	8.5	36042795.12	7	11	2
171.1391	0.269371595	1.5	86044.63	19	10	2
172.053	0.122636644	8	6038646.1	8	11	2
173.0608	-0.022535432	7.5	32364607.72	9	11	2
174.0687	0.408459585	7	110152.31	10	11	2
175.0765	0.263313535	6.5	6766009.57	11	11	2
177.0921	-0.022022439	5.5	831946.41	13	11	2
179.1078	0.257386958	4.5	86495.8	15	11	2
180.0218	0.672696759	11	53101.4	4	12	2
181.0296	0.530852695	10.5	783907.56	5	12	2
181.1236	1.082687249	3.5	58836.73	17	11	2
182.0374	0.390579231	10	10125092.38	6	12	2
183.0452	0.251850426	9.5	23542331.2	7	12	2
183.1391	0.251721296	2.5	61896.11	19	11	2
184.053	0.114640904	9	2104816.94	8	12	2
185.0608	-0.021074155	8.5	27829054.48	9	12	2
186.0687	0.382117074	8	713322.75	10	12	2
187.0765	0.246423315	7.5	9680661.89	11	12	2
189.0922	0.508217942	6.5	1388969.73	13	12	2
191.1079	0.764490174	5.5	173384.08	15	12	2
193.0296	0.497851354	11.5	2033022.41	5	13	2
194.0374	0.366424339	11	144953.03	6	13	2
195.0452	0.236355526	10.5	35123799.92	7	13	2
196.053	0.10762397	10	5062305.47	8	13	2
197.0609	0.487666741	9.5	23947939.87	9	13	2
197.1547	-0.01978142	2.5	55766.83	21	12	2
198.0687	0.358966487	9	603286.59	10	13	2
199.0765	0.231569325	8.5	11645711.12	11	13	2
199.1704	0.23146015	1.5	201411.21	23	12	2
200.0843	0.105455562	8	104307.92	12	13	2
200.9986	1.970664376	14.5	4144506.24	1	14	2
201.0922	0.47789047	7.5	1941899.94	13	13	2
203.1079	0.719322612	6.5	249008.21	15	13	2
205.0298	1.444182395	12.5	372502.28	5	14	2
205.1235	0.468498466	5.5	75703.69	17	13	2
206.0374	0.345083099	12	808583.65	6	14	2
207.0453	0.705643176	11.5	5424227.8	7	14	2
208.0531	0.582063283	11	7496205.51	8	14	2
209.0609	0.45967488	10.5	36149233.92	9	14	2
210.0688	0.814495724	10	1144842.82	10	14	2
211.0766	0.692166262	9.5	12943201.22	11	14	2
211.9908	1.986410903	16	180827.36	0	15	2
212.0844	0.570999419	9	114943.38	12	14	2
213.0923	0.920259345	8.5	3224798.66	13	14	2

213.1862	0.919854007	1.5	150912.62	25	13	2
215.1079	0.679194484	7.5	384361.48	15	14	2
217.0298	1.364330631	13.5	619337.83	5	15	2
217.1235	0.442605441	6.5	100676	17	14	2
219.0453	0.666985767	12.5	11856452.61	7	15	2
220.0531	0.550322021	12	1095059.26	8	15	2
221.0609	0.434722023	11.5	19126275.94	9	15	2
222.0688	0.770482534	11	3674850.47	10	15	2
223.0766	0.654932412	10.5	18604856.98	11	15	2
224.0844	0.540421669	10	189793.4	12	15	2
225.0923	0.87119893	9.5	4348558.36	13	15	2
226.0998	-0.570101904	9	55326.29	14	15	2
227.1079	0.643306965	8.5	693649.79	15	15	2
227.2019	1.083178735	1.5	600449.28	27	14	2
229.1236	0.855870665	7.5	86977.52	17	15	2
230.0376	1.178504381	14	87711.84	6	16	2
231.0455	1.497975587	13.5	1029999.59	7	16	2
232.0532	0.952799662	13	7158741.86	8	16	2
233.061	0.841411326	12.5	15655435.8	9	16	2
234.0689	1.158207323	12	1310555.21	10	16	2
235.0767	1.046893451	11.5	15885080.07	11	16	2
236.0845	0.936529959	11	560575.64	12	16	2
237.0923	0.827104727	10.5	5782637.63	13	16	2
238.0065	1.979361624	17	115434.36	2	17	2
239.108	1.029243075	9.5	906362.57	15	16	2
241.1237	1.228001908	8.5	138032.18	17	16	2
241.2176	1.227523877	1.5	758434.11	29	15	2
243.0456	1.835461406	14.5	3920649.03	7	17	2
244.0534	1.725445032	14	161794.79	8	17	2
245.0611	1.208271561	13.5	16839310.86	9	17	2
246.0689	1.101725162	13	4026534.08	10	17	2
247.0768	1.400780991	12.5	12615759.47	11	17	2
248.0845	0.891229388	12	508334.1	12	17	2
249.0924	1.188716926	11.5	6605464.18	13	17	2
250.1001	0.684126544	11	94738.28	14	17	2
251.1081	1.378292763	10.5	1044726.48	15	17	2
253.1238	1.564849373	9.5	164502.19	17	17	2
253.2177	1.564269084	2.5	212453.93	29	16	2
255.2333	1.356016121	1.5	21770341.06	31	16	2
256.0533	1.254037353	15	1482357.34	8	18	2
257.0613	1.929893599	14.5	5213393.92	9	18	2
258.069	1.437989583	14	2298345.42	10	18	2
259.0769	1.721885541	13.5	16964829.89	11	18	2
260.0846	1.234599805	13	859755.17	12	18	2
261.0925	1.517089158	12.5	5998090.21	13	18	2
262.1	0.271270581	12	124828.65	14	18	2

263.1082	1.695503053	11.5	1519784.63	15	18	2
265.1239	1.871204098	10.5	223410.71	17	18	2
267.1395	1.669916822	9.5	64100.35	19	18	2
267.2334	1.669330049	2.5	53403.26	31	17	2
269.0613	1.843821147	15.5	8139332.96	9	19	2
269.2491	1.842535088	1.5	899511.9	33	17	2
270.069	1.374095175	15	1249119.48	10	19	2
272.0847	1.547682217	14	1254604.73	12	19	2
273.0926	1.816603237	13.5	7665320.29	13	19	2
274.1002	0.989055346	13	184187.79	14	19	2
275.1083	1.985040379	12.5	1515748.03	15	19	2
276.1155	-0.285749903	12	51648.78	16	19	2
277.1237	1.06847706	11.5	263204.79	17	19	2
278.0377	1.334712865	18	81826.58	6	20	2
279.0457	1.957030941	17.5	1117703.37	7	20	2
279.1395	1.598128222	10.5	66130.92	19	19	2
279.2333	1.239466887	3.5	55781.5	31	18	2
280.0531	0.432418182	17	304538.02	8	20	2
281.0612	1.40930359	16.5	1231788.19	9	20	2
281.2489	1.052805226	2.5	802435.68	33	18	2
282.0689	0.961113616	16	2302779.69	10	20	2
283.0768	1.222637896	15.5	8351641.21	11	20	2
283.2646	1.221827305	1.5	29122657.2	35	18	2
283.9908	1.482796712	22	57618.58	0	21	2
284.0843	0.074273733	15	770373.76	12	20	2
285.0925	1.389375555	14.5	6146068.35	13	20	2
286.0066	1.996811054	21	112882.36	2	21	2
286.1	0.248514567	14	272042.35	14	20	2
287.1082	1.553772038	13.5	2082705.7	15	20	2
288.1155	-0.273848434	13	58526.27	16	20	2
289.1239	1.715876312	12.5	334408.59	17	20	2
292.0524	-1.982174612	18	222160.71	8	21	2
293.0613	1.692822764	17.5	2847064.56	9	21	2
294.0684	-0.778389728	17	586418.83	10	21	2
295.0766	0.495125918	16.5	5218706.18	11	21	2
296.0843	0.071263493	16	1657166.45	12	21	2
296.9986	1.333678099	22.5	57881.28	1	22	2
297.0923	0.660064665	15.5	5123522.01	13	21	2
297.2799	-0.013118949	1.5	70865.68	37	19	2
298.0065	1.580840501	22	79466.47	2	22	2
298.0996	-1.103321296	15	298496.04	14	21	2
299.0144	1.826336783	21.5	1186500.56	3	22	2
299.108	0.822780409	14.5	2325102.42	15	21	2
299.2019	0.822522191	7.5	163356	27	20	2
300.0221	1.403565241	21	191122.59	4	22	2
300.1153	-0.929308638	14	69980.5	16	21	2

301.03	1.64801122	20.5	1345190.92	5	22	2
301.1237	0.98331779	13.5	406744.62	17	21	2
301.2175	0.651025016	6.5	217653.22	29	20	2
302.0377	1.228656078	20	87640.55	6	22	2
303.0457	1.802041686	19.5	1285578.38	7	22	2
303.1395	1.471601875	12.5	98492.24	19	21	2
304.053	0.069395801	19	123798.29	8	22	2
305.0613	1.626233176	18.5	1758170.09	9	22	2
305.1551	1.29803013	11.5	52305.15	21	21	2
306.0687	0.232300841	18	1184276.62	10	22	2
307.0767	0.80142908	17.5	3053513.31	11	22	2
308.0841	-0.580685261	17	903819.46	12	22	2
309.0924	0.957966885	16.5	4704320.08	13	22	2
310.0066	1.842222237	23	63516.81	2	23	2
310.0999	-0.093195765	16	501239.33	14	22	2
311.1081	1.11247629	15.5	1960060.99	15	22	2
311.2958	0.790567025	1.5	1199608	39	20	2
312.0222	1.670076265	22	152984.73	4	23	2
312.1155	-0.25279097	15	105166.45	16	22	2
313.0301	1.904293342	21.5	1427444.93	5	23	2
313.1238	1.264996468	14.5	530091.5	17	22	2
313.2176	0.94535007	7.5	497214.7	29	21	2
314.0379	1.818573614	21	153550.09	6	23	2
315.1395	1.415565587	13.5	118128.21	19	22	2
316.0524	-1.83165494	20	126622.78	8	23	2
317.061	0.618493354	19.5	1249928.9	9	23	2
319.0765	0.14447948	18.5	3737332.85	11	23	2
320.084	-0.871333016	18	834450.03	12	23	2
321.0921	-0.012146048	17.5	2614669.82	13	23	2
322.0996	-1.021111681	17	425745.81	14	23	2
323.0142	1.071471071	23.5	131262.55	3	24	2
323.1078	0.142676861	16.5	1765465.41	15	23	2
324.022	0.990983076	23	88338.31	4	24	2
324.1151	-1.477559337	16	94869.04	16	23	2
325.0299	1.218658599	22.5	1433284.25	5	24	2
325.1235	0.295580075	15.5	445106.76	17	23	2
325.3114	0.602807397	1.5	101872.39	41	21	2
326.0376	0.831499881	22	112406.99	6	24	2
327.0456	1.364031831	21.5	885407.42	7	24	2
327.1393	0.752279488	14.5	130638.05	19	23	2
328.0531	0.369147692	21	86177.48	8	24	2
329.0611	0.899833698	20.5	539014.13	9	24	2
330.0685	-0.390524845	20	359205.57	10	24	2
331.0766	0.4412878	19.5	1151111.29	11	24	2
332.084	-0.839847044	19	795810.35	12	24	2
333.0923	0.588726054	18.5	3265493.71	13	24	2

334.0996	-0.984436007	18	417264.45	14	24	2
335.108	0.734390646	17.5	1407175.79	15	24	2
336.022	0.955593107	24	68550.42	4	25	2
336.1153	-0.829774094	17	131012.88	16	24	2
337.0301	1.768687884	23.5	357967.66	5	25	2
337.1237	0.878313391	16.5	512676.5	17	24	2
338.0378	1.393633069	23	103406.21	6	25	2
339.0455	1.020807984	22.5	1182262.92	7	25	2
339.1392	0.430796743	15.5	124186.46	19	24	2
339.3268	-0.158843898	1.5	1681236	43	22	2
340.0529	-0.23202267	22	91197.08	8	25	2
341.0611	0.868173641	21.5	663588.67	9	25	2
341.1552	1.454178982	14.5	59019.95	21	24	2
343.0765	0.134372381	20.5	1524736.61	11	25	2
344.0838	-1.391810174	20	445207.87	12	25	2
345.0922	0.27847638	19.5	1682577.18	13	25	2
346.0996	-0.950303576	19	547570.74	14	25	2
347.1079	0.42090676	18.5	1582410.15	15	25	2
348.0223	1.784658938	25	69397.78	4	26	2
348.116	1.209655716	18	911232.33	16	25	2
349.0299	1.134861081	24.5	173274.12	5	26	2
349.1236	0.561692507	17.5	468566.52	17	25	2
350.0376	0.774488826	24	85746.51	6	26	2
351.0456	1.270776694	23.5	445217.84	7	26	2
351.1393	0.700861944	16.5	121591.67	19	25	2
352.0532	0.628030476	23	83150.54	8	26	2
353.0612	1.121903071	22.5	628444.53	9	26	2
353.1549	0.555280725	15.5	49458.9	21	25	2
353.3426	0.271974073	1.5	385881.81	45	23	2
354.0685	-0.364053716	22	161498.8	10	26	2
355.0767	0.693090171	21.5	562291.22	11	26	2
356.084	-0.783241543	21	538175.18	12	26	2
357.0923	0.54915804	20.5	1309585.82	13	26	2
358.0995	-1.197710375	20	387054.96	14	26	2
359.1079	0.406841675	19.5	1159544.18	15	26	2
360.0221	1.169651787	26	50123.65	4	27	2
360.1153	-0.774473576	19	198055.75	16	26	2
361.0301	1.651111595	25.5	89144.23	5	27	2
361.1236	0.543027668	18.5	466911.42	17	26	2
362.0376	0.748817811	25	61093.01	6	27	2
362.1313	0.196337665	18	69173.69	18	26	2
363.0457	1.504220602	24.5	263852.05	7	27	2
363.1394	0.953078432	17.5	125644.93	19	26	2
364.0532	0.607329188	24	71629.57	8	27	2
365.0611	0.811097759	23.5	370601.78	9	27	2
365.1551	1.084745714	16.5	55668.42	21	26	2

366.0681	-1.44481105	23	87640.3	10	27	2
367.0765	0.125586917	22.5	710126.39	11	27	2
367.3582	0.125490613	1.5	1885071.5	47	24	2
368.0837	-1.57273764	22	227202.89	12	27	2
369.0921	-0.010566468	21.5	941296.69	13	27	2
370.0995	-1.158876202	21	404327.01	14	27	2
371.1078	0.124222681	20.5	761312.34	15	27	2
371.202	0.932377311	13.5	59394.85	27	26	2
372.1151	-1.286965736	20	174550.72	16	27	2
373.03	1.329921614	26.5	52768.72	5	28	2
373.1235	0.257555541	19.5	400319.63	17	27	2
374.0375	0.457440974	26	55758.93	6	28	2
374.1307	-1.413675094	19	56773.32	18	27	2
375.0456	1.189456777	25.5	154181.49	7	28	2
375.1392	0.389455586	18.5	126754.22	19	27	2
376.0532	0.587949072	25	63400.2	8	28	2
377.0612	1.050493703	24.5	248459.9	9	28	2
378.0687	0.188061094	24	77585.86	10	28	2
379.0767	0.649209407	23.5	291072.73	11	28	2
380.0841	-0.470685082	23	267018.33	12	28	2
381.0922	0.252170011	22.5	554980.61	13	28	2
381.3739	0.251983747	1.5	695689.69	49	25	2
382.0995	-1.122481235	22	296643.37	14	28	2
383.1079	0.38135485	21.5	734445.47	15	28	2
384.1152	-0.98642185	21	203370.48	16	28	2
385.1236	0.50918744	20.5	303874.76	17	28	2
386.0378	1.220348305	27	49762.55	6	29	2
386.1307	-1.369741581	20	63394.32	18	28	2
387.0457	1.410946486	26.5	102220.02	7	29	2
387.1393	0.635688902	19.5	121490.88	19	28	2
388.0532	0.569767563	26	68593.85	8	29	2
389.0614	1.532151258	25.5	181148.33	9	29	2
389.1552	1.274814348	18.5	51770.81	21	28	2
390.0687	0.182275617	25	78416.2	10	29	2
391.0769	1.140697671	24.5	279578.37	11	29	2
392.0841	-0.456279452	24	117554.79	12	29	2
393.0924	0.75325858	23.5	507967.8	13	29	2
394.0995	-1.088302658	23	240479.69	14	29	2
395.1078	0.116677032	22.5	433519.06	15	29	2
395.3895	0.116593904	1.5	649384.13	51	26	2
396.115	-1.461442142	22	192287.37	16	29	2
397.1234	-0.009820625	21.5	279262.23	17	29	2
398.0377	0.932324616	28	46910.19	6	30	2
398.1308	-1.077282988	21	69898.9	18	29	2
399.0456	1.1179186	27.5	68240.51	7	30	2
399.1393	0.616577099	20.5	107603.07	19	29	2

400.053	0.052743014	27	59292.4	8	30	2
401.0613	1.236969545	26.5	118873.48	9	30	2
401.1548	0.239558452	19.5	50198.8	21	29	2
402.0687	0.176835482	26	60689.18	10	30	2
403.0767	0.610554146	25.5	163236.31	11	30	2
404.0841	-0.442729424	25	128930.25	12	30	2
405.0922	0.23723	24.5	246748.65	13	30	2
406.0996	-0.809899181	24	215882.83	14	30	2
407.1079	0.358873047	23.5	396799.26	15	30	2
408.1151	-1.173442095	23	163834.21	16	30	2
409.1235	0.234892453	22.5	230397.65	17	30	2
409.4053	0.478987678	1.5	275536.35	53	27	2
410.1309	-0.801938445	22	77437.29	18	30	2
411.0458	1.571846776	28.5	46933.87	7	31	2
411.1393	0.598580937	21.5	97401.25	19	30	2
412.053	0.051207008	28	50192.2	8	31	2
413.0615	1.685224048	27.5	92750.76	9	31	2
414.0688	0.413216525	27	60088.05	10	31	2
415.077	1.315661235	26.5	149344.5	11	31	2
416.0841	-0.42996097	26	92102.48	12	31	2
417.0924	0.709915142	25.5	268158.78	13	31	2
418.0999	-0.069122231	25	143366.93	14	31	2
419.108	0.587199826	24.5	273900.77	15	31	2
420.1153	-0.663864932	24	166717.55	16	31	2
421.1236	0.46565923	23.5	201997.97	17	31	2
422.1315	0.642217352	23	96127.51	18	31	2
423.1393	0.581605498	22.5	99249.64	19	31	2
423.421	0.581218558	1.5	370966.17	55	28	2
426.0688	0.401578498	28	58625.35	10	32	2
427.077	1.278693768	27.5	98679.44	11	32	2
428.084	-0.651507224	27	73841.95	12	32	2
429.0925	0.923111836	26.5	140042.42	13	32	2
430.0996	-0.764706009	26	152557.51	14	32	2
431.1079	0.338894392	25.5	214853.35	15	32	2
432.115	-1.339687871	25	134215.94	16	32	2
433.1236	0.452757801	24.5	174074.33	17	32	2
434.131	-0.527259927	24	83207.15	18	32	2
435.1393	0.565566335	23.5	77616.51	19	32	2
436.0533	0.736378412	30	45748.81	8	33	2
436.1473	0.965500432	23	51646.4	20	32	2
437.0614	1.363883457	29.5	52119.7	9	33	2
437.1554	1.592342468	22.5	54888.17	21	32	2
437.4365	0.219689077	1.5	345253.36	57	29	2
438.0688	0.390578071	29	58837.24	10	33	2
439.077	1.243746949	28.5	71110.68	11	33	2
440.0844	0.275174565	28	53024.35	12	33	2

441.0929	1.804838475	27.5	130792.61	13	33	2
442.0998	-0.291563042	27	95302.8	14	33	2
443.1081	0.781074122	26.5	163824.9	15	33	2
444.1154	-0.402823068	26	120308.41	16	33	2
445.1236	0.440551987	25.5	137440.12	17	33	2
446.1309	-0.737227028	25	85454.18	18	33	2
447.1393	0.550388068	24.5	90272.81	19	33	2
449.1553	1.327159651	23.5	58592.3	21	33	2
451.0772	1.65404333	29.5	61508.45	11	34	2
451.4523	0.545129871	1.5	440708.05	59	30	2
452.0845	0.489068101	29	67686.31	12	34	2
453.0927	1.315626547	28.5	81506.73	13	34	2
454.1002	0.597005158	28	89030.97	14	34	2
456.1153	-0.611467823	27	110786.1	16	34	2
457.1238	0.866505623	26.5	131415.86	17	34	2
458.131	-0.4996385	26	71724.69	18	34	2
459.1395	0.971601079	25.5	71370.66	19	34	2
460.1477	1.784430228	25	56129.99	20	34	2
461.1555	1.726318724	24.5	50820.01	21	34	2
462.0692	1.235963587	31	50713.06	10	35	2
464.0841	-0.385490326	30	49273.25	12	35	2
465.4678	0.206459007	1.5	306080.4	61	31	2
466.0997	-0.491096406	29	85634.8	14	35	2
467.1083	1.169109259	28.5	102548.4	15	35	2
468.1154	-0.382170575	28	84436.22	16	35	2
469.1237	0.631177207	27.5	109584.03	17	35	2
470.1317	1.00206064	27	88303.12	18	35	2
471.1394	0.734602655	26.5	71925.62	19	35	2
472.1464	-1.014303009	26	47692.1	20	35	2
477.093	1.87825368	30.5	57377.97	13	36	2
478.1001	0.357874975	30	90538.41	14	36	2
479.4835	0.304702966	1.5	357146.62	63	32	2
480.1157	0.252230932	29	63950.21	16	36	2
481.1243	1.862515922	28.5	90211.99	17	36	2
482.1313	0.147470223	28	85373.84	18	36	2
483.14	1.958235403	27.5	62161.12	19	36	2
493.4993	0.600001211	1.5	397937.35	65	33	2
494.1315	0.548639681	29	77602.1	18	37	2
502.1004	0.938259444	32	55442	14	38	2
507.5146	-0.10620383	1.5	374050	67	34	2
518.1321	1.681234312	31	77025.92	18	39	2
520.1471	0.425072242	30	52639.3	20	39	2
521.5304	0.184265419	1.5	290001.02	69	35	2
526.1	0.135145428	34	60869.73	14	40	2
528.1166	1.933478278	33	63252.96	16	40	2
530.1312	-0.054514804	32	70646.9	18	40	2

532.1475	1.167158964	31	50981.2	20	40	2
535.5461	0.272805721	1.5	321750.18	71	36	2
549.562	0.720756321	1.5	338382.56	73	37	2
554.132	1.391547633	34	71158.26	18	42	2
563.5775	0.436674827	1.5	289994.22	75	38	2
564.1163	1.278284144	36	49738.62	16	43	2
566.1319	1.185414204	35	76083.3	18	43	2
576.1167	1.945963694	37	73981.72	16	44	2
577.5932	0.512644802	1.5	170402.93	77	39	2
590.1314	0.289935512	37	57637.58	18	45	2
591.6089	0.585015206	1.5	190141.92	79	40	2
594.1635	1.634401284	35	46071.5	22	45	2
602.132	1.28061785	38	74153.56	18	46	2
604.1478	1.524629245	37	64430.92	20	46	2
605.0615	1.150462888	43.5	66227.28	9	47	2
605.6246	0.654035947	1.5	238251.24	81	41	2
616.1473	0.683440936	38	66796.18	20	47	2
619.6403	0.719934325	1.5	192396.52	83	42	2
624.1166	1.636075167	41	68595.17	16	48	2
630.1632	1.064963353	38	73795.34	22	48	2
632.1793	1.773392435	37	45896.59	24	48	2
633.6565	1.571989818	1.5	105934.85	85	43	2
644.1782	0.032754913	38	50484.92	24	49	2
647.6712	0.071178097	1.5	119540.39	87	44	2
648.1165	1.421197129	43	60053.62	16	50	2
656.1784	0.336951162	39	56141.38	24	50	2
659.2025	1.283522265	37.5	49314.82	27	50	2
661.6873	0.749750482	1.5	128173.3	89	45	2
665.154	-1.058250052	41.5	79494.28	21	51	2
666.1621	-0.643836874	41	69559.95	22	51	2
675.1381	-1.412894121	43.5	61167.9	19	52	2
675.7028	0.5122077	1.5	103748.63	91	46	2
676.1459	-1.447762358	43	87274.29	20	52	2
681.1866	0.87509138	40.5	56649.98	25	52	2
684.2093	-0.261469631	39	54755.34	28	52	2
688.1461	-1.131880452	44	80288.63	20	53	2
689.1534	-1.892028006	43.5	56763.24	21	53	2
689.7177	-0.585601563	1.5	55542.41	93	47	2
690.1615	-1.490808262	43	69251.81	22	53	2
692.1787	0.752840838	42	47005.37	24	53	2
694.1943	0.678628908	41	46388.2	26	53	2
695.1091	1.936534289	47.5	50140.71	15	54	2
698.1309	-0.471114865	46	82980.56	18	54	2
702.1619	-0.89566158	44	84680.02	22	54	2
703.1693	-1.498783323	43.5	49481.73	23	54	2
703.7342	0.633904509	1.5	59833.43	95	48	2

704.1769	-1.816159657	43	69570.5	24	54	2
706.1945	0.95030567	42	61867.57	26	54	2
711.1382	-1.200749692	46.5	60750.74	19	55	2
714.163	0.659653764	45	56841.1	22	55	2
716.1768	-1.925358857	44	70949.19	24	55	2
717.7499	0.691188314	1.5	75422.15	97	49	2
718.1929	-1.293383433	43	64133.12	26	55	2
724.7115	1.684949193	5	49721.44	92	50	2
727.1698	-0.761719722	45.5	48398.77	23	56	2
728.1778	-0.520339679	45	68222.71	24	56	2
730.193	-1.135178041	44	68044.67	26	56	2
731.7661	1.429557198	1.5	65571.75	99	50	2
732.1149	-0.927312599	50	49517.39	16	57	2
732.2095	0.02881689	43	52767.64	28	56	2
734.1305	-0.992874259	49	49774.33	18	57	2
739.17	-0.478780024	46.5	50217.17	23	57	2
740.1771	-1.457622003	46	55376.08	24	57	2
742.1936	-0.308410002	45	65647.77	26	57	2
745.2166	-0.944556704	43.5	63061.59	29	57	2
750.1618	-0.971656104	48	47957.57	22	58	2
759.1382	-1.124826862	50.5	47658.5	19	59	2
759.7962	-0.202554275	1.5	56165.65	103	52	2
762.1618	-0.956357696	49	50626.98	22	59	2
773.8118	-0.263500694	1.5	70874.84	105	53	2
774.1625	-0.037330662	50	54845.81	22	60	2
776.1771	-1.390015887	49	53364.88	24	60	2
779.201	-0.839192264	47.5	64203.36	27	60	2
784.2414	0.791976159	45	49040.25	32	60	2
804.1147	-1.09300208	56	55340.78	16	63	2
812.1768	-1.697780162	52	49296.64	24	63	2
813.1846	-1.726419286	51.5	58187.84	25	63	2
814.1954	1.929638796	51	76217.29	26	63	2
823.1692	-1.401775458	53.5	51457.63	23	64	2
824.1774	-0.945062633	53	48705.13	24	64	2
828.8684	1.95580284	2	53928.34	112	57	2
844.2423	1.80173718	50	52979.6	32	65	2
849.5629	1.525610378	26.5	45582.95	73	62	2
864.2084	-1.248424168	54	78885.96	28	67	2
885.9379	0.785721603	1.5	52637.84	121	61	2
904.1485	1.792960898	62	67150.25	20	71	2
914.3185	-0.578463297	50	47191.47	42	70	2
921.1866	0.647100583	60.5	55533.92	25	72	2
941.9988	-1.065711475	1.5	48017.9	129	65	2
948.2109	1.498719462	61	46231.97	28	74	2
960.2079	-1.644328696	62	60707.51	28	75	2
978.2557	-0.74510116	60	47645.62	34	76	2

1060.898	0.208408333	19	56938.83	116	76	2
1065.282	1.592163706	65.5	53788.39	37	83	2
1110.256	-0.116099305	71	97550.39	34	87	2
1110.536	-1.82695213	50	79565.7	70	84	2

b)

Mass (m/z)	Deviation (ppm)	DBE	Intensity (AU)	H	C	O
87.045	-1.76805	1.5	32632.89	7	4	2
92.9981	-1.11723	5.5	2510406.04	1	5	2
95.0138	-0.56729	4.5	49008497.38	3	5	2
96.0216	-0.82169	4	12130.25	4	5	2
97.0294	-1.07081	3.5	4460634.73	5	5	2
99.0451	-0.5442	2.5	150446.18	7	5	2
101.0607	-1.02809	1.5	11398.67	9	5	2
101.061	1.940416	1.5	8125.29	9	5	2
106.0061	0.670717	6	675314.98	2	6	2
107.0139	0.430785	5.5	5655704.61	3	6	2
108.0217	0.195331	5	120220762.5	4	6	2
109.0295	-0.03577	4.5	66956522.15	5	6	2
111.0452	0.415146	3.5	1337277.62	7	6	2
113.0609	0.849985	2.5	78093.24	9	6	2
115.0766	1.269591	1.5	14617.69	11	6	2
116.9983	0.82138	7.5	71533.86	1	7	2
118.0062	1.449926	7	20693.16	2	7	2
119.0139	0.38735	6.5	24789821.88	3	7	2
120.0217	0.175802	6	878751.67	4	7	2
121.0295	-0.03222	5.5	252231185.3	5	7	2
122.0373	-0.23681	5	43257141.72	6	7	2
123.0451	-0.43805	4.5	32022317.56	7	7	2
125.0608	-0.03118	3.5	145471.67	9	7	2
127.0765	0.362774	2.5	58241.47	11	7	2
129.0921	-0.03021	1.5	12137.16	13	7	2
130.006	-0.2223	8	23874.04	2	8	2
131.0138	-0.41141	7.5	2487904.19	3	8	2
132.0216	-0.59763	7	12130109.7	4	8	2
133.0295	-0.02932	6.5	44765706.06	5	8	2
134.0373	-0.21561	6	8182091.54	6	8	2
135.0452	0.341367	5.5	76581959.15	7	8	2
136.0529	-0.57992	5	6879289.98	8	8	2
137.0607	-0.75806	4.5	4381121.36	9	8	2
139.0765	0.331472	3.5	13913.28	11	8	2
141.0921	-0.02764	2.5	47854.72	13	8	2
143.0138	-0.37689	8.5	1796093.43	3	9	2
143.1077	-0.37664	1.5	14201.31	15	8	2

144.0216	-0.54783	8	474223.11	4	9	2
145.0295	-0.02689	7.5	114156368.9	5	9	2
146.0372	-0.88265	7	4045198.57	6	9	2
147.0451	-0.36655	6.5	45339388.22	7	9	2
148.0529	-0.53292	6	1314491.57	8	9	2
149.0607	-0.69703	5.5	11187649.3	9	9	2
150.0686	-0.19258	5	244792.55	10	9	2
151.0764	-0.35677	4.5	400249.75	11	9	2
155.0138	-0.34771	9.5	157080.87	3	10	2
155.1078	0.297213	2.5	31482.26	15	9	2
156.0216	-0.5057	9	1559949.42	4	10	2
157.0294	-0.66166	8.5	8274169.94	5	10	2
157.1234	-0.02482	1.5	9629.3	17	9	2
158.0372	-0.81563	8	23696459.75	6	10	2
159.0451	-0.3389	7.5	77592226.83	7	10	2
160.0529	-0.49296	7	916042.15	8	10	2
161.0607	-0.6451	6.5	9037669.24	9	10	2
162.0686	-0.17832	6	50341.25	10	10	2
163.0764	-0.33052	5.5	1198070.65	11	10	2
165.0921	-0.02362	4.5	24272.68	13	10	2
167.0138	-0.32273	10.5	102325.37	3	11	2
168.0216	-0.46958	10	165697	4	11	2
169.0294	-0.61469	9.5	20645875.57	5	11	2
169.1234	-0.02306	2.5	31011.5	17	10	2
170.0372	-0.75807	9	1768963.56	6	11	2
171.0451	-0.31512	8.5	44238379.03	7	11	2
171.1391	0.269372	1.5	10114.23	19	10	2
172.0529	-0.45858	8	5900749.93	8	11	2
173.0607	-0.60037	7.5	21358168.03	9	11	2
174.0685	-0.74051	7	73386.81	10	11	2
175.0763	-0.87904	6.5	1964114.89	11	11	2
177.092	-0.5867	5.5	180989.33	13	11	2
180.0216	-0.43828	11	150736.05	4	12	2
181.0294	-0.57394	10.5	2849268.18	5	12	2
182.0373	-0.15876	10	14336632.62	6	12	2
183.045	-0.84078	9.5	26254119.26	7	12	2
183.1389	-0.84035	2.5	24588.34	19	11	2
184.0528	-0.972	9	3081070.06	8	12	2
185.0607	-0.56144	8.5	22518910.69	9	12	2
186.0685	-0.69276	8	601306.81	10	12	2
187.0763	-0.82266	7.5	4011019.01	11	12	2
188.9985	1.566682	13.5	48900.04	1	13	2
189.092	-0.54947	6.5	326063.41	13	12	2
191.1078	0.241225	5.5	24362.51	15	12	2
192.0218	0.630658	12	20411.09	4	13	2
193.0293	-1.05632	11.5	4327688.56	5	13	2

194.0372	-0.66431	11	575715.45	6	13	2
195.0451	-0.27635	10.5	49550039.74	7	13	2
196.0528	-0.91251	10	5788051.92	8	13	2
197.0607	-0.52725	9.5	24802793.38	9	13	2
197.1547	-0.01978	2.5	19968.97	21	12	2
198.0685	-0.65078	9	1194307.31	10	13	2
199.0763	-0.77307	8.5	6722982.27	11	13	2
199.1703	-0.27062	1.5	65472.89	23	12	2
200.0842	-0.39433	8	23589.9	12	13	2
200.9985	1.473147	14.5	41356.27	1	14	2
201.092	-0.51668	7.5	636427.46	13	13	2
203.0141	1.212233	13.5	11916.62	3	14	2
203.1077	-0.26538	6.5	37918.91	15	13	2
204.0215	-0.87687	13	6269.89	4	14	2
205.0294	-0.50676	12.5	316742.36	5	14	2
205.1233	-0.50652	5.5	7179.14	17	13	2
206.0372	-0.62561	12	1979681.34	6	14	2
207.045	-0.74332	11.5	7317074.77	7	14	2
208.0528	-0.85988	11	10209412.19	8	14	2
209.0608	-0.01865	10.5	37916630.53	9	14	2
210.0685	-0.61361	10	1918871.28	10	14	2
211.0763	-0.72912	9.5	11287152.92	11	14	2
211.1704	0.218307	2.5	19042.96	23	13	2
212.0842	-0.37202	9	324750.9	12	14	2
212.9985	1.390153	15.5	140452.96	1	15	2
213.092	-0.48758	8.5	2027252.72	13	14	2
213.186	-0.01829	1.5	18655.4	25	13	2
214.0063	1.266787	15	27146.14	2	15	2
215.0141	1.144577	14.5	15668.3	3	15	2
215.1077	-0.25057	7.5	80077.25	15	14	2
217.0294	-0.47874	13.5	376867.64	5	15	2
217.1233	-0.47853	6.5	6636.58	17	14	2
218.0371	-1.04982	13	117694.22	6	15	2
219.045	-0.70259	12.5	10626841.42	7	15	2
219.1391	0.210369	5.5	6321.13	19	14	2
220.0528	-0.81299	12	2070468.12	8	15	2
221.0607	-0.47001	11.5	15813691.29	9	15	2
222.0685	-0.58045	11	4427309.4	10	15	2
223.0764	-0.24162	10.5	13743211.18	11	15	2
224.0842	-0.3521	10	346952.05	12	15	2
224.9985	1.316011	16.5	245240.34	1	16	2
225.092	-0.46159	9.5	3443446.1	13	15	2
225.186	-0.01732	2.5	25874.01	25	14	2
226.0999	-0.12782	9	44019.08	14	15	2
227.0141	1.084075	15.5	26856.84	3	16	2
227.1077	-0.23733	8.5	549726.93	15	15	2

227.2017	0.202903	1.5	81030.38	27	14	2
229.0296	0.419597	14.5	36405.05	5	16	2
229.1234	-0.01702	7.5	9596.46	17	15	2
230.0373	-0.12563	14	179071.39	6	16	2
231.0451	-0.23329	13.5	1396611.69	7	16	2
232.0529	-0.34001	13	6949066.55	8	16	2
233.0607	-0.44581	12.5	11787259.72	9	16	2
234.0686	-0.12347	12	1936728.04	10	16	2
235.0764	-0.22929	11.5	11007783.05	11	16	2
236.0842	-0.3342	11	704971.95	12	16	2
236.9985	1.249377	17.5	156344.72	1	17	2
237.0921	-0.01645	10.5	3684971.5	13	16	2
238.0999	-0.12138	10	30877.27	14	16	2
239.0143	1.866419	16.5	51656.92	3	17	2
239.1078	0.1928	9.5	845141.3	15	16	2
239.2016	-0.22533	2.5	16020.28	27	15	2
240.1157	0.50434	9	12280.82	16	16	2
241.0298	1.22848	15.5	35873.76	5	17	2
241.1235	0.398551	8.5	76501.86	17	16	2
241.2174	0.398396	1.5	44780.96	29	15	2
242.0369	-1.77204	15	24900.97	6	17	2
243.0451	-0.22177	14.5	2102534.18	7	17	2
243.1392	0.600891	7.5	8987.04	19	16	2
244.0529	-0.32329	14	494283.4	8	17	2
245.0608	-0.01591	13.5	11147638.04	9	17	2
246.0686	-0.11745	13	3653689.78	10	17	2
247.0764	-0.21815	12.5	8174385.12	11	17	2
248.0843	0.085052	12	662778.62	12	17	2
248.9986	1.590775	18.5	2431429.2	1	18	2
249.0921	-0.01566	11.5	4257498.35	13	17	2
250.1	0.284286	11	41693.26	14	17	2
251.0143	1.777193	17.5	79128.72	3	18	2
251.1078	0.183587	10.5	856869.36	15	17	2
253.03	1.960641	16.5	20407.25	5	18	2
253.1235	0.379657	9.5	128597.26	17	17	2
253.2175	0.774434	2.5	137916.96	29	16	2
254.0374	0.27988	16	26772.09	6	18	2
255.0453	0.57284	15.5	176412.36	7	18	2
255.1392	0.572629	8.5	8928.1	19	17	2
255.2333	1.356016	1.5	14970289.85	31	16	2
256.0531	0.472949	15	1896887.14	8	18	2
257.0609	0.373842	14.5	3569888.69	9	18	2
258.0686	-0.11199	14	3012741.24	10	18	2
259.0765	0.17794	13.5	11210041.19	11	18	2
260.0843	0.081128	13	883593.16	12	18	2
261.0921	-0.01494	12.5	3923854.55	13	18	2

262.1001	0.652804	12	71385.25	14	18	2
263.0143	1.696108	18.5	396429.49	3	19	2
263.1078	0.175213	11.5	1130472.19	15	18	2
265.03	1.871867	17.5	48377.66	5	19	2
265.1236	0.739656	10.5	140471.84	17	18	2
266.0373	-0.10863	17	11182.84	6	19	2
267.0454	0.921567	16.5	273838.66	7	19	2
267.1392	0.546906	9.5	21775.32	19	18	2
267.2332	0.920919	2.5	15715.72	31	17	2
268.0531	0.451776	16	99053.7	8	19	2
269.0609	0.357168	15.5	4659488.77	9	19	2
269.2489	1.099727	1.5	59864.39	33	17	2
270.0688	0.633543	15	1385371.11	10	19	2
271.0766	0.538962	14.5	4991019.39	11	19	2
272.0845	0.812616	14	1662027.92	12	19	2
273.0923	0.718073	13.5	5351710.97	13	19	2
274.1001	0.624225	13	131000.15	14	19	2
275.108	0.894559	12.5	1256905.35	15	19	2
276.1157	0.438584	12	11467.6	16	19	2
277.0299	1.429812	18.5	99159.93	5	20	2
277.1235	0.346777	11.5	221947.93	17	19	2
278.0374	0.255721	18	7321.45	6	20	2
279.0453	0.523571	17.5	59181.17	7	20	2
279.1389	-0.55134	10.5	13571.49	19	19	2
279.2332	0.881343	3.5	21760.78	31	18	2
280.053	0.075343	17	268648.35	8	20	2
281.0609	0.341919	16.5	761454.89	9	20	2
281.2488	0.697248	2.5	392415.55	33	18	2
282.0687	0.252066	16	2328321.5	10	20	2
283.0765	0.162854	15.5	4554968.19	11	20	2
283.2646	1.221827	1.5	17489172.75	35	18	2
284.0843	0.074274	15	729186.35	12	20	2
285.0922	0.337084	14.5	3392643.77	13	20	2
286.0065	1.647168	21	26543.92	2	21	2
286.1	0.248515	14	268389.05	14	20	2
287.0144	1.902696	20.5	72116.08	3	21	2
287.1079	0.508868	13.5	1444429.7	15	20	2
288.1158	0.7674	13	11602.78	16	20	2
289.1236	0.678257	12.5	261659.2	17	20	2
291.0455	1.189163	18.5	77684.68	7	21	2
291.1393	0.845301	11.5	25514.6	19	20	2
292.0528	-0.61256	18	20966.62	8	21	2
293.061	0.669144	17.5	1267911.87	9	21	2
294.0688	0.581837	17	381047.67	10	21	2
295.0764	-0.18266	16.5	2906295.74	11	21	2
295.2644	0.494811	2.5	6470.21	35	19	2

296.0842	-0.26648	16	1473565.64	12	21	2
297.092	-0.34972	15.5	2599834.63	13	21	2
297.2798	-0.3495	1.5	18174.55	37	19	2
298.0999	-0.09695	15	185363.47	14	21	2
299.0142	1.157471	21.5	248396.96	3	22	2
299.1077	-0.1802	14.5	1300994.26	15	21	2
300.1157	0.403511	14	23822.73	16	21	2
301.0298	0.983625	20.5	68930.86	5	22	2
301.1234	-0.01295	13.5	145014.63	17	21	2
302.0378	1.559741	20	6877.83	6	22	2
303.0455	1.142074	19.5	26266.01	7	22	2
303.1391	0.152075	12.5	40091.46	19	21	2
304.0531	0.398286	19	38696.83	8	22	2
305.0609	0.315019	18.5	150574.59	9	22	2
306.0687	0.232301	18	950890.19	10	22	2
307.0765	0.150125	17.5	1521620.13	11	22	2
308.0844	0.393074	17	628065.16	12	22	2
309.0922	0.310911	16.5	2486017.03	13	22	2
310.1001	0.551758	16	318412.41	14	22	2
311.1079	0.469612	15.5	1050477.64	15	22	2
311.2959	1.111805	1.5	29696.6	39	20	2
312.1157	0.387997	15	20994.44	16	22	2
313.0301	1.904293	21.5	138871.09	5	23	2
313.1236	0.626271	14.5	296210.58	17	22	2
315.0457	1.733402	20.5	45095.05	7	23	2
315.1394	1.098245	13.5	38281.87	19	22	2
316.0534	1.332372	20	17517.63	8	23	2
317.0607	-0.3277	19.5	214814.65	9	23	2
318.0685	-0.40526	19	73409.79	10	23	2
319.0763	-0.48233	18.5	1360523.58	11	23	2
320.0842	-0.2465	18	564754.84	12	23	2
321.092	-0.32358	17.5	1226269.12	13	23	2
322.0999	-0.08972	17	266446.6	14	23	2
323.1077	-0.16682	16.5	1154236.24	15	23	2
324.1155	-0.24343	16	21577.94	16	23	2
325.0299	1.218659	22.5	97476.77	5	24	2
325.1235	0.29558	15.5	236047.08	17	23	2
327.0455	1.058264	21.5	46582.47	7	24	2
327.1391	0.140919	14.5	34855.46	19	23	2
328.0533	0.978805	21	14119.97	8	24	2
329.0609	0.292043	20.5	30280.62	9	24	2
330.0687	0.21541	20	197858.59	10	24	2
331.0765	0.139243	19.5	331405.1	11	24	2
332.0843	0.063538	19	432145.83	12	24	2
333.0921	-0.01171	18.5	1241361.03	13	24	2
334.1	0.212811	18	168764.26	14	24	2

335.1079	0.435979	17.5	691055.72	15	24	2
336.1158	0.657809	17	36622.66	16	24	2
337.1236	0.581686	16.5	221767.69	17	24	2
339.0455	1.020808	22.5	60750.65	7	25	2
339.139	-0.15893	15.5	38203.15	19	24	2
340.0535	1.532408	22	6537.05	8	25	2
341.0609	0.281768	21.5	63540.65	9	25	2
342.0685	-0.37682	21	28117.36	10	25	2
343.0764	-0.15711	20.5	475315.49	11	25	2
344.0843	0.061322	20	167060.55	12	25	2
345.0921	-0.0113	19.5	615015.93	13	25	2
346.0999	-0.0835	19	256687.33	14	25	2
347.1078	0.132812	18.5	616183	15	25	2
348.1159	0.922395	18	33900.87	16	25	2
349.1235	0.275261	17.5	189718.21	17	25	2
351.0458	1.840504	23.5	25801.66	7	26	2
351.1392	0.416074	16.5	35387.31	19	25	2
353.0611	0.838666	22.5	26102.84	9	26	2
353.1547	-0.01104	15.5	6038.1	21	25	2
354.0688	0.48324	22	58678.11	10	26	2
355.0766	0.411461	21.5	94076.74	11	26	2
355.1705	0.411352	14.5	5983.27	23	25	2
356.0844	0.340088	21	230552.81	12	26	2
357.0922	0.269118	20.5	493303.4	13	26	2
358.1	0.198548	20	113401.71	14	26	2
359.1079	0.406842	19.5	424582.8	15	26	2
360.1157	0.336281	19	52408.39	16	26	2
361.1236	0.543028	18.5	150588.62	17	26	2
363.1393	0.677702	17.5	28467.87	19	26	2
365.0611	0.811098	23.5	25151.87	9	27	2
365.1551	1.084746	16.5	6775.92	21	26	2
366.0693	1.833263	23	9817.83	10	27	2
367.0765	0.125587	22.5	138272.51	11	27	2
368.0844	0.329001	22	45524.88	12	27	2
369.0921	-0.01057	21.5	307562.38	13	27	2
370.0999	-0.07809	21	150865.76	14	27	2
371.1078	0.124223	20.5	250871.15	15	27	2
372.1157	0.325437	20	33608.99	16	27	2
373.1235	0.257556	19.5	125919.84	17	27	2
375.1391	0.122888	18.5	32609.15	19	27	2
376.0536	1.651629	25	9223.65	8	28	2
377.1547	-0.01034	17.5	7719.24	21	27	2
378.0691	1.24607	24	24221.94	10	28	2
379.0768	0.913008	23.5	23852.87	11	28	2
380.0846	0.844813	23	85827.74	12	28	2
381.0922	0.25217	22.5	134110.51	13	28	2

382.1002	0.7095	22	75169.62	14	28	2
383.1079	0.381355	21.5	216212.65	15	28	2
384.1157	0.31527	21	37682.73	16	28	2
385.1237	0.768844	20.5	74988.58	17	28	2
386.1312	-0.07485	20	6129.21	18	28	2
387.1393	0.635689	19.5	25825.89	19	28	2
389.0613	1.275122	25.5	20003.62	9	29	2
391.0768	0.884993	24.5	43154.76	11	29	2
392.0849	1.584098	24	23648.01	12	29	2
392.9989	1.771255	30.5	6989.24	1	30	2
393.0924	0.753259	23.5	120684.37	13	29	2
394.1001	0.434154	23	59161.15	14	29	2
395.1078	0.116677	22.5	107121.07	15	29	2
396.1157	0.305719	22	30011	16	29	2
397.1235	0.24199	21.5	74052.74	17	29	2
399.1391	0.115499	20.5	17255.75	19	29	2
403.0767	0.610554	25.5	19305.73	11	30	2
404.0848	1.289582	25	28274.86	12	30	2
405.0924	0.730945	24.5	38206.46	13	30	2
406.1003	0.913814	24	65094.2	14	30	2
407.1079	0.358873	23.5	85202.13	15	30	2
408.116	1.031816	23	28409.62	16	30	2
409.1235	0.234892	22.5	44096.6	17	30	2
410.131	-0.55811	22	9462.91	18	30	2
411.1393	0.598581	21.5	20547.47	19	30	2
412.0536	1.50733	28	5817.52	8	31	2
413.1551	0.958721	20.5	6933.02	21	30	2
415.077	1.315661	26.5	24020.68	11	31	2
416.0844	0.291047	26	13538.58	12	31	2
417.0924	0.709915	25.5	56594.9	13	31	2
419.108	0.5872	24.5	62135.78	15	31	2
420.1162	1.478403	24	33215.02	16	31	2
421.1238	0.940579	23.5	32196.27	17	31	2
422.1319	1.58979	23	8484.87	18	31	2
423.1393	0.581605	22.5	16912.36	19	31	2
427.0772	1.746994	27.5	17924.5	11	32	2
428.0843	0.049289	27	20325.16	12	32	2
429.0924	0.690062	26.5	24798.53	13	32	2
430.1004	1.095327	26	28203.43	14	32	2
431.1077	-0.12503	25.5	43857.5	15	32	2
432.1161	1.205927	25	22955.82	16	32	2
433.1237	0.683639	24.5	34002.76	17	32	2
434.1317	1.085156	24	7441.36	18	32	2
435.1386	-1.04311	23.5	15138.02	19	32	2
436.0537	1.653698	30	6879.57	8	33	2
437.1553	1.36359	22.5	6906.11	21	32	2

441.0928	1.578128	27.5	41175.94	13	33	2
442.1004	1.065596	27	17435.93	14	33	2
443.1074	-0.79868	26.5	42317.62	15	33	2
444.1163	1.623676	26	26291.48	16	33	2
445.1235	0.215895	25.5	26311.45	17	33	2
446.132	1.728415	25	8147.39	18	33	2
447.1388	-0.56783	24.5	10065.93	19	33	2
454.1007	1.698084	28	21468.72	14	34	2
457.1239	1.085265	26.5	27919.32	17	34	2
459.1394	0.753802	25.5	13270.7	19	34	2
466.1008	1.868913	29	11721.4	14	35	2
468.1165	1.967676	28	22916.73	16	35	2
469.1238	0.844341	27.5	24721.86	17	35	2
470.1315	0.576648	27	11165.4	18	35	2
471.1397	1.371357	26.5	9657.76	19	35	2
473.1552	1.048494	25.5	5776.8	21	35	2
480.1163	1.50193	29	18352.92	16	36	2
482.132	1.599357	28	13010.22	18	36	2
483.1397	1.337296	27.5	11533.48	19	36	2
485.1541	-1.24476	26.5	10602.79	21	36	2
490.1005	1.165273	31	6011.26	14	37	2
491.1086	1.72284	30.5	33391.99	15	37	2
492.1162	1.262102	30	7636.52	16	37	2
495.1396	1.102922	28.5	9400.03	19	37	2
497.154	-1.41586	27.5	6996.11	21	37	2
506.1315	0.535632	30	12836.12	18	38	2
509.0616	1.56386	35.5	5762.2	9	39	2
512.0852	1.798727	34	5919.4	12	39	2
519.139	-0.10383	30.5	22516.3	19	39	2
526.1001	0.325223	34	12470.83	14	40	2
528.1163	1.365421	33	11926.15	16	40	2
531.1395	0.839893	31.5	18465.45	19	40	2
536.085	1.345124	36	6987.35	12	41	2
538.101	1.990522	35	6377.42	14	41	2
556.1473	0.757174	33	9536.26	20	42	2
564.1153	-0.4944	36	10620.19	16	43	2
566.1319	1.185414	35	7494.61	18	43	2
569.1557	1.750139	33.5	9730.4	21	43	2
572.0838	-0.83711	39	8037.04	12	44	2
582.0694	1.324758	41	6111.26	10	45	2
586.1011	1.998123	39	7901.07	14	45	2
589.1238	0.672355	37.5	14347.41	17	45	2
591.1393	0.416315	36.5	15094.61	19	45	2
600.1159	0.535064	39	9230.81	16	46	2
606.1626	0.117295	36	9901.37	22	46	2
614.1302	-1.67537	39	11384.74	18	47	2

624.1152	-0.6071	41	12996.99	16	48	2
628.1472	0.511186	39	9881.82	20	48	2
638.1315	0.424834	41	10566.55	18	49	2
648.1143	-1.97326	43	8873.36	16	50	2
650.132	1.186068	42	8885.27	18	50	2
652.1474	0.799053	41	8068.4	20	50	2
654.1633	1.178759	40	7201.51	22	50	2
660.1161	0.789407	44	14544.18	16	51	2
664.1463	-0.87164	42	13138.97	20	51	2
666.1624	-0.1935	41	8310.93	22	51	2
676.1456	-1.89145	43	11329.65	20	52	2
688.1472	0.466615	44	7751.35	20	53	2
698.1308	-0.61435	46	6086	18	54	2
701.1557	1.420657	44.5	6861.29	21	54	2
702.1637	1.667847	44	7910.27	22	54	2
712.1482	1.855095	46	6414.92	20	55	2
724.1468	-0.10896	47	8048.51	20	56	2
728.178	-0.24568	45	8582.44	24	56	2
730.1938	-0.03958	44	6572.63	26	56	2
738.1617	-1.12292	47	7958.88	22	57	2
740.1769	-1.72783	46	9127.52	24	57	2
742.1929	-1.25156	45	7282.56	26	57	2
750.1613	-1.63818	48	8023.76	22	58	2
754.1952	1.817968	46	6902.09	26	58	2
762.164	1.930166	49	10972.5	22	59	2
800.1788	0.776202	51	8680.43	24	62	2
804.2104	1.145348	49	7788.46	28	62	2
810.1619	-0.77626	53	8727.41	22	63	2
824.1779	-0.3384	53	7563.64	24	64	2
914.2243	-0.90667	57	5690.78	30	71	2
924.2092	-0.30177	59	6627.59	28	72	2
938.2241	-1.09665	59	6559.14	30	73	2
963.2332	0.255494	60.5	8650.98	31	75	2
1045.31	-1.63004	61.5	5910.21	41	81	2
1067.295	-0.42528	64.5	11825.45	39	83	2
1080.303	-0.07304	65	5961.58	40	84	2

c)

Mass (m/z)	Deviation (ppm)	DBE	Intensity (AU)	H	C	O
85.0293	-2.39799	2.5	5050.53	5	4	2
92.9981	-1.11723	5.5	8640.46	1	5	2
95.0138	-0.56729	4.5	163947	3	5	2
97.0294	-1.07081	3.5	26981.66	5	5	2
107.0139	0.430785	5.5	9091.68	3	6	2
108.0217	0.195331	5	263177.4	4	6	2

109.0295	-0.03577	4.5	135806.2	5	6	2
111.0452	0.415146	3.5	9863.44	7	6	2
119.0138	-0.45289	6.5	102630.2	3	7	2
120.0217	0.175802	6	6527.03	4	7	2
121.0295	-0.03222	5.5	784922.7	5	7	2
122.0373	-0.23681	5	24277.07	6	7	2
123.0452	0.374659	4.5	58050.05	7	7	2
127.0764	-0.42415	2.5	9011	11	7	2
131.0138	-0.41141	7.5	3218.14	3	8	2
132.0216	-0.59763	7	46426.82	4	8	2
133.0295	-0.02932	6.5	160342.8	5	8	2
134.0373	-0.21561	6	24906.3	6	8	2
135.0451	-0.39913	5.5	1110759	7	8	2
137.0607	-0.75806	4.5	5380.82	9	8	2
141.0921	-0.02764	2.5	20434.78	13	8	2
143.0138	-0.37689	8.5	8595.77	3	9	2
145.0294	-0.71641	7.5	911478.3	5	9	2
146.0372	-0.88265	7	13643.75	6	9	2
147.0451	-0.36655	6.5	278396.5	7	9	2
148.053	0.142517	6	4084.72	8	9	2
149.0607	-0.69703	5.5	121129.8	9	9	2
157.0294	-0.66166	8.5	23894.17	5	10	2
158.0372	-0.81563	8	164557.9	6	10	2
159.0451	-0.3389	7.5	572742.6	7	10	2
161.0608	-0.02421	6.5	64698.88	9	10	2
163.0764	-0.33052	5.5	17548.85	11	10	2
169.0295	-0.02307	9.5	148194.7	5	11	2
170.0373	-0.16996	9	2779.99	6	11	2
171.0451	-0.31512	8.5	294621.6	7	11	2
172.053	0.122637	8	32948.41	8	11	2
173.0607	-0.60037	7.5	154942.7	9	11	2
175.0764	-0.30787	6.5	11991.8	11	11	2
177.0921	-0.02202	5.5	10203.86	13	11	2
181.0295	-0.02154	10.5	3428.19	5	12	2
182.0374	0.390579	10	73657.85	6	12	2
183.0451	-0.29446	9.5	137611.7	7	12	2
184.053	0.114641	9	16921.41	8	12	2
185.0607	-0.56144	8.5	144204.4	9	12	2
187.0764	-0.28812	7.5	14766.9	11	12	2
191.1078	0.241225	5.5	6478.64	15	12	2
193.0295	-0.0202	11.5	17307.5	5	13	2
195.0451	-0.27635	10.5	243982.1	7	13	2
196.053	0.107624	10	14768.3	8	13	2
197.0608	-0.01979	9.5	93692.02	9	13	2
198.0686	-0.14591	9	2814.82	10	13	2
199.0765	0.231569	8.5	30070.84	11	13	2

200.9985	1.473147	14.5	3200	1	14	2
203.0142	1.70481	13.5	4005.04	3	14	2
205.1235	0.468498	5.5	3800.02	17	13	2
206.0374	0.345083	12	5692.27	6	14	2
207.0452	0.222657	11.5	22011.63	7	14	2
208.053	0.101416	11	42398.91	8	14	2
209.0608	-0.01865	10.5	138609.8	9	14	2
210.0687	0.338461	10	5496.41	10	14	2
211.0765	0.218404	9.5	67111.91	11	14	2
217.0297	0.903564	13.5	3096.86	5	15	2
219.0452	0.210459	12.5	67372.44	7	15	2
220.053	0.095886	12	4789.21	8	15	2
221.0609	0.434722	11.5	82464.53	9	15	2
222.0687	0.320171	11	8889.72	10	15	2
223.0765	0.206656	10.5	35554.05	11	15	2
225.0922	0.426936	9.5	4172.78	13	15	2
231.0454	1.06516	13.5	2973.94	7	16	2
232.0531	0.521864	13	34340.85	8	16	2
233.0609	0.412339	12.5	43526.96	9	16	2
233.1548	0.412173	5.5	4146.5	21	15	2
234.0687	0.303757	12	6649.54	10	16	2
235.0766	0.6215	11.5	48522.84	11	16	2
237.0922	0.405328	10.5	6049.93	13	16	2
243.0453	0.601123	14.5	9284.64	7	17	2
245.061	0.80021	13.5	69178.84	9	17	2
246.0689	1.101725	13	7189.9	10	17	2
247.0766	0.591315	12.5	29720.69	11	17	2
249.0924	1.188717	11.5	12812.06	13	17	2
256.0532	0.863493	15	7627.48	8	18	2
257.0612	1.540881	14.5	10530.73	9	18	2
258.0689	1.050496	14	8817.35	10	18	2
259.0767	0.949913	13.5	36562.56	11	18	2
261.0924	1.134083	12.5	5859.32	13	18	2
269.0612	1.472158	15.5	31064.06	9	19	2
271.0768	1.276762	14.5	23825.15	11	19	2
273.0925	1.450426	13.5	7217.03	13	19	2
282.0688	0.60659	16	7777.3	10	20	2
283.0767	0.869376	15.5	15255.86	11	20	2
285.0923	0.687848	14.5	8391.99	13	20	2
293.0611	1.010371	17.5	5405.45	9	21	2
295.0765	0.156231	16.5	13105	11	21	2
297.0922	0.323469	15.5	6582.56	13	21	2
309.0921	-0.01262	16.5	4581.85	13	22	2
319.0764	-0.16893	18.5	6547.05	11	23	2

Supplementary Table 8: Output from SIMSMFP on all sulfonated species ($C_nH_nSO_3^-$) found in negative ion depth profile datasets from different engine deposits. a. injector tip 1, b. injector tip 2, c. injector needle 1.

a)

Mass (m/z)	Deviation (ppm)	DBE	Intensity (AU)	H	C	O	S
94.9808	-0.420084725	0.5	261373.59	3	1	3	1
106.9809	0.561782839	1.5	129002.6	3	2	3	1
108.9966	1.010124272	0.5	86666.45	5	2	3	1
120.9964	-0.74299676	1.5	51336.2	5	3	3	1
123.0122	0.488569665	0.5	112313.66	7	3	3	1
155.9884	-1.698200316	5	514526.43	4	6	3	1
156.9965	0.064332648	4.5	4905764.45	5	6	3	1
165.9727	-1.897296378	7	54647.67	2	7	3	1
170.0041	-1.264085563	5	8642757.7	6	7	3	1
171.0122	0.351437038	4.5	7770542.57	7	7	3	1
179.9884	-1.471759348	7	215954.85	4	8	3	1
180.9964	-0.496694715	6.5	504599.65	5	8	3	1
182.004	-1.730178761	6	1633013.89	6	8	3	1
183.0121	-0.218018324	5.5	141185515.7	7	8	3	1
184.0198	-0.896098421	5	4768474.31	8	8	3	1
185.0279	0.59504575	4.5	149150.56	9	8	3	1
191.9884	-1.379768957	8	73957.05	4	9	3	1
194.004	-1.623159775	7	3651125.19	6	9	3	1
195.0122	0.308185942	6.5	2232912.79	7	9	3	1
196.0198	-0.84124084	6	4518536.76	8	9	3	1
197.0279	0.558804421	5.5	5889233.53	9	9	3	1
198.0353	-1.590118021	5	450776.03	10	9	3	1
206.004	-1.528608758	8	1789043.18	6	10	3	1
207.0121	-0.19274232	7.5	443281.45	7	10	3	1
208.0197	-1.273435462	7	2634609.47	8	10	3	1
209.0279	0.526724222	6.5	13389238.08	9	10	3	1
210.0353	-1.499269541	6	3005210.96	10	10	3	1
211.0436	0.758611592	5.5	620065.12	11	10	3	1
218.004	-1.444466822	9	583541.52	6	11	3	1
220.0197	-1.203981648	8	5633611.37	8	11	3	1
221.0279	0.498127408	7.5	367301.03	9	11	3	1
222.0354	-0.967862746	7	1121719.73	10	11	3	1
223.0435	0.269454237	6.5	1126679.25	11	11	3	1
224.051	-1.182318699	6	486228.23	12	11	3	1
225.059	-0.399450651	5.5	877724.88	13	11	3	1
230.0042	-0.499555846	10	244845.09	6	12	3	1
231.0119	-1.038473563	9.5	292789.85	7	12	3	1
232.0196	-1.572709487	9	2679452.26	8	12	3	1
233.028	0.901609203	8.5	6192113.08	9	12	3	1
234.0354	-0.918236312	8	4144505.16	10	12	3	1
234.1293	-0.917868045	1	57271.17	22	11	3	1
235.0437	1.106603954	7.5	184100.2	11	12	3	1

236.0509	-1.545850645	7	1945855.88	12	12	3	1
237.0591	0.042605411	6.5	234055.77	13	12	3	1
238.0665	-1.742787318	6	3204620.22	14	12	3	1
239.075	1.087944298	5.5	908240.48	15	12	3	1
240.0821	-1.936417166	5	2293880.34	16	12	3	1
242.0043	-0.06156915	11	72926.15	6	13	3	1
242.0978	-1.713767283	4	430383.1	18	12	3	1
243.0121	-0.164189328	10.5	78745.32	7	13	3	1
244.0199	-0.265961845	10	2261087.22	8	13	3	1
244.1135	-1.494794244	3	176207.31	20	12	3	1
245.0277	-0.366897159	9.5	135539.44	9	13	3	1
246.0355	-0.46700556	9	1987727.63	10	13	3	1
246.1291	-1.685697874	2	167600.15	22	12	3	1
248.0512	-0.261639464	8	710262.41	12	13	3	1
248.1448	-1.470510216	1	146361.33	24	12	3	1
249.0588	-1.163980805	7.5	203898.84	13	13	3	1
250.0667	-0.859369982	7	245514.21	14	13	3	1
251.0745	-0.955492377	6.5	816037.41	15	13	3	1
252.0822	-1.447541603	6	465611.5	16	13	3	1
253.0905	0.435022444	5.5	547578.17	17	13	3	1
254.0044	0.335033679	12	51305.21	6	14	3	1
254.0979	-1.239284582	5	718169.71	18	13	3	1
255.0124	1.019951443	11.5	75634.71	7	14	3	1
255.1058	-0.940393258	4.5	48493.42	19	13	3	1
256.02	0.137098683	11	754968.79	8	14	3	1
256.1136	-1.034305582	4	156492.67	20	13	3	1
257.0279	0.42835835	10.5	311800.3	9	14	3	1
258.0356	-0.057743967	10	2716975.76	10	14	3	1
258.1294	-0.445125386	3	54171.49	22	13	3	1
259.0434	-0.15402822	9.5	130235.78	11	14	3	1
260.0512	-0.249566177	9	2173572.54	12	14	3	1
261.0586	-1.876586639	8.5	199613.79	13	14	3	1
262.0667	-0.820019574	8	1924422	14	14	3	1
263.0747	-0.151667925	7.5	81159.9	15	14	3	1
264.0823	-1.003095377	7	1908615.45	16	14	3	1
265.0906	0.792559851	6.5	310650.91	17	14	3	1
266.098	-0.807596549	6	468105.99	18	14	3	1
267.0126	1.723142626	12.5	51235.91	7	15	3	1
267.1063	0.973770567	5.5	234859.39	19	14	3	1
268.0201	0.504066927	12	511258.9	8	15	3	1
268.1137	-0.615037197	5	165512.75	20	14	3	1
269.0282	1.524377835	11.5	104144.08	9	15	3	1
270.0357	0.315143615	11	2582643.49	10	15	3	1
270.1292	-1.16573711	4	66318.94	22	14	3	1
271.0438	1.328569325	10.5	166619.54	11	15	3	1
272.0514	0.496597581	10	991200.32	12	15	3	1

272.145	-0.605926621	3	53103.06	24	14	3	1
273.0591	0.036988331	9.5	131410.49	13	15	3	1
274.067	0.310508111	9	376499.58	14	15	3	1
275.0745	-0.872126609	8.5	393037.77	15	15	3	1
276.0823	-0.959495578	8	243170.22	16	15	3	1
277.09	-1.407122059	7.5	286926.09	17	15	3	1
278.0978	-1.491918962	7	480990.74	18	15	3	1
279.1061	0.215330345	6.5	116820.85	19	15	3	1
280.0199	-0.231769188	13	219786.3	8	16	3	1
280.1135	-1.302684536	6	186446.52	20	15	3	1
281.028	0.747612896	12.5	260817.56	9	16	3	1
281.1219	0.74736318	5.5	160669.64	21	15	3	1
282.0355	-0.407395357	12	1344841.97	10	16	3	1
282.1292	-1.116154048	5	52881.08	22	15	3	1
283.0436	0.565637558	11.5	188267.27	11	16	3	1
284.0512	-0.228479884	11	2464660.86	12	16	3	1
285.0595	1.438649089	10.5	78606.51	13	16	3	1
286.0667	-0.751222839	10	1405087.16	14	16	3	1
287.0751	1.254377171	9.5	68547.05	15	16	3	1
288.0823	-0.919528053	9	1443678.34	16	16	3	1
290.098	-0.7407836	8	521613.2	18	16	3	1
291.106	-0.137063457	7.5	77671.13	19	16	3	1
291.2004	1.580014062	0.5	51973.11	31	15	3	1
292.0202	0.80508194	14	100077.29	8	17	3	1
292.1136	-0.906838161	7	146163.73	20	16	3	1
293.122	1.057922394	6.5	72925.86	21	16	3	1
294.0357	0.289420722	13	1292145.6	10	17	3	1
294.1293	-0.730630519	6	64152.45	22	16	3	1
295.0435	0.203698818	12.5	175075.09	11	17	3	1
295.1375	0.542459317	5.5	161630.07	23	16	3	1
296.0511	-0.556998127	12	1309967.94	12	17	3	1
297.0591	0.03399997	11.5	140930.05	13	17	3	1
297.1532	0.707043197	4.5	1738427.73	25	16	3	1
298.0667	-0.720979046	11	629919.73	14	17	3	1
299.0745	-0.802140629	10.5	157535.58	15	17	3	1
300.0823	-0.882757051	10	195072.46	16	17	3	1
301.0901	-0.962833786	9.5	238337.35	17	17	3	1
302.0978	-1.373394411	9	301179.34	18	17	3	1
303.1058	-0.791472186	8.5	68034.14	19	17	3	1
304.0201	0.444378711	15	91314.69	8	18	3	1
304.1135	-1.199879526	8	205149.56	20	17	3	1
305.0282	1.344467664	14.5	82749.99	9	18	3	1
305.2158	0.688365886	0.5	82267.79	33	16	3	1
306.0355	-0.375446498	14	524107.65	10	18	3	1
306.1292	-1.02864959	7	62049.07	22	17	3	1
307.0437	0.847111406	13.5	222771.1	11	18	3	1

307.1376	0.846852421	6.5	58172.15	23	17	3	1
308.0511	-0.535300512	13	1693945.61	12	18	3	1
309.0596	1.650493438	12.5	111657.77	13	18	3	1
309.1533	1.003063241	5.5	226643.29	25	17	3	1
310.0668	-0.370565173	12	1450304.07	14	18	3	1
311.1689	0.835881148	4.5	14513907.43	27	17	3	1
312.0823	-0.848813839	11	1027687.77	16	18	3	1
313.0909	1.629241958	10.5	48643.8	17	18	3	1
314.0979	-1.002552657	10	576457.83	18	18	3	1
315.1063	0.825436415	9.5	48362.04	19	18	3	1
318.0354	-0.675710486	15	265197.81	10	19	3	1
318.1289	-1.932860584	8	56900.84	22	18	3	1
319.0438	1.128686426	14.5	88635.29	11	19	3	1
319.2312	-0.124987767	0.5	72543.83	35	17	3	1
320.051	-0.827679903	14	788120.88	12	19	3	1
321.0592	0.342927528	13.5	148364.39	13	19	3	1
322.0666	-0.977747125	13	546664.31	14	19	3	1
323.0747	-0.123500835	12.5	103513.8	15	19	3	1
323.1687	0.185971015	5.5	206446	27	18	3	1
324.0823	-0.817384299	12	222583.98	16	19	3	1
325.0901	-0.891751983	11.5	153514.74	17	19	3	1
325.1844	0.338577242	4.5	16960296.04	29	18	3	1
326.0978	-1.272316072	11	156397.35	18	19	3	1
327.106	-0.121978793	10.5	63592.44	19	19	3	1
328.1134	-1.416886178	10	146004.87	20	19	3	1
330.0354	-0.651141848	16	143744.04	10	20	3	1
330.1292	-0.95386806	9	61468.58	22	19	3	1
331.0438	1.08777265	15.5	123458.7	11	20	3	1
332.0511	-0.496610064	15	593290.66	12	20	3	1
333.0596	1.531560061	14.5	110457.53	13	20	3	1
333.2473	1.230619137	0.5	69943.32	37	18	3	1
334.0667	-0.643284296	14	958405.41	14	20	3	1
334.2541	-1.839615038	0	50802.7	38	18	3	1
335.0753	1.671567365	13.5	55788.79	15	20	3	1
336.0823	-0.788199174	13	701764.14	16	20	3	1
337.1845	0.623101391	5.5	136200.17	29	19	3	1
338.0979	-0.931386166	12	477642.44	18	20	3	1
339.2	0.177181635	4.5	6239694.28	31	19	3	1
340.1133	-1.660914354	11	130392.74	20	20	3	1
342.0356	-0.043562715	17	75819.28	10	21	3	1
342.129	-1.50498562	10	56089.23	22	20	3	1
343.0439	1.341229554	16.5	62972.34	11	21	3	1
344.0511	-0.479289039	16	373787.13	12	21	3	1
345.0595	1.188492093	15.5	107713.77	13	21	3	1
346.0667	-0.620978171	15	386268.1	14	21	3	1
347.075	0.749406309	14.5	112298.69	15	21	3	1

348.0826	0.100838144	14	3774944.25	16	21	3	1
349.0906	0.601849853	13.5	84705.78	17	21	3	1
350.0979	-0.899461884	13	128879.47	18	21	3	1
351.106	-0.113640882	12.5	79597.43	19	21	3	1
351.2	0.171127592	5.5	79729.82	31	20	3	1
352.1134	-1.320311542	12	126597.34	20	21	3	1
354.0357	0.240371297	18	65176.01	10	22	3	1
354.1292	-0.88922269	11	63907.33	22	21	3	1
356.0511	-0.463135555	17	217657.22	12	22	3	1
357.0596	1.428615079	16.5	98215.94	13	22	3	1
358.0668	-0.320889742	16	583451.77	14	22	3	1
359.0754	1.838335941	15.5	68978.29	15	22	3	1
360.0824	-0.457950526	15	483244.8	16	22	3	1
362.0979	-0.869653556	14	373951.78	18	22	3	1
364.1135	-1.002159037	13	160929.89	20	22	3	1
365.2157	0.301465773	5.5	63505.06	33	21	3	1
366.1289	-1.679460341	12	57665.28	22	22	3	1
368.0512	-0.176334131	18	134523.93	12	23	3	1
369.0596	1.382163491	17.5	75368.22	13	23	3	1
370.0667	-0.58070579	17	258332.04	14	23	3	1
371.0751	0.970424449	16.5	86815.16	15	23	3	1
372.0824	-0.443181207	16	178903.16	16	23	3	1
374.098	-0.574448077	15	112004.21	18	23	3	1
376.1135	-0.97018492	14	102930.09	20	23	3	1
378.1291	-1.097243097	13	58249.44	22	23	3	1
379.2314	0.422169861	5.5	184481.72	35	22	3	1
380.0513	0.092355961	19	89982.23	12	24	3	1
381.247	0.288789241	4.5	9662373.54	37	22	3	1
382.0668	-0.300732661	18	272405.64	14	24	3	1
384.0824	-0.429334771	17	343753.12	16	24	3	1
386.0979	-0.815595587	16	316014.57	18	24	3	1
388.1135	-0.940188004	15	157230.41	20	24	3	1
390.1294	-0.294517578	14	60786.9	22	24	3	1
392.0517	1.109803842	20	58962.85	12	25	3	1
393.247	0.279976785	5.5	57952.11	37	23	3	1
394.0668	-0.291574846	19	150920.78	14	25	3	1
395.0753	1.417706432	18.5	67698.03	15	25	3	1
396.0826	0.088617887	18	139302.36	16	25	3	1
398.0983	0.213766344	17	106090.03	18	25	3	1
400.1137	-0.412132682	16	79313.46	20	25	3	1
402.1292	-0.783081043	15	57559.4	22	25	3	1
404.0513	0.086870165	21	62247.49	12	26	3	1
406.0668	-0.28295829	20	136513.89	14	26	3	1
407.2627	0.393112512	5.5	5732989.9	39	24	3	1
408.0825	-0.159036444	19	230788.43	16	26	3	1
409.2783	0.269010181	4.5	8039727.68	41	24	3	1

410.098	-0.524020813	18	230235.98	18	26	3	1
412.1136	-0.642783518	17	143848.03	20	26	3	1
414.1294	-0.277449435	16	57987.43	22	26	3	1
418.067	0.203555931	21	82882.18	14	27	3	1
420.0827	0.321603445	20	110641.48	16	27	3	1
421.2785	0.736093174	5.5	106901.87	41	25	3	1
422.0986	0.912346905	19	85641.62	18	27	3	1
424.1142	0.790118003	18	67881.51	20	27	3	1
426.1294	-0.269636334	17	51848.13	22	27	3	1
430.067	0.197876184	22	83696.75	14	28	3	1
432.0825	-0.150202775	21	148378.58	16	28	3	1
433.2786	0.946505062	6.5	51109.21	41	26	3	1
434.098	-0.49504926	20	178896.87	18	28	3	1
435.2939	0.138067655	5.5	3852620.9	43	26	3	1
436.1137	-0.378112262	19	126579.72	20	28	3	1
437.3096	0.251766775	4.5	4690758.18	45	26	3	1
438.1294	-0.262251221	18	75989.59	22	28	3	1
442.0674	1.097345184	23	50473.5	14	29	3	1
444.083	0.979772759	22	83331.62	16	29	3	1
446.0987	1.087428696	21	74751.87	18	29	3	1
448.1141	0.524643441	20	69050.31	20	29	3	1
449.3096	0.245042676	5.5	139211.38	45	27	3	1
450.1297	0.411214981	19	56669.77	22	29	3	1
452.0518	1.183715303	25	45316.75	12	30	3	1
454.0674	1.06834474	24	43834.49	14	30	3	1
456.0828	0.515476841	23	99690.81	16	30	3	1
458.0983	0.185768024	22	126315.82	18	30	3	1
460.1137	-0.358389548	21	146097.21	20	30	3	1
462.1295	-0.032242044	20	89493.38	22	30	3	1
463.3255	0.777207988	5.5	1945050.99	47	28	3	1
465.3408	0.021704523	4.5	1865174.22	49	28	3	1
468.0827	0.288624294	24	47688.3	16	31	3	1
470.0984	0.393747507	23	72481.41	18	31	3	1
472.1144	1.133413017	22	58411.33	20	31	3	1
477.3408	0.021158887	5.5	99606.31	49	29	3	1
480.0823	-0.551780088	25	59986.45	16	32	3	1
482.0979	-0.653186405	24	101231.75	18	32	3	1
484.1134	-0.960311269	23	142643.81	20	32	3	1
486.1298	0.586469225	22	80203.01	22	32	3	1
490.0662	-1.458780379	27	50508.57	14	33	3	1
491.3566	0.325832709	5.5	614139.53	51	30	3	1
492.0833	1.493855	26	41565.78	16	33	3	1
493.3723	0.425844924	4.5	107251.41	53	30	3	1
495.1063	0.525342005	24.5	75906.85	19	33	3	1
496.1144	1.078583039	24	41621.94	20	33	3	1
504.0825	-0.12874875	27	51486.8	16	34	3	1

505.3725	0.811481299	5.5	82702.38	53	31	3	1
506.0976	-1.214981563	26	83179.76	18	34	3	1
508.1137	-0.324533557	25	84929.44	20	34	3	1
510.1291	-0.813322868	24	101042.86	22	34	3	1
512.1452	0.068535256	23	45608.48	24	34	3	1
516.082	-1.094592297	28	49137.36	16	35	3	1
519.388	0.50078194	5.5	129251.8	55	32	3	1
522.1301	1.12060319	25	41030.58	22	35	3	1
526.0661	-1.549042483	30	59219.43	14	36	3	1
528.0817	-1.637812073	29	66016.54	16	36	3	1
530.0981	-0.216752286	28	63393.37	18	36	3	1
533.4041	1.33126263	5.5	63744.18	57	33	3	1
543.0124	0.478994816	35.5	51645.75	7	38	3	1
543.106	-0.073466316	28.5	114859.75	19	37	3	1
544.1148	1.718574108	28	44462.29	20	37	3	1
544.3952	-0.670284667	7	68789.96	56	34	3	1
545.1219	0.385418529	27.5	103633.6	21	37	3	1
547.1381	1.389230719	26.5	126365.06	23	37	3	1
547.4196	1.023164996	5.5	66903.96	59	34	3	1
554.0977	-0.929257641	30	44069.03	18	38	3	1
556.1132	-1.195618455	29	75926.23	20	38	3	1
558.1292	-0.564205962	28	101246.45	22	38	3	1
560.1442	-1.72258918	27	95040.54	24	38	3	1
569.1224	1.247712067	29.5	125253.21	21	39	3	1
571.1376	0.455407101	28.5	117601.57	23	39	3	1
573.1541	1.936830165	27.5	81851.96	25	39	3	1
575.1681	-0.938681219	26.5	60272.26	27	39	3	1
579.1996	-0.586843983	24.5	52323.79	31	39	3	1
580.1133	-0.973774347	31	85402.53	20	40	3	1
584.1448	-0.624673492	29	95058.37	24	40	3	1
586.1619	1.851198464	28	53055.69	26	40	3	1
589.4667	1.204649652	5.5	49525.38	65	37	3	1
591.106	-0.067500579	32.5	50804.26	19	41	3	1
593.1228	1.871622669	31.5	94118.24	21	41	3	1
595.1376	0.437041978	30.5	114406.59	23	41	3	1
597.1528	-0.318008958	29.5	103836.94	25	41	3	1
600.082	-0.94137046	35	53273.93	16	42	3	1
603.482	0.596704152	5.5	111686.08	67	38	3	1
604.1132	-1.100620328	33	81791.48	20	42	3	1
605.4977	0.677294526	4.5	5098216.39	69	38	3	1
606.1295	-0.024582205	32	83575.51	22	42	3	1
615.1065	0.748001109	34.5	49487.8	19	43	3	1
617.1222	0.826579277	33.5	66183.66	21	43	3	1
619.137	-0.548989664	32.5	75816.91	23	43	3	1
621.1535	0.821214754	31.5	75509.38	25	43	3	1
623.1693	1.05926383	30.5	52075.71	27	43	3	1

628.113	-1.376979634	35	72551.78	20	44	3	1
631.1384	1.679666109	33.5	65084.04	23	44	3	1
631.5133	0.570217928	5.5	234160.71	71	40	3	1
633.529	0.647326745	4.5	9966396.25	73	40	3	1
634.162	1.868768887	32	48933.64	26	44	3	1
637.0892	-1.867709855	37.5	45912.57	17	45	3	1
641.1215	-0.296199619	35.5	107537.04	21	45	3	1
643.1374	0.093448158	34.5	90740.3	23	45	3	1
645.1541	1.720677138	33.5	74297.35	25	45	3	1
647.1691	0.710942978	32.5	97186.07	27	45	3	1
651.1994	-0.829084843	30.5	52916.94	31	45	3	1
652.113	-1.326302118	37	59533.69	20	46	3	1
654.129	-0.787152985	36	46391.96	22	46	3	1
657.1542	1.841428128	34.5	64527.34	25	46	3	1
659.5446	0.545983087	5.5	228138.41	75	42	3	1
660.1765	0.053167603	33	50308.52	28	46	3	1
661.5604	0.771056419	4.5	10070947.77	77	42	3	1
664.2087	1.40784262	31	46839.5	32	46	3	1
665.1212	-0.736556945	37.5	49548	21	47	3	1
667.1371	-0.359596122	36.5	64274.5	23	47	3	1
669.1527	-0.433234259	35.5	58879.92	25	47	3	1
671.1687	0.089545304	34.5	63946.75	27	47	3	1
678.1288	-1.054223396	38	54873.96	22	48	3	1
680.1443	-1.271640298	37	55373.96	24	48	3	1
683.1673	-1.961301971	35.5	94994.42	27	48	3	1
684.1753	-1.702630914	35	78953.48	28	48	3	1
687.5755	-0.058029987	5.5	163286.46	79	44	3	1
689.1225	1.17555435	39.5	50395.05	21	49	3	1
689.5915	0.449686719	4.5	7035275.46	81	44	3	1
691.1368	-0.781176141	38.5	62187.38	23	49	3	1
695.1683	-0.488946107	36.5	60616.44	27	49	3	1
700.2078	0.050127979	34	59085.25	32	49	3	1
702.2228	-0.875647247	33	50406.64	34	49	3	1
703.1376	0.369913508	39.5	45134.49	23	50	3	1
715.6071	0.363467739	5.5	70470.4	83	46	3	1
717.6229	0.571470384	4.5	2979082.76	85	46	3	1
727.136	-1.842705534	41.5	45401.41	23	52	3	1
740.2402	1.53342353	35	58930.96	36	52	3	1
741.153	0.013627416	41.5	64637.56	25	53	3	1
743.1672	-1.937514479	40.5	59147.32	27	53	3	1
745.6542	0.549987146	4.5	770967.23	89	48	3	1
758.1911	-1.338578943	40	64345.79	30	54	3	1
765.1528	-0.24818566	43.5	55043.73	25	55	3	1
769.1831	-1.546963473	41.5	56811.22	29	55	3	1
773.685	-0.116197147	4.5	123934.27	93	50	3	1
782.1919	-0.274740688	42	81313.49	30	56	3	1

787.1368	-0.685903174	46.5	56346.5	23	57	3	1
798.2227	-0.895613918	41	48048.26	34	57	3	1
819.2014	1.782348764	44.5	93672.11	31	59	3	1
827.1689	0.314446157	47.5	54025.35	27	60	3	1
829.7478	0.132690939	4.5	66919.48	101	54	3	1
833.215	-0.707980032	44.5	46428.72	33	60	3	1
849.5274	-1.400659159	22.5	78258.68	73	58	3	1
856.2086	0.97534738	47	67073.71	32	62	3	1
857.7793	0.361515033	4.5	145566.82	105	56	3	1
863.1703	1.923262643	50.5	50111.18	27	63	3	1
867.2923	-1.775522815	41.5	51119.66	43	62	3	1
872.2382	-0.991585948	46	48968.25	36	63	3	1
874.1612	0.440536791	52	67302.48	26	64	3	1
878.1928	0.780125429	50	62816.43	30	64	3	1
885.2454	-1.683033306	46.5	50371.25	37	64	3	1
885.8106	0.350074958	4.5	246736.83	109	58	3	1
898.161	0.206087815	54	52645.29	26	66	3	1
904.2075	-0.292963642	51	84271.73	32	66	3	1
911.1701	1.6024476	54.5	53353.97	27	67	3	1
913.1832	-1.193515933	53.5	51322.11	29	67	3	1
913.8419	0.339336712	4.5	274144.67	113	60	3	1
915.6685	-1.026462126	17.5	49820.45	91	62	3	1
916.2077	-0.070835462	52	50684.95	32	67	3	1
920.2409	1.994155725	50	98477.13	36	67	3	1
926.1917	-0.447963218	54	50986.54	30	68	3	1
941.8733	0.435409071	4.5	180774.58	117	62	3	1
944.2396	0.566699705	52	60647.54	36	69	3	1
948.2708	0.458835387	50	47639.67	40	69	3	1
962.2862	0.192354453	50	51579.19	42	70	3	1
966.3177	0.398523336	48	49583.8	46	70	3	1
967.2307	-0.558191235	54.5	84671.47	35	71	3	1
969.9042	0.0104134	4.5	73990.54	121	64	3	1
991.6999	-0.846928883	21.5	51806.8	95	68	3	1
1004.241	1.926931612	57	47898.82	36	74	3	1
1007.734	1.44889707	20.5	53217.47	99	69	3	1
1026.319	1.641889867	53	52590.06	46	75	3	1
1029.246	-0.767454347	58.5	46597.14	37	76	3	1
1062.225	1.77467374	63	53077.42	34	79	3	1
1082.031	1.857712941	4.5	61439.88	137	72	3	1
1082.192	-0.568198559	67	46703.34	30	81	3	1
1083.037	-0.290756398	4	51926.15	138	72	3	1
1109.054	1.29398701	5	59990.23	140	74	3	1
1109.497	-0.351420417	46.5	46109.29	69	80	3	1
1109.779	0.279425005	25.5	53329.64	105	77	3	1
1110.062	0.819865083	4.5	89107.88	141	74	3	1
1110.881	0.256643217	18	75897.26	118	76	3	1

b)

Mass (m/z)	Deviation (ppm)	DBE	Intensity (AU)	H	C	O	S
93.9728	-2.28683	1	2333199.5	2	1	3	1
94.9807	-1.47293	0.5	21072048.92	3	1	3	1
104.9653	1.048919	2.5	10841.24	1	2	3	1
106.9809	0.561783	1.5	7320835.87	3	2	3	1
108.9966	1.010124	0.5	2712140.45	5	2	3	1
119.9887	0.292528	2	62284.8	4	3	3	1
120.9965	0.083473	1.5	1697099.17	5	3	3	1
123.0121	-0.32436	0.5	1404597.5	7	3	3	1
132.9965	0.075942	2.5	24202.44	5	4	3	1
137.0276	-1.38585	0.5	200061.48	9	4	3	1
143.9884	-1.83973	4	56083.07	4	5	3	1
149.0276	-1.27426	1.5	63754.96	9	5	3	1
154.9809	0.38779	5.5	38011.86	3	6	3	1
155.9886	-0.41606	5	604358.14	4	6	3	1
156.9964	-0.57262	4.5	6660420.78	5	6	3	1
168.9966	0.651493	5.5	39442.63	5	7	3	1
170.0043	-0.08764	5	20941188.06	6	7	3	1
171.0121	-0.23332	4.5	2905824.73	7	7	3	1
180.9965	0.055802	6.5	3698739.11	5	8	3	1
183.0122	0.328394	5.5	511433348.8	7	8	3	1
184.0198	-0.8961	5	20270630.3	8	8	3	1
185.0276	-1.02633	4.5	1067406.08	9	8	3	1
191.0746	-0.73217	1.5	382828.95	15	8	3	1
191.9883	-1.90063	8	13177.45	4	9	3	1
192.9962	-1.5021	7.5	21178.78	5	9	3	1
193.0902	-0.98348	0.5	24524.73	17	8	3	1
194.004	-1.62316	7	593122.87	6	9	3	1
195.0122	0.308186	6.5	20162138.29	7	9	3	1
196.0199	-0.33109	6	8197732.64	8	9	3	1
197.0279	0.558804	5.5	56843043.79	9	9	3	1
198.0355	-0.5802	5	1712417.22	10	9	3	1
199.0432	-1.20526	4.5	432480.05	11	9	3	1
205.0902	-0.92593	1.5	62430.93	17	9	3	1
207.0121	-0.19274	7.5	8243816.36	7	10	3	1
208.0196	-1.75416	7	150988.63	8	10	3	1
209.0278	0.048319	6.5	8199712.19	9	10	3	1
210.0352	-1.97538	6	1510177.41	10	10	3	1
211.0433	-0.6629	5.5	4734852.41	11	10	3	1
213.0588	-1.36066	4.5	252480.22	13	10	3	1
219.1059	-0.6385	1.5	24954.98	19	10	3	1
220.0198	-0.74948	8	445468.79	8	11	3	1
221.0278	0.045696	7.5	1054986.51	9	11	3	1

222.0354	-0.96786	7	75002.56	10	11	3	1
223.0434	-0.17889	6.5	1401504.48	11	11	3	1
224.0509	-1.62865	6	145168.54	12	11	3	1
225.059	-0.39945	5.5	3809232.44	13	11	3	1
226.0666	-1.39295	5	275629.01	14	11	3	1
227.0743	-1.93725	4.5	473793.94	15	11	3	1
231.1059	-0.60535	2.5	5917.13	19	11	3	1
233.0281	1.330743	8.5	478860.27	9	12	3	1
233.1217	0.043325	1.5	12195.94	21	11	3	1
235.0436	0.681151	7.5	343335.91	11	12	3	1
237.0591	0.042605	6.5	563291.92	13	12	3	1
239.0748	0.251386	5.5	3755693.56	15	12	3	1
241.0904	0.041893	4.5	253578.98	17	12	3	1
243.0125	1.481819	10.5	34732.24	7	13	3	1
244.0198	-0.67576	10	89207.49	8	13	3	1
246.0356	-0.06056	9	97960.91	10	13	3	1
247.0437	1.052851	8.5	241655.09	11	13	3	1
247.1374	0.243185	1.5	82320.34	23	12	3	1
248.0515	0.947788	8	25259.55	12	13	3	1
248.9653	0.44223	14.5	14001.6	1	14	3	1
249.0591	0.040553	7.5	155630.22	13	13	3	1
250.0668	-0.45948	7	28662.05	14	13	3	1
251.0746	-0.5572	6.5	399589.43	15	13	3	1
253.0904	0.039907	5.5	2191250.63	17	13	3	1
254.0039	-1.63344	12	13149.49	6	14	3	1
254.0978	-1.63283	5	193353.8	18	13	3	1
255.0125	1.41209	11.5	64827.38	7	14	3	1
255.1062	0.627582	4.5	167141.55	19	13	3	1
256.0197	-1.03468	11	57768.26	8	14	3	1
258.0353	-1.22037	10	191381.64	10	14	3	1
260.0508	-1.78772	9	214390.12	12	14	3	1
261.1532	0.804509	1.5	27942.19	25	13	3	1
262.0664	-1.96477	8	409036.77	14	14	3	1
263.075	0.988692	7.5	42291.37	15	14	3	1
265.0906	0.79256	6.5	118004.94	17	14	3	1
266.0977	-1.935	6	88060.24	18	14	3	1
267.0122	0.225083	12.5	16746.63	7	15	3	1
267.1062	0.599387	5.5	1125887.07	19	14	3	1
268.0202	0.877173	12	39860.53	8	15	3	1
268.1135	-1.36099	5	28165.31	20	14	3	1
269.1219	0.780688	4.5	222767.53	21	14	3	1
270.0357	0.315144	11	81872.34	10	15	3	1
272.0518	1.966909	10	26310.25	12	15	3	1
275.0752	1.672636	8.5	118117.73	15	15	3	1
275.169	1.308652	1.5	19781.89	27	14	3	1
276.0822	-1.32171	8	28458.89	16	15	3	1

277.0902	-0.68534	7.5	53883.88	17	15	3	1
278.0977	-1.8515	7	73529.97	18	15	3	1
279.1062	0.573617	6.5	56823.45	19	15	3	1
280.0198	-0.58889	13	42178.5	8	16	3	1
280.1134	-1.65968	6	23668.86	20	15	3	1
281.1218	0.391645	5.5	756496.02	21	15	3	1
282.0354	-0.76196	12	111914.98	10	16	3	1
282.1294	-0.40726	5	7508.94	22	15	3	1
283.1375	0.56545	4.5	516173.71	23	15	3	1
284.051	-0.93258	11	142584.37	12	16	3	1
284.9655	1.088203	17.5	9179.16	1	17	3	1
286.0665	-1.45036	10	184593	14	16	3	1
287.0751	1.254377	9.5	383271.16	15	16	3	1
288.0821	-1.61377	9	205003.24	16	16	3	1
289.1845	0.726526	1.5	19092.33	29	15	3	1
290.0977	-1.77492	8	75511.22	18	16	3	1
292.0198	-0.56469	14	7330.09	8	17	3	1
292.1136	-0.90684	7	19278.07	20	16	3	1
293.1219	0.716767	6.5	51729.26	21	16	3	1
294.0358	0.629516	13	76175.96	10	17	3	1
294.1293	-0.73063	6	6620.16	22	16	3	1
295.1373	-0.13519	5.5	793677.3	23	16	3	1
296.0512	-0.21922	12	42624.42	12	17	3	1
297.1531	0.370516	4.5	11867614.88	25	16	3	1
299.0752	1.538411	10.5	146237.85	15	17	3	1
301.0908	1.362049	9.5	112263.21	17	17	3	1
301.1845	0.69758	2.5	8529.25	29	16	3	1
302.0977	-1.70441	9	30423.04	18	17	3	1
303.1065	1.517951	8.5	27513.2	19	17	3	1
303.2001	0.528034	1.5	72062.85	31	16	3	1
304.0195	-1.52918	15	22336.87	8	18	3	1
304.1133	-1.85753	8	21093.8	20	17	3	1
305.2159	1.016003	0.5	11244.4	33	16	3	1
306.0356	-0.04869	14	57885.23	10	18	3	1
307.1376	0.846852	6.5	46177.14	23	17	3	1
308.0511	-0.5353	13	116286.65	12	18	3	1
309.1531	0.356134	5.5	1218202.66	25	17	3	1
310.0668	-0.37057	12	78343.28	14	18	3	1
311.169	1.15725	4.5	48840611.29	27	17	3	1
312.0822	-1.16924	11	105808.24	16	18	3	1
314.0978	-1.32092	10	71517.5	18	18	3	1
317.0278	0.031858	15.5	8987.97	9	19	3	1
317.122	0.977858	8.5	8356.7	21	18	3	1
317.2157	0.347083	1.5	26008.9	33	17	3	1
318.0359	0.89644	15	22813.17	10	19	3	1
319.044	1.75556	14.5	47274.12	11	19	3	1

319.1376	0.81501	7.5	8342.32	23	18	3	1
319.2311	-0.43824	0.5	7295.71	35	17	3	1
320.0513	0.10967	14	32294.41	12	19	3	1
321.153	0.031449	6.5	22855.27	25	18	3	1
323.1687	0.185971	5.5	1063626.52	27	18	3	1
325.1846	0.953613	4.5	43762883.49	29	18	3	1
328.1133	-1.72166	10	15200.99	20	19	3	1
330.0361	1.469841	16	33860.29	10	20	3	1
331.2315	0.785252	1.5	39994.32	35	18	3	1
332.0513	0.105707	15	52290.68	12	20	3	1
333.1533	0.930804	7.5	18038.31	25	19	3	1
333.247	0.330386	0.5	10613.4	37	18	3	1
334.0669	-0.0446	14	52885.6	14	20	3	1
335.1689	0.776027	6.5	22542.29	27	19	3	1
337.1845	0.623101	5.5	732218.37	29	19	3	1
339.2001	0.471993	4.5	29010380.77	31	19	3	1
340.1133	-1.66091	11	9058.05	20	20	3	1
343.1375	0.466577	9.5	11387.02	23	20	3	1
344.0519	1.845946	16	39830	12	21	3	1
345.153	0.029262	8.5	6184.68	25	20	3	1
345.247	0.318902	1.5	57353.77	37	19	3	1
346.0675	1.690713	15	16795.02	14	21	3	1
347.2626	0.173068	0.5	23502.41	39	19	3	1
351.2003	1.025342	5.5	62486.73	31	20	3	1
353.2158	0.594821	4.5	142053.32	33	20	3	1
354.0359	0.805286	18	7611.31	10	22	3	1
356.0513	0.098581	17	30707.62	12	22	3	1
357.1535	1.428239	9.5	6947.24	25	21	3	1
358.0669	-0.04161	16	44497.42	14	22	3	1
359.1689	0.724172	8.5	62786.83	27	21	3	1
359.2628	0.723983	1.5	232395.49	39	20	3	1
361.2784	0.581546	0.5	48368.69	41	20	3	1
364.1133	-1.55144	13	12805.88	20	22	3	1
365.2159	0.849088	5.5	35034.18	33	21	3	1
367.2315	0.708273	4.5	79992.21	35	21	3	1
368.0513	0.095367	18	17964.41	12	23	3	1
370.0672	0.770401	17	16118.09	14	23	3	1
373.1844	0.295028	8.5	440891.4	29	22	3	1
373.2783	0.294954	1.5	77785.49	41	21	3	1
375.2	0.160181	7.5	29639.27	31	22	3	1
375.294	0.426599	0.5	34558.68	43	21	3	1
378.0353	-0.83299	20	7175.48	10	24	3	1
379.2314	0.42217	5.5	25040.17	35	22	3	1
380.0513	0.092356	19	19583.78	12	24	3	1
381.2472	0.813384	4.5	99219.65	37	22	3	1
382.0673	1.007939	18	22109.14	14	24	3	1

387.2003	0.93001	8.5	20635.54	31	23	3	1
387.2941	0.671583	1.5	102315.52	43	22	3	1
389.3098	0.796539	0.5	50416.6	45	22	3	1
392.052	1.87501	20	11169.09	12	25	3	1
393.247	0.279977	5.5	8403.54	37	23	3	1
394.0672	0.723481	19	12772.95	14	25	3	1
395.2626	0.152051	4.5	14189.72	39	23	3	1
401.3097	0.523536	1.5	135061.61	45	23	3	1
403.3253	0.39695	0.5	56359.48	47	23	3	1
404.0516	0.82935	21	6245.62	12	26	3	1
406.0667	-0.52922	20	18169.64	14	26	3	1
407.2627	0.393113	5.5	10014.45	39	24	3	1
409.2784	0.513343	4.5	33817.13	41	24	3	1
413.3097	0.508336	2.5	6415.23	45	24	3	1
415.3255	0.867032	1.5	216112.79	47	24	3	1
417.3412	0.98265	0.5	81190.38	49	24	3	1
421.2786	0.973466	5.5	6238.92	41	25	3	1
423.2943	1.086952	4.5	12375.7	43	25	3	1
428.052	1.717318	23	5820.86	12	28	3	1
429.2471	0.489462	8.5	6083.06	37	26	3	1
429.3409	0.25644	1.5	122144.16	49	25	3	1
430.0664	-1.19726	22	17394.26	14	28	3	1
431.3566	0.371155	0.5	81827.43	51	25	3	1
435.2944	1.286717	5.5	20293.95	43	26	3	1
437.3097	0.480438	4.5	1988029.08	45	26	3	1
443.3567	0.586661	1.5	114260.66	51	26	3	1
444.0823	-0.59651	22	12508.27	16	29	3	1
445.3726	1.145334	0.5	109120.09	53	26	3	1
448.0195	-1.03768	27	7153.55	8	30	3	1
449.31	1.135298	5.5	23729.22	45	27	3	1
451.3255	0.797873	4.5	2617857.06	47	27	3	1
452.051	-0.586	25	7224.87	12	30	3	1
455.3564	-0.08762	2.5	7275.4	51	27	3	1
457.2788	1.334199	8.5	15325.34	41	28	3	1
457.3726	1.115284	1.5	120449.42	53	27	3	1
459.2944	1.219481	7.5	651176.31	43	28	3	1
459.3883	1.219231	0.5	142314.86	55	27	3	1
465.3411	0.666393	4.5	684957.93	49	28	3	1
467.3566	0.342565	3.5	6840.47	51	28	3	1
468.0829	0.715899	24	6583.79	16	31	3	1
471.3881	0.763915	1.5	159293.97	55	28	3	1
473.3101	1.289009	7.5	11082.68	45	29	3	1
473.4038	0.86628	0.5	161982.11	57	28	3	1
477.3415	1.487616	5.5	9168.48	49	29	3	1
478.0662	-1.4954	26	13236.43	14	32	3	1
479.3569	0.959829	4.5	1146401.86	51	29	3	1

480.0819	-1.38497	25	14425.82	16	32	3	1
482.0978	-0.86061	24	19727.41	18	32	3	1
485.4039	1.050879	1.5	107258.4	57	29	3	1
487.4197	1.354276	0.5	130349.37	59	29	3	1
490.0662	-1.45878	27	6310.72	14	33	3	1
491.075	0.529655	26.5	15165.39	15	33	3	1
493.3726	1.033905	4.5	79552.2	53	30	3	1
497.122	0.623791	23.5	50024.57	21	33	3	1
497.4032	-0.38178	2.5	6537.69	57	30	3	1
499.4196	1.121503	1.5	98611.89	59	30	3	1
501.0596	1.018044	28.5	8247.73	13	34	3	1
501.4353	1.216709	0.5	73903	61	30	3	1
503.0739	-1.66953	27.5	11454.75	15	34	3	1
504.0819	-1.31903	27	5297.95	16	34	3	1
506.0975	-1.41257	26	15386.99	18	34	3	1
507.3882	0.906802	4.5	276096.17	55	31	3	1
513.4351	0.798738	1.5	81362.23	61	31	3	1
515.4507	0.698612	0.5	50979	63	31	3	1
517.0894	-1.91437	27.5	17617.95	17	35	3	1
519.1053	-1.42533	26.5	32974.64	19	35	3	1
521.122	0.595063	25.5	48072.17	21	35	3	1
523.4198	1.452183	3.5	9818.56	59	32	3	1
525.4347	0.019222	2.5	6332.5	61	32	3	1
527.4507	0.682718	1.5	62272.22	63	32	3	1
529.0909	0.964107	28.5	27382.01	17	36	3	1
529.4666	1.152293	0.5	27894.39	65	32	3	1
531.1057	-0.63999	27.5	36187.36	19	36	3	1
532.1136	-0.49783	27	16607.4	20	36	3	1
533.1225	1.519541	26.5	35800.24	21	36	3	1
534.1286	-1.71288	26	19849.63	22	36	3	1
536.1442	-1.7997	25	10514.56	24	36	3	1
541.4663	0.572704	1.5	35543.72	65	33	3	1
543.1052	-1.54648	28.5	24889.43	19	37	3	1
543.4822	1.030578	0.5	15459.14	67	33	3	1
545.1211	-1.08214	27.5	36949.83	21	37	3	1
547.1364	-1.71785	26.5	24484.76	23	37	3	1
555.4822	1.008314	1.5	14634.89	67	34	3	1
557.1225	1.454081	28.5	28581.11	21	38	3	1
557.4973	0.018117	0.5	6507.39	69	34	3	1
558.1292	-0.56421	28	13578.17	22	38	3	1
563.0757	1.705102	32.5	5534.08	15	39	3	1
565.0908	0.725725	31.5	14214.81	17	39	3	1
566.0982	-0.02632	31	14442.08	18	39	3	1
567.1065	0.811312	30.5	17315.17	19	39	3	1
569.4977	0.720109	1.5	17623.75	69	35	3	1
571.1366	-1.29549	28.5	27920	23	39	3	1

572.1463	1.983937	28	10832.4	24	39	3	1
573.1528	-0.33133	27.5	28036.72	25	39	3	1
575.1686	-0.06937	26.5	6390.75	27	39	3	1
575.3557	-1.28599	12.5	372785.12	51	37	3	1
577.0914	1.750332	32.5	5846.93	17	40	3	1
579.1056	-0.75962	31.5	11019.95	19	40	3	1
581.1216	-0.1547	30.5	26001.99	21	40	3	1
582.1288	-1.22808	30	13463.09	22	40	3	1
583.514	1.816755	1.5	7690.78	71	36	3	1
584.1458	1.08723	29	19214.85	24	40	3	1
585.1537	1.213529	28.5	26074.06	25	40	3	1
586.0658	-1.90234	35	6245.74	14	41	3	1
586.1598	-1.73144	28	11181.97	26	40	3	1
587.1679	-1.26011	27.5	12745.46	27	40	3	1
589.09	-0.66187	33.5	20175.48	17	41	3	1
591.1053	-1.25172	32.5	12807.28	19	41	3	1
595.1368	-0.90719	30.5	21930.32	23	41	3	1
597.1529	-0.15055	29.5	22352.35	25	41	3	1
604.1131	-1.26615	33	13160.63	20	42	3	1
605.1218	0.181947	32.5	16097.41	21	42	3	1
606.1286	-1.50941	32	7101.29	22	42	3	1
607.1375	0.263697	31.5	21543.71	23	42	3	1
608.1443	-1.42219	31	11416.41	24	42	3	1
609.1537	1.165717	30.5	17784.96	25	42	3	1
611.1693	1.080062	29.5	13154.39	27	42	3	1
613.0912	1.321339	35.5	7337.75	17	43	3	1
613.1843	0.016471	28.5	11908.85	29	42	3	1
615.1054	-1.04031	34.5	15611.97	19	43	3	1
617.1209	-1.27997	33.5	16836.95	21	43	3	1
619.1363	-1.6796	32.5	15732.65	23	43	3	1
621.1539	1.465178	31.5	24963.84	25	43	3	1
624.1754	-1.70609	30	6402.81	28	43	3	1
627.1072	1.849926	35.5	10334.94	19	44	3	1
628.1132	-1.05857	35	7751.09	20	44	3	1
631.1382	1.362778	33.5	15190.7	23	44	3	1
634.1614	0.922637	32	7880.19	26	44	3	1
637.1843	0.015851	30.5	8270.88	29	44	3	1
639.1065	0.719912	36.5	7097.69	19	45	3	1
640.1148	1.460834	36	6840.82	20	45	3	1
643.1365	-1.30594	34.5	16331.6	23	45	3	1
645.152	-1.53436	33.5	12488.47	25	45	3	1
647.1681	-0.83425	32.5	7629.16	27	45	3	1
648.1754	-1.64292	32	7210.56	28	45	3	1
651.1992	-1.13621	30.5	5884.31	31	45	3	1
669.1524	-0.88156	35.5	12905.8	25	47	3	1
672.1777	1.837464	34	7430.7	28	47	3	1

673.1841	-0.28209	33.5	6532.81	29	47	3	1
675.2006	0.977636	32.5	7080.86	31	47	3	1
678.129	-0.75929	38	9133.38	22	48	3	1
683.1673	-1.9613	35.5	11916.82	27	48	3	1
685.0905	0.160709	41.5	5929.28	17	49	3	1
687.2007	1.106083	33.5	8159.32	31	48	3	1
695.168	-0.9205	36.5	7184.02	27	49	3	1
710.1907	-1.99228	36	9552.82	30	50	3	1
713.2168	1.696682	34.5	6108.4	33	50	3	1
719.1682	-0.61168	38.5	11162.79	27	51	3	1
725.1225	1.117192	42.5	7221.15	21	52	3	1
728.1447	-0.63847	41	10874.23	24	52	3	1
735.2001	0.217764	37.5	7090.21	31	52	3	1
745.1832	-1.46259	39.5	6343.57	29	53	3	1
754.1606	-0.28495	42	15076.34	26	54	3	1
756.1751	-1.805	41	16690.33	28	54	3	1
764.1437	-1.91704	44	9661.12	24	55	3	1
765.1528	-0.24819	43.5	6110.99	25	55	3	1
766.16	-1.06361	43	5977.72	26	55	3	1
776.1448	-0.47014	45	8115.8	24	56	3	1
784.2077	-0.08276	41	6414.21	32	56	3	1
792.1757	-0.96557	44	8651.25	28	57	3	1
802.1611	0.355415	46	10177.1	26	58	3	1
835.6999	-1.00502	8.5	46269.73	95	55	3	1
848.2381	-1.13753	44	6680.94	36	61	3	1
854.1915	-0.71986	48	8329.56	30	62	3	1
855.1995	-0.51438	47.5	6358.64	31	62	3	1
863.7323	0.301135	8.5	58091.3	99	57	3	1
891.7626	-0.8297	8.5	11985.84	103	59	3	1
893.2155	-0.10065	49.5	6064.56	33	65	3	1
898.1599	-1.01864	54	6883.31	26	66	3	1
941.2164	0.860695	53.5	5976.65	33	69	3	1
948.269	-1.43936	50	6427.31	40	69	3	1
993.2453	-1.60071	55.5	7737.8	37	73	3	1
1028.239	-0.54939	59	5882.74	36	76	3	1

c)

Mass (m/z)	Deviation (ppm)	DBE	Intensity (AU)	H	C	O	S
106.9809	0.561782839	1.5	11985.31	3	2	3	1
156.9964	-0.572624268	4.5	85848.85	5	6	3	1
171.0121	-0.233316769	4.5	8278.56	7	7	3	1
297.1531	0.370516211	4.5	75644.46	25	16	3	1
311.1688	0.514512002	4.5	295070.3	27	17	3	1
325.1843	0.031059311	4.5	275892.6	29	18	3	1
339.2	0.177181635	4.5	68174.87	31	19	3	1
155.9886	-0.416055872	5	50009.39	4	6	3	1
170.0043	-0.087644834	5	193261.1	6	7	3	1

184.0199	-0.352679124	5	94614.58	8	8	3	1
198.0355	-0.580198668	5	3177.24	10	9	3	1
183.0121	-0.218018324	5.5	5819652	7	8	3	1
197.0278	0.051261804	5.5	409289	9	9	3	1
211.0435	0.284775495	5.5	38650.67	11	10	3	1
225.0591	0.044877103	5.5	28921.2	13	11	3	1
239.0749	0.66966506	5.5	29204.32	15	12	3	1
253.0905	0.435022444	5.5	11567.52	17	13	3	1
267.1064	1.348153715	5.5	2877.42	19	14	3	1
295.1375	0.542459317	5.5	6298.23	23	16	3	1
309.1532	0.679598797	5.5	9376.64	25	17	3	1
323.1686	-0.12346495	5.5	9393.61	27	18	3	1
196.0199	-0.331088724	6	86192.03	8	9	3	1
210.0353	-1.499269541	6	4897.37	10	10	3	1
180.9965	0.055802187	6.5	100900.8	5	8	3	1
195.0121	-0.204602647	6.5	306767.7	7	9	3	1
209.0278	0.048318934	6.5	172641.7	9	10	3	1
223.0436	0.717797394	6.5	17874.88	11	11	3	1
237.0591	0.042605411	6.5	5164.54	13	12	3	1
194.0042	-0.592254869	7	5085.41	6	9	3	1
207.0122	0.290321138	7.5	36289.6	7	10	3	1
221.0279	0.498127408	7.5	22893.32	9	11	3	1
235.0436	0.681150685	7.5	3652.35	11	12	3	1
233.028	0.901609203	8.5	3000.88	9	12	3	1
252.9961	-1.541128101	12.5	5215.16	5	14	3	1
250.9804	-1.752723436	13.5	3296.72	3	14	3	1
